# An explainable machine learning framework for cross-dataset chronic kidney disease risk prediction using hybrid data balancing

**DOI:** 10.1371/journal.pone.0362320

**Published:** 2026-11-05

**Authors:** Jarin Tasnim Mumu, Ismail Hossain, Kazi Md. Tanvir Anzum

**Affiliations:** 1 Department of Industrial Engineering and Management, Khulna University of Engineering & Technology, Khulna, Bangladesh; 2 Department of Mechanical Engineering, Khulna University of Engineering & Technology, Khulna, Bangladesh; Healthcare and Technology Institute (HCT Academy), PHILIPPINES

## Abstract

Chronic kidney disease (CKD) is a major global health problem, and early detection is important for reducing complications and improving treatment. Machine learning has shown strong potential for CKD prediction, but many existing studies rely on single datasets, face severe class imbalance, and provide limited interpretability. These challenges reduce model reliability and make cross-dataset use in real clinical settings more difficult. Therefore, this study aimed to develop an interpretable and adaptable machine learning framework for CKD risk prediction across different datasets. The proposed hybrid explainable machine learning (HXAI-ML) framework used two public CKD datasets: the UCI CKD Risk Factor Prediction dataset as the source dataset and the Kaggle CKD dataset as the target dataset. After preprocessing, label binarization, imputation, and standard scaling, seven balancing methods and six classifiers were evaluated. A target adaptation setting was applied by using 10% of the target dataset for calibration and 90% for blind testing. SHAP, LIME, and Permutation Importance Analysis were used to explain the model predictions. Among all tested combinations, RO + TL + ETC achieved the highest overall accuracy among the evaluated target-domain adaptation configurations and was therefore selected as the primary model under the accuracy-based selection criterion. On the 1,494-sample blind holdout it obtained 91.97% accuracy, 92.24% precision, 99.64% recall, 95.80% F1-score, and 67.52% ROC-AUC. However, its specificity of 5.74% and balanced accuracy of 52.69% indicate substantial class-wise imbalance in predictive performance. The findings demonstrate the methodological feasibility of cross-dataset target-domain adaptation and explainable machine learning under limited target-domain data. However, the relatively low ROC-AUC values and low specificity for the non-CKD class indicate limited discriminative ability. Therefore, the proposed framework should be regarded as an exploratory cross-dataset evaluation rather than a clinically deployable screening or decision-support system.

## 1. Introduction

Chronic kidney disease (CKD) affects more than 850 million people worldwide, and approximately 1.5 million people die from CKD annually. This amounts to 1 death in every 20 seconds around the world. Cases of CKD are supposed to increase by 2050 [1]. CKD is another condition that poses a big economic challenge in the world. Approximately 93.1 million individuals worldwide in Europe have CKD. Kidney replacement therapy is implemented on approximately 750,000 patients. Within a year, the number of people dying as a result of CKD is approximately 210,000 [1]. According to World Kidney Day reports, there are severe impacts of kidney disease. There are at least 2.4 million deaths on kidney related disorders each year. Kidney disease is among the leading causes of death worldwide and is one of the causes of mortality showing substantial growth globally [2]. The development of kidney issues can have a number of causes. It may result in Acute Kidney Injury as a result of dehydration or low blood flow. The AKI can also be caused by infections, injuries, specific medicines, and kidney stones. Such conditions can also rapidly impair the functionality of kidneys [3]. Kidneys can also be damaged because of autoimmune diseases and exposure to harmful environmental factors. These factors can be identified early and lead to the correct prevention and treatment. Severe kidney complications can be diminished with the early treatment [4]. National kidney foundation classifies CKD in five stages. These are stages that are reliant on kidney abnormalities and their slowed rate of filtration. The Glomerular Filtration Rate is the measure of the availability of the kidneys to filter the blood [5]. CKD is a significant reason for heart disease and a serious cause of premature mortality [6]. Individuals are supposed to understand that CKD is treatable and manageable. Vital diseases are observable and treatment can be delayed. With appropriate management, the significant issues with kidney might be avoided [7]. CKD will lead to kidney failure without treatment. Thus, timely diagnosis of CKD is very critical. Machine Learning is useful in the identification of CKD at its initial stages. It uses patient medical data by identifying trends and risks. Such models examine the tests of blood as well as the history of the patients. They assist in monitoring kidney damage without showing serious symptoms [8].

Federated Learning is a privacy-conscious machine learning method. Without sending raw data, models flow through many locations. Between systems only model updates are exchanged. Sensitive medical data are secured during the training process [9]. Without patient-record sharing, hospitals will be able to establish stronger models. They also exchange model parameters as opposed to raw information. This would enhance teamwork and preserve the privacy of patients [10]. ML has already been put in healthcare in order to enhance the diagnosis of CKD through the analysis of complex medical data and identifying patterns that can be detected by doctors without using particular equipment [8,11]. Clustering algorithms like k means identify latent patterns of patient data. Such patterns allow finding subgroups of patients to treat them specifically [8,11]. Deep learning models are also used to enhance the level of diagnosis of CKD. Convolutional Neural Network is an analysis of medical imaginations like ultrasound or MRI. Sequential data on patients are recycled via Neural Networks. These models describe the complex medical relationships that is not a linear association when using medical data. They aide in identifying the irregularities of the images and temporal health data [8,11]. The study of CKD prediction has continued to develop over the last several years. A great deal of research features sophisticated machine learning codes. They are a Random Forest and XGBoost. Neural networks are also popular. Researchers combine ensemble learning models with feature selection. Such a method enhances the accuracy of prediction and reliability of the model [8]. Through these steps, by following them systematically, researchers were able to come up with strong and correct prediction models in case of chronic kidney disease. Combination of feature selection and proper machine learning algorithms enhanced better model result and high prediction accuracy. Hyper parameter optimization also enhances the model performance. Parameters such as grid search and random search are used to optimize. These methods determine the optimal model set ups. Raised models are embedded in clinical decision systems as part of some studies. These systems are used to ensure that doctors make enhanced and faster decisions whenever making medical choices [8,11,12].

### 1.1 Technical challenges

While doing the experiment, several technical difficulties appeared. They are following below:

1. Severe Class Imbalance: The CKD datasets have a much larger number of CKD cases than non -CKD cases. This imbalance will place models in favor of the majority class.
2. **Oversampling and Intersecting Samples:** Superimposition of clinical data results in duplicated data around the limits of classes. Such noisy samples cause confusion among models and absence of accurate prediction.
3. Elements of Minimization of Target Data Adaptation: A trained model that has been trained using one dataset distribution could not work on a different dataset distribution. Customization of models using little new data is still technically difficult.
4. **Nonexistence of Model Interpretability:** A lot of machine learning models are black boxes in the medical predictions. Automated predictions cannot be trusted before the clinical is clearly explained to clinicians. [13]

### 1.2 Problem statement

Despite the increasing use of machine-learning methods for CKD prediction, several technical challenges limit their reliable application across datasets. In the target CKD dataset considered in this study, the class distribution is highly imbalanced, with 1,524 CKD samples compared with only 135 non-CKD samples. Such severe imbalance can bias conventional classifiers toward the majority CKD class and reduce their ability to correctly identify non-CKD cases. In addition, differences between the source and target datasets create a cross-dataset adaptation challenge, as a model trained on one dataset may not directly generalize to another dataset with different sample distributions and characteristics. The limited representation of the minority class further restricts reliable target-domain adaptation. Moreover, high predictive performance alone is insufficient for clinical applications when the underlying model decisions cannot be clearly interpreted. Therefore, there is a need for a framework that simultaneously addresses class imbalance, cross-dataset adaptation, and model interpretability. These challenges motivate the development of the proposed HXAI-ML framework, which combines data-balancing strategies, source-domain model optimization, target-domain adaptation, and explainable AI techniques to provide more transparent CKD predictions. Despite the increasing application of machine-learning techniques for CKD prediction, several challenges remain, including severe class imbalance, limited availability of representative target-domain samples, variation between source and target datasets, and insufficient interpretability of model predictions. Conventional learning approaches may become biased toward the majority class and may not adequately distinguish CKD from non-CKD cases. Furthermore, models that provide high predictive performance without transparent explanations can limit their usefulness in clinical decision-making. Therefore, there is a need for a robust framework that integrates effective class-imbalance handling, cross-domain adaptation, and explainable AI to improve CKD prediction while providing transparent and clinically interpretable model decisions.

### 1.3 Research question

**RQ1:** Which data-balancing technique, including oversampling, SMOTE, and hybrid balancing approaches, provides the most effective handling of class imbalance for CKD prediction?

**RQ2:** Can XAI techniques explain how ML model predicts CKD risk?

**RQ3:** Can target-domain adaptive generalization enable CKD models to transfer reliably across different medical datasets with only limited labeled target data?

To address these questions, this study has some objectives. They are described in the next sub-section.

### 1.4 Objectives

The study intended to address the challenges faced by the objectives following below:

- To identify the most effective data balancing technique for improving CKD prediction on imbalanced datasets.
- To develop an interpretable machine learning framework using XAI techniques for CKD risk prediction.
- To evaluate the ability of target adapting cross-dataset learning to generalize CKD prediction across different medical datasets.

The remainder of the article is organized as follows: Section 2 reviews the research literature regarding the prediction of chronic kidney disease. Section 3 provides an overview of the suggested methodology and explains it. Section 4 provides an evaluation study of the suggested framework. Section 5 evaluates our method, explains the study topics, and discusses the key findings, limitations, and recommendations. Section 6 concludes the paper with a summary of the paper’s contributions.

## 2. Literature review

Chronic Kidney Disease (CKD) is a condition which gradually deteriorates the performance of kidneys and is colossal health challenge in the world platform. The markers in the blood and urine estimated glomerular filtration rate (eGFR) and urine albumin-to-creatinine ratio (uACR)) tend to enable the physicians to diagnose CKD and also give insights on the progression. These are useful in the classification of CKD though they are not that specific and they can sometimes fail to capture the complex biology of the force behind the disease [14].

### 2.1 Chronic kidney disease clinical risk determinants

In the recent research, the inflammation has been found to be significant in CKD. In case the filters are broken, a type of protein Albumin is commonly released into the urine by the blood. This will not be allowed to happen in a healthy kidney. CKD is the consequence of high blood pressure, which may also occur due to CKD [15]. Imflammatory biomarkers are indicative of deterioration of kidney function and end-stage kidney disease. They include Tumor Necrosis Factor Receptor 1, Tumor Necrosis Factor Receptor 2 and Interleukin-6 [16,17]. Such inflammatory mechanisms also lead to the scar tissue and the destruction of the blood vessels in the kidney [14]. eGFR figure illustrates that your blood is being cleansed by your kidney. When the eGFR levels exceed 90, then the kidney is fine. When the eGFR is below 60, it implies that you have CKD [18]. Another test ordered by the doctor is urine test to determine the functionality of the kidneys since the kidneys produce urine. When the urine is filled with blood and protein, that is an indication that your kidney is not functioning properly [19]. Doctor takes blood pressure since Blood pressure range indicates the manner in which your heart is pushing blood. When the amount of eGFR at less than one hundred and fifty, then the patient has end-stage kidney disease. Now all that is available is treatment: (i) dialysis, (ii) kidney transplant. In case of dialysis failure, the physician is left with the only option, i.e., transplantation of kidney. But it is so very costly [20]. CKD is also caused by oxidative stress. When the body is over-production of reactive oxygen species (ROS) then the cells are destroyed, inflammation develops, and scar tissue is deposited in the kidneys. Measures of such oxidative stress like urinary 8 oxiogo, F2 isoprostanes have also been studied to establish the possibility of PC measurement of early kidney problems and predict long-term albumin in the urine and kidney disease [14].

### 2.2 Machine learning in CKD prediction

Machine-learning methods have increasingly been applied to CKD prediction, with studies evaluating different classification algorithms and feature-selection strategies. Polat et al. [21] developed a Support Vector Machine (SVM)-based CKD prediction model using the UCI CKD database and reported an accuracy of 98.5%. Their findings demonstrated the importance of selecting informative features for improving predictive performance [21]. Medicine is being considered the field where new computer programs that learn data are being applied. They are able to read a plethora of patient records, identify patterns and assist in predicting and diagnosing diseases [22]. Almasoud et al. [23] have experimented with various algorithms in computers, such as the Logistic Regression, Random Forest, SVM and Gradient Boosting, to identify CKD. They discovered that Gradient Boosting was the most accurate and correctly identified 99.1% [23]. Random Forest would easily be applied in CKD prediction since it can deal with complex medical data. Several algorithms were used to come up with a prediction model of end stage kidney disease. Han et al. expressed that, Han et al. [24] evaluated several algorithms for predicting end-stage kidney disease and reported that Random Forest achieved the highest performance, with an accuracy of approximately 93.97%. [24]. Qin et al. [25] developed a composite model combining Logistic Regression and Random Forest for CKD progression prediction and reported an accuracy of 99.83%, suggesting that combining complementary models may improve predictive performance. [25]. Farjana et al. [26] compared multiple machine-learning algorithms for CKD prediction and identified LightGBM as the best-performing algorithm, achieving approximately 99% accuracy and a high AUC [26]. Similarly, Raihan et al. experimented an XGBoost predictive model with SHAP explanation and used to predict CKD. It was used to identify the accurate explanation to a 99.16% and demonstrates the significance of explainable models in healthcare [27]. Overall, previous studies demonstrate the strong predictive potential of machine-learning algorithms for CKD prediction, particularly ensemble and boosting methods. However, many existing studies rely on relatively small datasets or evaluate models within a single dataset, which may limit their generalizability to different populations and data distributions. These limitations highlight the need to investigate model robustness across datasets and to address class-imbalance and cross-dataset adaptation challenges [22,28].

### 2.3 Explainable AI in CKD risk prediction

In addition to predictive performance, the interpretability of machine-learning models has become an important consideration in healthcare applications. Many predictive models operate as black boxes, making it difficult for clinicians to understand the basis of individual predictions [29]. The Explainable Artificial Intelligence or XAI is a new research area which acquires a growing method in AI [30] of making these predictions more understandable. XAI methods including SHapley Additive explanations and Local Interpretable Model-agnostic Explanations are employed by researchers in explaining the model of CKD prediction. Ghosh and Khandoker [31] evaluated Logistic Regression, Random Forest, and XGBoost models for CKD prediction and examined the clinical features contributing to their predictions. Important features included creatinine, glycated hemoglobin (HbA1C), and patient age [31]. These predictions were explained using SHAP and LIME. SHAP was also used by Arjaria et al. when explaining CKD detection models. Their model was based on Logistic Regression to predict CKD. The analysis demonstrated the relevance of strides that define the presence of CKD. They consisted of serum creatinine, hemoglobin, albumin, and random blood glucose analyzes [32]. Gupta et al. [33] proposed a hybrid CKD prediction framework that combined ensemble learning with the Grey Wolf Optimizer and reported an accuracy of approximately 98.75%. This study demonstrates the potential of combining multiple predictive models with optimization techniques to improve classification performance. In contrast, the present study focuses on the effect of data-balancing strategies and cross-dataset target-domain adaptation rather than optimization of a hybrid ensemble. The proposed framework also evaluates performance on a blind target-domain holdout after adaptation, providing a different perspective on model generalization across datasets. [33]. Elshewey et al. [34] investigated an Extra Trees-based CKD prediction approach incorporating feature-selection and SHAP-based interpretation. The reported accuracy of approximately 99.9% demonstrates the strong predictive capability of ensemble tree-based models under the evaluated dataset conditions. However, the study primarily evaluates prediction and interpretability within its dataset, whereas the present study examines whether a source-domain model can be adapted to a distinct target dataset and subsequently evaluated on a blind target-domain holdout. [34]. Rezk et al. [35] investigated a different framework incorporating a Generative Adversarial Network for handling missing data together with Prototypical Networks and Model-Agnostic Meta-Learning. The reported prediction accuracy of approximately 99.99% indicates the potential of combining advanced representation-learning and adaptation strategies for CKD prediction. Nevertheless, the methodology differs from the present framework in its treatment of class imbalance, target-domain adaptation, and model evaluation. Our study systematically compares seven balancing configurations and six classifiers and evaluates the adapted model using an independent 90% target-domain blind holdout [35]. Elshewey et al. [34] further investigated explainable machine-learning approaches for CKD prediction and demonstrated the use of XAI techniques to identify influential clinical features contributing to model predictions. Their findings highlighted features such as hemoglobin, serum creatinine, and hypertension as important predictors. The use of SHAP and LIME provided both feature-level and instance-level interpretations of the predictive models. However, this approach primarily addresses model interpretability, whereas the present study additionally investigates the effect of multiple class-balancing strategies and cross-dataset target-domain adaptation on predictive performance [34]. SHAP and LIME are among the commonly used post-hoc XAI methods for interpreting machine-learning predictions. SHAP provides feature-attribution values based on Shapley-value principles, while LIME generates locally interpretable approximations around individual instances [36,37]. These approaches can provide both global insights into feature importance and local explanations of individual predictions. XAI allows scientists and physicians to understand how a machine-learning model arrives at a guess, indicating the most significant factors that took part in the choice [38]. Medical pictures of kidneys are also processed with the help of XAI. In 2021, Bayram and others applied explainable deep-learned models to discuss kidney images and identify parts that depict cysts or other disease. They demonstrated that XAI may provide images aiding physicians to arrive at decisions [39]. Recent CKD prediction research has achieved high reported predictive performance using ensemble learning, optimization, advanced learning architectures, missing-data handling, and XAI. However, these studies differ in how they address class imbalance and generalization across datasets. The present study complements this literature by systematically evaluating seven balancing configurations—Random Oversampling, SMOTE, their combinations with Tomek Links, Instance Hardness Threshold, and related configurations—across six classifiers. The selected source-domain model is subsequently adapted using 10% of the target dataset and evaluated on a blind 90% target-domain holdout. This design allows the study to examine cross-dataset target-domain adaptation rather than relying solely on within-dataset performance. In addition, SHAP, LIME, and PIA are incorporated to examine model behavior and prediction explanations. Therefore, the primary contribution of the present work is not simply achieving high within-dataset accuracy, but integrating class-imbalance handling, target-domain adaptation, blind cross-dataset evaluation, and explainability within a unified CKD prediction framework. Overall, the literature indicates that XAI can improve the interpretability of CKD prediction models by identifying influential features and providing explanations at both global and individual levels. However, existing studies primarily focus on model development and explanation within individual datasets, leaving comparatively less attention to the joint challenges of class imbalance, cross-dataset target-domain adaptation, and explainability. These limitations motivate the integrated approach investigated in this study. A comparative summary of the reviewed studies, together with their reported limitations and the way the present study addresses them, is presented in Table 1.

**Table 1:** Comparative analysis of related work on CKD risk prediction and mitigation.

Ref	Application Focuus	Main Goal	Method Category	Data Balancing Technique	XAI Integrating	Limitations	How this study addresses it
[35]	CKD IoT Prediction	Few-Shot	ML, GANs	Not Specified	Yes (SHAP, LIME)	Focuses in limited cross- domain depth	Using few-shot personalization, adding PIA
[40]	CKD Forecasting	ML Modeling	RFC, ETC, ANN	SMOTE	Yes (SHAP, LIME)	Single Dataset	Cross dataset training, XAI implementation
[41]	CKD Progression	Short-term Prediction	XGBC, LR	No	No	Shifting domain externally	Few-shot personalization, XAI
[42]	CKD Prediction	Detection in community	ML Classifiers	No	Yes (SHAP)	Limited Personalization	Multiple dataset few-shot generalization, XAI
[43]	CKD Prediction	ML-CKDP modeling	ML	No	No	Focuses on preprocessing	Adding data balancing technique + cross dataset personalization
[44]	CKD domain adaptation	Generalization	RDANN	Not Specified	No	Limited feature	Doing Few-shot, XAI
[45]	CKD cross domain adaptation	Few-shot learning	DKD, pseudo-labels	No	No	Mismatch category	Doing Few-shot, XAI
[46]	CKD Progression	Validation externally	RFC	No	No	Risky data leakage	Train, personalized test split doing
[47]	CKD Federated	Equitable models	Distributed FL	No	No	Syncing time high	Adding Few-shot in cross dataset
[48]	CKD Federated	Preserving with privacy maintaining	RFC, XGBC	No	No	Predictive only	Combining Few-shot personalization with XAI
**This Study**	**Cross dataset CKD Risk personalization assessment**	**Generalization or personalization**	**RFC, DTC, ETC, GBC, MLP, XGBC**	**RO, SMOTE, RO + TL, SMOTE+TL, RO + IHT, SMOTE+IHT, IHT**	**Yes (SHAP, LIME, PIA)**	**Addressing past gaps**	**Cross-dataset, training, 10% target adaptive personalization on another dataset and 90% blind test on the remaining, comparative XAI analysis**

## 3. Proposed methodology

This study offers a HXAI-ML framework for chronic kidney disease risk prediction structures. This study is approached a novel cross-dataset target adaptive learning mechanism where clinical CKD data from one dataset (source) is integrated with the second dataset (target). This model shows the combination of Ensemble Machine Learning (EML) models and both local and global explainability techniques ensuring the predictive interpretability. The overall system consisting of 6 chronological instances: (i) dataset acquisition, (ii) data preprocessing and feature engineering, (iii) data balancing with necessary techniques, (iv) ensemble model training, (v) performance metrics investigation, (vi) explainability using different interpretable methods of explainable AI (XAI). The detailed chronological methodology is described in Fig 1. This methodology is aimed to predict performance with transparency and methodologically rigorous for highly impactful modeling.

**Fig 1:**
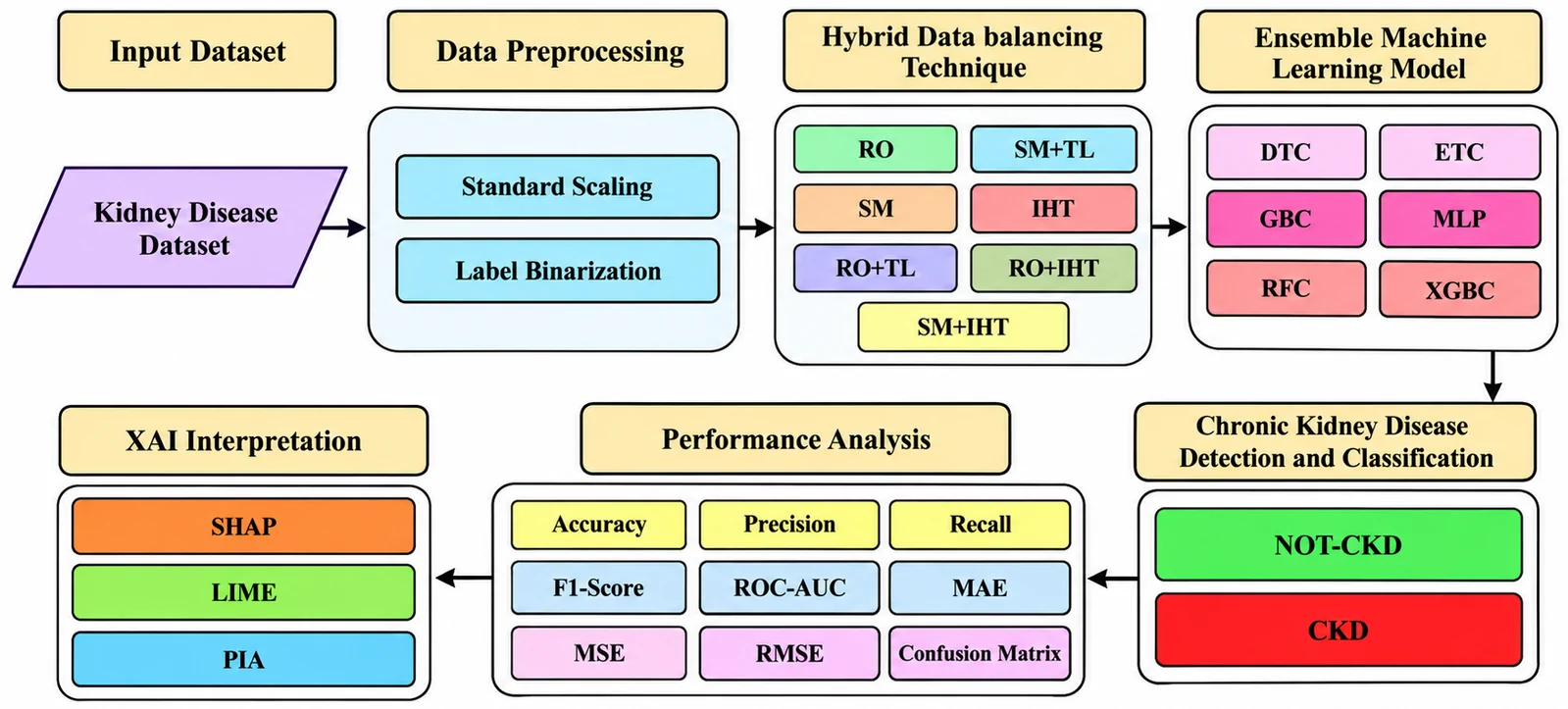
Proposed Hybrid XAI-ML Approach for CKD Risk Detection.

### Dataset acquisition

The experimental datasets were built by two independent (CKD) datasets within a cross-dataset target adaptive learning framework. First of all, The UCI CKD Risk Factor Prediction dataset (Enam Medical College, Bangladesh) was used as a source dataset to train the model. Chronic Kidney Disease dataset by Rabie El Kharoua (Kaggle) was used as a target dataset for target-domain adaptation and evaluation. Both datasets that include clinically relevant attributes that are associated with kidney function and patient health indicators. Firstly, for model development, the datasets underwent preprocessing measures such as data cleaning, data standardization through standard scaling and label binarization for class. The final dataset that was cleaned and validated included 200 UCI observations and 1659 Kaggle observations. In both datasets, there were ten attributes that were selected that can be used to analyze the prediction of chronic kidney disease.

### Data preprocessing and feature engineering

The datasets were prepared using a consistent preprocessing procedure to ensure methodological consistency. First, the target variable was binarized, with non-CKD cases labeled as 0 and CKD cases labeled as 1. The common attributes shared by the source and target datasets were retained to ensure consistency across domains. The source and target datasets were preprocessed before the target-domain adaptation stage. Missing values were handled using imputation, followed by feature standardization using StandardScaler. All preprocessing parameters, including imputation values and scaling parameters, were derived exclusively from the corresponding training or adaptation data and subsequently applied to the evaluation data. After preprocessing, the target dataset was partitioned into a 10% adaptation subset and a 90% blind holdout subset. The 10% adaptation subset was used for target-domain adaptation and calibration, whereas the 90% holdout subset was reserved exclusively for final blind evaluation. No model fitting, hyperparameter tuning, threshold selection, or performance evaluation used information from the blind holdout before the final evaluation.

### Standard scaling

Standard Scaler is a technique that is used to scale numeric data to have a standard normal distribution. Changes the data such that the average value of the data is 0, and the standard deviation is 1. It does by eliminating the average from each value and dividing by the deviation. The outcome of this is the data is centered to zero and the variability is made the same.


$$\begin{array}{c} m_{ij}^{scaled}=\frac{m_{ij}-\mu_{j}}{\sigma_{j}} \end{array}$$ (1)

where $\mu_{j}$ and $\sigma_{j}$ are training-set mean and standard deviation.

We addressed the imbalance between the Safe and Risk groups by balancing the groups using the different balancing techniques. It was done only on the training dataset. This ensured that the training set was balanced, but that the test set remains in the original order so that this would provide an unbiased evaluation.

### Data balancing techniques

To mitigate the imbalance in our data, we used numerous balancing methods. These methods ensure that both the minority and the majority groups are of roughly equal importance and as such this model is trained with balanced data. We examined the suggested proposed methods.

### Random oversampling (RO)

Random oversampling (RO) is replicating examples of the minority class so as to match the balance of the majority class. This technique is useful for the minority class because it makes multiple copies of examples that already exist to alleviate the class imbalance. However, existing samples can be repeatedly learnt, which can cause severe overfitting. The model can just remember what they see rather than generalizing what they see in the information. As such, the model might do exceptionally well when using the data available in training phase and do poorly with unknown data.

### Synthetic minority oversampling technique (SMOTE)

Synthetic Minority Over-sampling Technique (SMOTE) generates synthetic minority-class samples by interpolating between existing minority-class instances to alleviate class imbalance [49]. The synthetic sample formation can be written as:


$$\begin{array}{c} m_{\text{new}\;}=m_{\text{min}}+\lambda \cdot \left(m_{\text{neighbor}\;}-m_{\text{min}}\right) \end{array}$$ (2)

where,

$m_{\text{new }}$= synthetic instance,

$m_{\text{min }}=$ minority class instance,

$m_{\text{neighbor }}=$ randomly selected neighbor of $m_{\text{min }},$

λ = arbitrary digit between 0 and 1

### Random oversampling with Tomek Links (RO + TL)

It is a hybrid model which is used to determine class imbalance in combination of naïve oversampling with data cleaning. The hybrid balancing approach will initially increase the samples of minority classes so that the imbalance in classes is minimized. Then it filters the samples with noise to enhance clarity of the decision boundaries. Let $m_{i}$ and $m_{j}$ be a PIA of instances belonging to different classes; they form a TL if:


$$\begin{array}{c} \left(m_{i},m_{j}\right)<c\left(m_{i},m_{k}\right)\;\text{and}\;c\left(m_{j},m_{i}\right)<c\left(m_{j},m_{l}\right) \end{array}$$ (3)

where:

- $c\left(m_{i},m_{j}\right)$ is the distance between $m_{i}$ and $m_{j}$,
- $m_{k}$ and $m_{l}$ are instances from other classes.

In our study, we proposed RO + TL as best approach because of the fixing the severe class imbalance of the original distribution. After applying this strategy, dataset become balanced and computation was perfectly done.

### SMOTE with Tomek Links (SMOTE+TL)

This combination works as SMOTE creates synthetic minority examples, clears the overlaps between classes using TL. It normalizes the data but gives better parting between classes. This process helps eliminate uncertain data points and helps improve the separation between classes. By combining synthetic sample generation and noise and overlap removal.

### Instance hardness threshold (IHT)

Instance Hardness Threshold is an undersampling method that is used to deal with instances of imbalance between classes. It eluted hard samples that may be in interclass regions which are frequently close together. The resulting cleaner dataset, as a result of this process, contributes to the machine learning models acquiring meaningful patterns. The models hence use more informative and representative samples as they train.

### Random oversampling with instance hardness threshold (RO + IHT)

IHT is a measure of the hardness of the instances that means how difficult is it to correctly classify them by the model. RO + IHT method employs random oversampling, excludes crude instances with the help of a threshold to correct class distribution. This hybrid strategy not only helps in improving the balance of classes by the effect of oversampling. Though it also helps in improving the quality of data by removing the problematic instances. Therefore, the model gets capable of learning clear decision boundaries which gives for improved stability. It also lowered misclassification and improved generalization results of unseen data.

### SMOTE with instance hardness threshold (SMOTE+IHT)

In this approach, the synthetic minority examples are removed if they are hard to classify based upon IHT. The SMOTE+IHT method is a synthetically driven data enhanced with challenging sample filtering. This is done to increase the class balance as well as the quality of the dataset. This allows the model to learn more prominent rules of decision-making which results into better accuracy in new data.

### Proposed approach (RO + TL)

Among the above methods, RO + TL comes out the best method producing the best presentation in removing class imbalance and enhancing model simplification. Fig 2 illustrates the flow chart of the study framework we used to develop the predictive decision from the dataset.

**Fig 2:**
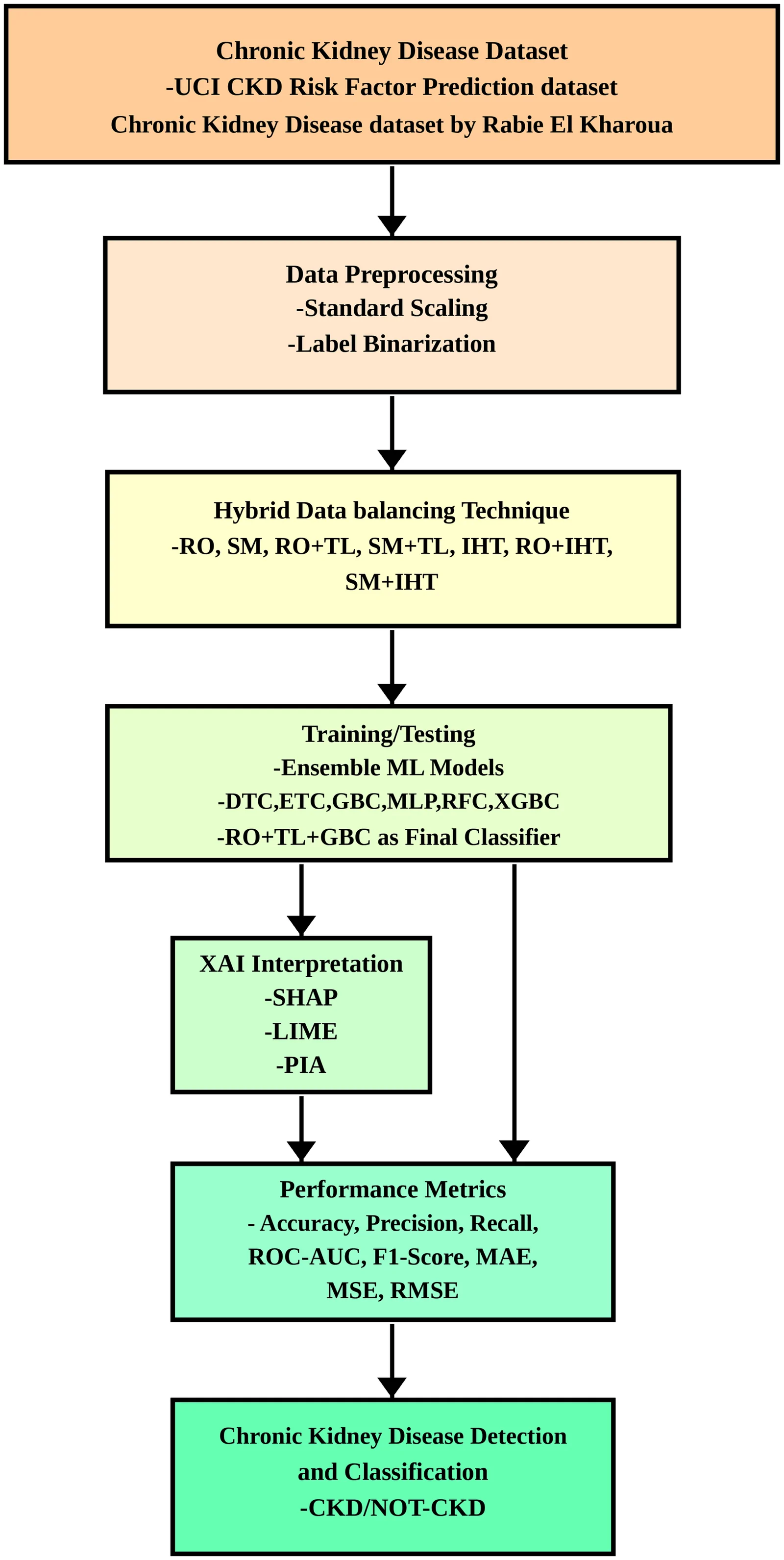
Schematic diagram of hybrid XAI-ML approach predicting CKD risk.

### Ensemble machine learning (EML) models

To enhance the stability of prediction and model robustness several ensemble-based classifiers were used. Such models are projections that are combined together like concerted instruments in an elaborate orchestra. The EML framework used five models: Decision Tree Classifier (DTC), Random Forest (RFC), Extra Trees (ETC), Gradient Boosting (GBC), and the sharper, faster Extreme Gradient Boosting (XGBC).

### Decision tree classifier (DTC)

DTC is a model that creates a tree structure. With the tree structure, it split the data into branches based on features to predict the outputs. DTC is easily interpretable to understand and visualize. The Mini impurity at a node is:


$$\begin{array}{c} M=\text{1}-\sum_{i=\text{1}}^{D}\;n_{i}^{\text{2}} \end{array}$$ (4)

where, $n_{i}$ = proportion of class i instances,

D = total number of classes.

### Random forest classifier (RFC)

An ensemble method building multiple decision trees during the training period. Then they merge their predictions by averaging for regression or majority vote for classification. In this mechanism, each tree is trained on a random subset of features creating diversity. Mathematically, the final prediction $\hat{g}$ of RFC is given by:


$$\begin{array}{c} \hat{g}=\frac{\text{1}}{T}\sum_{t=\text{1}}^{T}\;h_{t}(m) \end{array}$$ (5)

where $h_{t}(m)$ = the prediction of the t-th tree,

T = total number of trees.

### Extra trees classifier (ETC)

ETC is similar kind of Random Forest Classifier, which ensembles de-correlated decision trees. It generates threshold for each feature instead of finding the discriminative threshold for each feature. Then it preferences the top among the random splits. Also, it reduces variance and make the model faster than others.

### Gradient boosting classifier (GBC)

GBC is a boosting method which sequentially builds additive model trees. Each new tree modifies the errors made by the previous trees. It is slightly slower model technique but gives higher accuracy. The disadvantage of the technique is the overfitting tendency because of the not tuning correctly. The update rule for the model prediction $\hat{F}(j)$ at iteration m is:


$$\begin{array}{c} {\hat{F}}_{m}(j)={\hat{F}}_{m-\text{1}}(j)+\eta \cdot h_{m}(j) \end{array}$$ (6)

where, η = learning rate

$h_{m}(j)$ = m-th base learner.

### Extreme gradient boosting classifier (XGBC)

XGBC is an advanced and enhanced version of GBC. It enhances both the speed and performance. It offers good controlling over overfitting by initializing regularization. The regularized objective function is:


$$\begin{array}{c} L(\theta )=\sum_{i=\text{1}}^{N}\;l\left(s_{i},{\hat{s}}_{i}\right)+\Omega (f_{k}) \end{array}$$ (7)

Where, N = Number of samples,

$l\left(s_{i},{\hat{s}}_{i}\right)=$ loss function,

$s_{i}=$ The true label (actual target value) for the i-th training example.

${\hat{s}}_{i}$= The predicted label (model output) for the i-th training example,

$\omega_{j}$ = model parameters,

Ω = regularization term.

### Multilayer perception (MLP)

MLP is a neural network which artificially design the model. It is a class which set the complex nonlinear relationship between input and output features. MLP creates multiple layers of the neural networks to represent hierarchical feature. So, in total, MLP consists of three parts:

1. Input Layer
2. Hidden Layers
3. Output Layer

If the input feature vector represent as:


$$\begin{array}{c} a={\left[a_{\text{1}},a_{\text{2}},a_{\text{3}},\ldots \ldots ,a_{n}\right]}^{T} \end{array}$$

Where n denotes as the number of input features. Predicted class can be expressed:


$$\begin{array}{c} \hat{q}=\sigma (p)=\frac{\text{1}}{\text{1}+e^{-p}} \end{array}$$ (8)

where:

- $\hat{q}=$ predicted probability of CKD
- p= linear combination from the final layer

The predicted class label is obtained as:


$$\begin{array}{c} {\hat{q}}_{\text{class }}=\left\{ \begin{array}{cc} \text{1}, & \hat{q}\geq \text{0}.\text{5}\\ \text{0}, & \hat{q}<\text{0}.\text{5} \end{array}\right. \end{array}$$

In this work, a number of machine learning models were used for classification of chronic kidney disease (CKD) using clinical and laboratory features. These models were able to capture nonlinear relationships and also overlapping variables. These models also identified complicated feature interactions found typically within medical data. Means to check the severe class imbalance, RO + TL technique was used to balance the training dataset and remove unclear samples near decision boundaries. This approach allowed the models to study the differences between CKD and non-CKD cases. Among the methods evaluated, RO + TL with Extra Tree Classifier (ETC) gave the best result under the accuracy-optimized selection criterion. The models were evaluated with cross dataset validation with target adaptive personalization. In this methodology, 10% of the target data was employed for adaptation and 90% was kept for blind testing in order to simulate an actual application scenario. Performance was assessed in terms of accuracy, precision, recall, F1-score, ROC-AUC, MAE, MSE and RMSE. A threshold calibration was used in order to maintain a balanced trade-off between sensitivity and specificity.

### Explainable artificial intelligence (XAI) techniques

The HXAI-ML framework combines three explainability methods to ensure that it is interpretable. Such methods are SHAP, LIME, and Permutation Importance Analysis to explain the model. These tools are analytical lenses that show the effects of features to predictive outcomes.

### Shapley additive explanation (SHAP)

The method is considered as a state-of-the-art technique in the field of machine learning interpretability. Its most important use is in understanding the contribution of individual elements of the data – for instance features or variables – to the prediction output of a model. SHAP computes the importance of features based on Shapley values based on the concept of cooperative game theory. This method describes predictive contribution as well as privacy sensitivity of the elements of data belonging to an individual.

### Local interpretable model-agnostic explanation (LIME)

LIME is one of the prominent XAI frameworks provided for visualizing and interpreting ML prediction approach. It aims to produce the interpretation of each individual prediction of how it approaches to the direction of the predictions. It is a local interpretation like the global model behavior. It is useful for the prediction of complicated models at a local level.

### Permutation importance analysis (PIA)

Permutation Importance Analysis is a metric of feature importance based on randomly shuffling feature values, as evaluated. The significance of every feature is ascertained by noting the change of performance with shuffling. It displays the most important factors in a descriptive way in the context of determining relevant data in a medical context. PIA consumes fewer computer power as compared with other techniques (SHAP and LIME). It directly operates with performance measures such as accuracy and F1 score, thus providing results that are easy to understand.

We used SHAP, LIME and PIA in this study for analyzing the influence of each clinical feature on the prediction model for CKD. SHAP was used to calculate the influence of each feature to the predictions of the framework. In order to explain predicted results for individual patients, LIME reduced the complicated model into an obvious local model. Permutation Importance Analysis gives the importance of each feature, merges it to the note the change in performance the feature values are randomly swapped. These methods give more obvious and less biased insights than the traditional measures of importance because they are model independent. Also, they are easy to comprehend. Using SHAP and permutation scores, we managed to define the important clinical features. These scores were also used to confirm that they indeed have an effect on the prediction of CKD. Combining explainability tools and ensemble machine learning is an important address to a challenge in medical prediction. It has a high degree of accuracy and explainability simultaneously. Ensemble models like the Gradient Boosting and Random Forest give very good prediction but often they are a black-boxes. Our way of solving this is evidenced by the combination of robust ensembles and easily understandable forms of AI. This makes the model easier to understand and yet maintain good model performance. This explanation framework enhances reliability, reproducibility as well as finding clinical dependability in the decision support system. It also aids in the earlier detection and prediction of risk of chronic kidney disease.

### Explainability analysis

Explainability analyses were performed using the final selected models after target-domain adaptation. SHAP, LIME, and Permutation Importance were applied to examine the contribution of individual features to model predictions. The reference data for LIME were drawn from the adaptation-training subset, while explanations were generated for observations in the 90% blind holdout dataset. SHAP and PIA were also applied to the blind holdout data to examine global, class-specific, and model-specific feature contributions. The explanations were interpreted as descriptions of model behavior rather than evidence of causal relationships, external validity, or clinical validity. Feature-ranking stability across independent resamples was not formally assessed; therefore, the reported rankings should not be interpreted as evidence of ranking stability.

## 4. Performance analysis

In this article, we used two different data sets. The first one is the UCI CKD Risk Factor Prediction dataset from the Enam Medical College in Bangladesh. The second one is the chronic kidney disease by Rabie El Kharoua on Kaggle. The 10% of the Kaggle data was used for target-domain adaptation and the remaining 90% were used for standard validation. The framework tests are the model that determines accuracy and stability in the various datasets that represent patients of different populations. This design replicates real-life situations in which models will have to work in various healthcare situations. [50]

### 4.1 Experimental setup

A simple overview of the experiment is provided in Table 2. The framework of the experiment has a list of datasets, balancing strategies, algorithms, evaluation metrics, and target domain adapatation strategies. From the UCI dataset, global training and parameter tuning are taken. The Kaggle one is the target. 10% is for personalization, and 90% is for blind testing. The table also shows the balancing tricks, models and how we used them.

**Table 2:** System specifications and experimental framework.

Components	Description
**Dataset Characteristics**	The two datasets of chronic kidney disease (CKD) are also used in this study, and they were obtained via varying sources. The source dataset is the dataset of UCI CKD Risk Factor Prediction dataset gathered at Enam Medical college, Bangladesh that has 200 samples and 10 attributes. Its target dataset is the chronic kidney disease dataset provided by Rabie EL Khouroua on Kaggle that has 1659 samples and 10 attributes after preprocessing. Both datasets are binary classification tasks of CKD and Non-CKD trainings.
**Data Preprocessing Pipeline**	The preprocessing of the datasets was done before model training so that the learning was consistent and reliable. The distributions of the features were normalized using standard scaling and the label binarization transformed the class labels to numerical values. This preprocessing step helped in normalization of features and lessened bias in the training of the model.
**Training and Testing**	In this study, a cross-dataset validation approach was used. Model training was performed on the UCI CKD Risk Factor Prediction dataset and the Kaggle CKD was the target domain. The target dataset was again separated into 10% target adaptive calibration set and 90% blind holdout test set, which enables the model to adapt to the new dataset distribution and thereafter the model is fully evaluated.
**Class Balancing Techniques**	A number of data balancing methods were also used to cope with class imbalance. They are Random Oversampling (RO), Synthetic Minority Oversampling Technique (SMOTE), Random Oversampling with Tomek Links (RO + TL), SMOTE with Tomek Links (SMOTE+TL), Instance Hardness Threshold (IHT), Random Oversampling with IHT (RO + IHT) and SMOTE with IHT (SMOTE+IHT). The methods were used to better class representation of minorities and better model generalization.
**Predictive Algorithms**	These are some machine learning algorithms such as Random Forest Classifier (RFC), Decision Tree Classifier (DTC), Extra Trees Classifier (ETC), Gradient Boosting Classifier (GBC), Extreme Gradient Boosting Classifier (XGBC), and Multilayer Perceptron (MLP) applied in performing CKD prediction task. The models have been chosen as such since they have the ability to induce nonlinear relationships and negatively interactive features in clinical data.
**XAI Implementation**	Three explainable artificial intelligence methods were used to increase the transparency and interpretability of models: SHAP (Shapley Additive Explanations), LIME (Local Interpretable Model-Agnostic Explanations) and Permutation Importance Analysis (PIA). These methods are capable of giving explanations on model predictions on the global and local levels and the significant contribution of meaningful clinical features.
**Performance Metrics Evaluation**	The model performance was measured based on a number of classification and error-based measures such as Accuracy, Precision, Recall, F1-Score, ROC-AUC, Mean Absolute Error (MAE), Mean Squared Error (MSE) and Root Mean Squared Error (RMSE). These measures represent an overall assessment of the classification accuracy, predictive reliability and model error.
**Tools and Libraries Used to Compute**	In the implementation, the experiment was done with Python-based scientific libraries. Scikit-learn was used to implement machine learning models, NumPy and Pandas were used to perform data manipulation, Matplotlib and Seaborn were used to perform visualization, and the Imbalanced-learn library was used to handle class imbalances.
**Best Performed Model So Far**	Among the evaluated configurations, RO + TL + ETC achieved the highest overall accuracy and CKD recall. However, its specificity for the non-CKD class was only 5.74%, indicating substantial class-wise imbalance in prediction. Therefore, the model is considered the highest-performing configuration under the accuracy-based selection criterion, rather than the most clinically balanced model.

#### Data collection.

This study has taken two public CKD data sets namely: UCI dataset from Enam Medical College, Bangladesh and Kaggle data set by Rabie El Kharoua. These are datasets that provide information on real clinical risk factors required for predicting CKD. Using two different datasets – one smaller, one larger – allows us to test whether the model can be generalized on data which may appear different. The UCI set is the basis from which training and tuning is performed. The Kaggle set is the target set for target-domain adaptation and blind testing.

#### UCI CKD risk factor prediction dataset.

This dataset contains 200 samples, 10 clinical attributes and 2 labels: CKD or not-CKD. Being a small robot, we utilized it for the main training, tuning, and testing of various balancing strategies. It gives a very nice binary classification problem for an early detection of CKD.

#### Chronic kidney disease dataset by Rabie El Kharoua.

The dataset publicly available on Kaggle, contains 1,659 samples with 10 attributes and 2 class labels. The pooled data sets are greater and more varied to realistic model testing. This variety aids in the assessment of the models on medical data that they have not encountered before. We divided it into 10% to do a couple of shots tweak of model and 90% to blind test how well the crossover domain prediction holds up. The increased size allows us to inspect the stability, adaptability and scalability in changing data.

#### Data visualization and performance metrics.

Fig 3 presents how the CKD data in the source set is imbalanced. Most of the cases are CKD (about 92%) while only about 8% are not-CKD. This imbalance meaningfully affects how the models learn and informed the choice of balancing technique used in both the main training pipeline and the target-domain adaptation stage. After balancing, the classes are evenly distributed, as shown in Fig 4, which illustrates the effect of the RO + TL technique specifically. Model performance was evaluated using a standard set of metrics: Accuracy, Precision, Recall, F1-Score, ROC-AUC, Mean Absolute Error, Mean Squared Error, and Root Mean Squared Error: which together capture classification correctness, robustness to class imbalance, and the magnitude of prediction error. Performance is reported separately for source-domain training and the independent, blind target-domain holdout evaluation.

**Fig 3:**
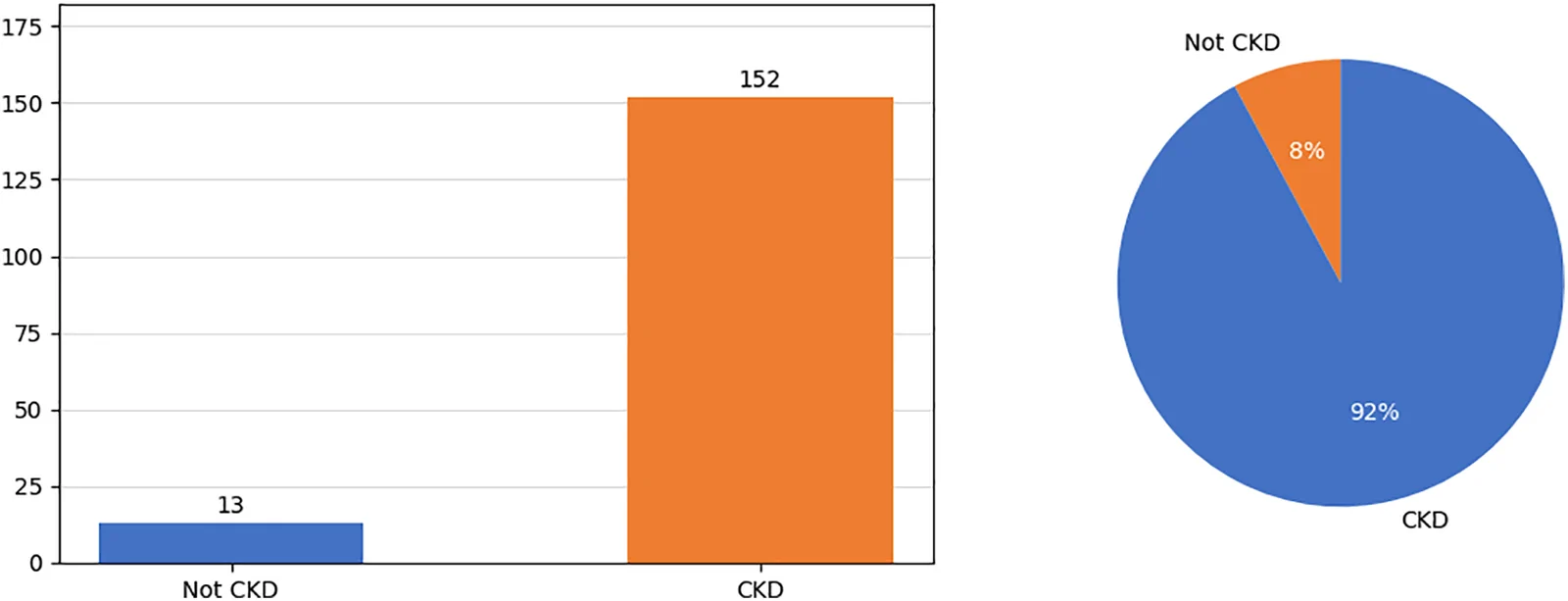
Original data distribution of each labellabel. (a) The Count of Each Label (b) The Percentage of Each Label.

**Fig 4:**
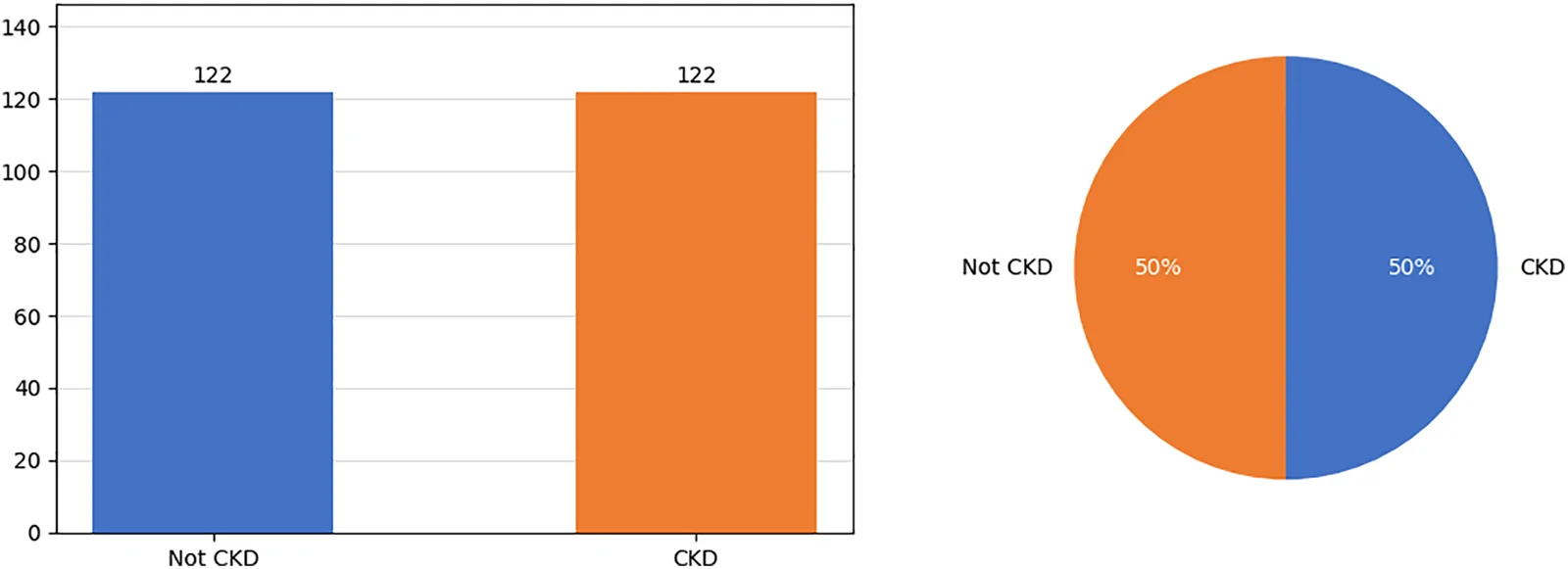
Class distribution after applying RO + TL data balancing techniquetechnique. (a) The Count of Each Label (b) Percentage of Each Label.

In the final analysis, the models were trained on the UCI CKD Risk Factor Prediction Dataset, and their hyperparameters were optimized using RandomizedSearchCV with 20 randomly sampled configurations and 5-fold stratified cross-validation. Balanced accuracy was used as the optimization criterion, with a fixed random seed of 42. We then divided the Kaggle root in 10% for small-samples-adaption and 90% for blind test. We tried some of the balancing tricks such as Random Oversampling, SMOTE, combinations with Tomek Links, Instance Hardness Threshold, mixes etc. We tested each of the mixes on a number of algorithms: Random Forest, Decision Tree, Gradient Boosting, XGBoost, Extra Trees, and a Multi-Layer Perceptron. This examination reveals the effectiveness, solidness and computational soundness of each approach to dealing with data from a variety of sources.

#### Computational environment.

All experiments were conducted in a Python-based computational environment. The hardware and software configuration used to obtain the training and prediction times reported in Table 3 is provided to ensure reproducibility. The experiments were executed using AMD EPYC 7812, 16 GB RAM, and Windows Operating System, with Python 3.14.x. The principal libraries used were scikit-learn 1.6.1, imbalanced-learn 0.14.2, XGBoost 3.4.1, NumPy 2.1.3, Pandas 2.2.3, Matplotlib 3.10.0, and Seaborn 0.13.2.

**Table 3:** Complexity analysis evaluation of ML models.

Data Balancing Technique	Model	Training Time (s)	Prediction Time (s)	Total Time (s)
RO + TL	DTC	0.004	0.001	0.005
	ETC	0.445	0.097	0.542
	RFC	0.177	0.004	0.181
	GBC	0.449	0.002	0.451
	XGBC	0.324	0.054	0.376
	MLP	0.215	0.013	0.228

### 4.2 Result analysis

In our experimental analysis, we have utilized two different CKD data sets. The first set from the study conducted by UCI CKD Risk Factor Prediction was used for training the full model and setting tuning. The Kaggle data was separated in such a way that contents of ten percent were taken as a calibration data. The remaining ninety percent of the data was to be used during the trial evaluation. We attempted a lot of ways of balancing the data such as RO, SMOTE, combinations of these with Tomek Links (TL), and a way of cutting hard examples (IHT). We ran a number of machine learning algorithms or classifiers such as Random Forest, Decision Tree, Gradient Boosting, XGBoost, Extra Trees, and Neural Network. For each setting, we measured numerous scores: Accuracy, Precision, Recall, F1-score, ROC-AUC and various measures of error like MAE, MSE, RMSE. This provided us a thorough check of how well the models work and how stable the models are when the data are unbalanced. This study also showed how fast the model memorize the data when we try them on a new set of data.

Table 4 shows that ETC achieved the highest NPV (58.33%), while MLP obtained the highest balanced accuracy (59.39%) and MCC (19%). However, specificity remained low for all classifiers, ranging from 5.74% for ETC to 25.41% for MLP. To assess uncertainty in model performance, 2,000 non-parametric bootstrap resamples of the final blind-test predictions were generated, and 95% confidence intervals were calculated for the principal performance metrics. Overall, although the models achieved high sensitivity, their limited specificity demonstrates poor discrimination of non-CKD cases.

**Table 4:** Class imbalance-aware performance evaluation For RO + TL.

Balancing Technique	Classifier	NPV(%)	Balanced Accuracy(%)	MCC(%)	95% CI Accuracy	95% Sensitivity	95% Specificity
RO + TL	DTC	17.81	56.28	12	83.73-87.28	89.68-92.67	14.08-28.85
	ETC	58.33	52.69	16	90.50-93.37	99.27-99.93	1.85-10.16
	GBC	10.87	52.22	4	80.25-84.07	86.40-89.75	9.91-23.14
	MLP	25.41	59.39	19	86.14-89.42	92.10-94.61	17.74-33.59
	RFC	21.88	55.87	13	86.58-89.83	93.35-95.73	10.77-24.03
	XGBC	16.28	54.67	9	84.25-87.77	90.68-93.49	10.74-24.03

To further assess the ability of the adapted models to correctly identify non-CKD cases, specificity was calculated from the confusion matrices obtained on the independent blind holdout set. Table 5 summarizes the specificity of the RO + TL-based models. The results indicate substantial variation across classifiers, with specificity values of 21.31%, 5.74%, 17.21%, 16.39%, 17.21%, and 25.41% for DTC, ETC, RFC, GBC, XGBC, and MLP, respectively. These results demonstrate that the models exhibit considerably lower performance in identifying non-CKD cases than in detecting CKD cases. In particular, RO + TL + ETC, despite achieving the highest overall accuracy, obtained only 5.74% specificity, highlighting the influence of class imbalance and the model’s strong tendency toward CKD classification.

**Table 5:** Specificity analysis of RO + TL data balancing technique.

Exp No	Data Balancing Technique	Classifier	Specificity (%)
1	RO + TL	DTC	21.31
2		ETC	5.74
3		RFC	17.21
4		GBC	16.39
5		XGBC	17.21
6		MLP	25.41

#### UCI CKD risk factor prediction dataset.

The UCI CKD Risk Factor dataset was used to select source training domain. The impact of some balancing methods on the optimization of models was considered. Results of the experiment indicate that hybrid balancing strategies achieved better model performance in the course of training. Random Oversampling along with Tomek linking was synonymous and achieved good results in all models. As summarized in the experimental results, the Extra Tree Classifier (ETC) combined with RO + TL achieved the most steady and competitive performance. Additionally, the model demonstrated improved ROC-AUC and reduced error metrics (MAE, MSE, and RMSE), indicating reliable discrimination capability and prediction stability. These findings reveal that RO + TL is a good method of alleviating the class imbalance in the source dataset. This method is also useful in enhancing the learning ability and strength of assembly classifiers.

#### Chronic kidney disease dataset by Rabie El Kharoua.

The target domain was the Kaggle CKD dataset on which the evaluation of generalization was conducted. This comparison had a cross-dataset target-domain adaptation learning strategy. After dividing it into 10% target adaptive calibration and 90% holdout test, we fine-tuned the pre-trained models using the same RO + TL. The combination of RO + TL + ETC provided the best results in the cross domain. It maintained a high F1 score and balanced precision and recall even with a change in data distribution; The stable ROC- AUC and smaller error values confirm that this hybrid approach works well in terms of adapting to the structure of the target dataset.

#### Reproducibility.

All experiments were implemented using Python and the Scikit-learn and imbalanced-learn libraries. The experiments were executed in a Google Colab CPU-based environment. A fixed random state of 42 was used wherever applicable to improve reproducibility. The complete experimental pipeline consisted of source-domain training, data-balancing, cross-validated hyperparameter optimization, target-domain adaptation using 10% of the target dataset, threshold selection using the target calibration subset, and final evaluation on the remaining 90% blind holdout set.

For source-domain model selection, RandomizedSearchCV was employed with five-fold cross-validation. Twenty parameter configurations were randomly sampled for each classifier and data-balancing combination, and balanced accuracy was used as the optimization criterion. The selected source-domain model was subsequently retained for target-domain adaptation. Importantly, the blind target holdout data were not used during model fitting or hyperparameter optimization. The hyperparameter search space defined for each classifier is presented in Table 6.

**Table 6:** Hyperparameter search configuration.

Model	Hyperparameter Searched
**DTC**	max_depth: None, 3, 5, 8, 10, 15, 20; min_samples_split: 2, 5, 10, 20; min_samples_leaf: 1, 2, 4, 8; criterion: gini, entropy
**ETC**	n_estimators: 100, 200, 300, 500; max_depth: None, 5, 10, 15, 20; min_samples_split: 2, 5, 10; min_samples_leaf: 1, 2, 4; max_features: sqrt, log2, None
**RFC**	n_estimators: 100, 200, 300, 500; max_depth: None, 5, 10, 15, 20; min_samples_split: 2, 5, 10; min_samples_leaf: 1, 2, 4; max_features: sqrt, log2, None
**GBC**	n_estimators: 100, 200, 300; learning_rate: 0.01, 0.05, 0.1; max_depth: 2, 3, 5; subsample: 0.8, 1.0
**XGBC**	n_estimators: 100, 200, 300; learning_rate: 0.01, 0.05, 0.1; max_depth: 3, 5, 7; subsample: 0.8, 1.0; colsample_bytree: 0.7, 1.0
**MLP**	hidden_layer_sizes: (50,), (100,), (100,50); activation: relu, tanh; alpha: 0.0001, 0.001; learning_rate_init: 0.001, 0.01

For target-domain adaptation, the target dataset was divided into a 10% adaptation subset and a 90% blind holdout subset using stratified sampling with random_state = 42. The selected source-domain classifier was cloned and retrained using the 10% target adaptation data after applying the corresponding balancing technique. The 90% blind holdout data were not used during adaptation. A decision threshold was subsequently selected using the target calibration subset according to the predefined threshold-selection criterion, and the resulting threshold was applied once to the blind holdout set for final evaluation.

For each balancing-technique/classifier combination, 20 hyperparameter configurations were randomly sampled using RandomizedSearchCV. Five-fold stratified cross-validation was employed, with balanced accuracy used as the model-selection criterion.

Hyperparameter optimization was performed separately for each of the six machine-learning classifiers using RandomizedSearchCV. For each classifier, 20 hyperparameter configurations were randomly sampled from the predefined search space and evaluated using 5-fold stratified cross-validation. The folds were shuffled, and a fixed random seed of 42 was used for reproducibility. Balanced accuracy was used as the optimization criterion because of the class imbalance in the CKD data. The best-performing configuration according to cross-validated balanced accuracy was selected and refitted using the available source-domain training data. The predefined search spaces for the six classifiers and the final selected hyperparameters for the RO + TL configuration are reported in **Table 7**.

**Table 7:** Final hyperparameter configurations corresponding to the selected balancing strategy RO + TL.

Classifier	Final Optimal hyperparameters
**DTC**	‘model__min_samples_split’: 2, ‘model__min_samples_leaf’: 1, ‘model__max_depth’: 5, ‘model__criterion’: ‘entropy
**ETC**	‘model__n_estimators’: 300, ‘model__min_samples_split’: 5, ‘model__min_samples_leaf’: 1, ‘model__max_features’: ‘sqrt,’ ‘model__max_depth’: None
**GBC**	‘model__subsample’: 0.8, ‘model__n_estimators’: 100, ‘model__max_depth’: 3, ‘model__learning_rate’: 0.05
**MLP**	‘model__learning_rate_init’: 0.001, ‘model__hidden_layer_sizes’: (100,), ‘model__alpha’: 0.001, ‘model__activation’: ‘relu’
**RFC**	‘model__n_estimators’: 100, ‘model__min_samples_split’: 10, ‘model__min_samples_leaf’: 2, ‘model__max_features’: ‘log2,’ ‘model__max_depth’: 10
**XGBC**	‘model__subsample’: 1.0, ‘model__n_estimators’: 100, ‘model__max_depth’: 5, ‘model__learning_rate’: 0.05, ‘model__colsample_bytree’: 1.0

All stochastic balancing procedures used a fixed random state of 42. For IHT, logistic regression with the LBFGS solver and a maximum of 1000 iterations was used as the hardness-estimation estimator as stated in **Table 8**.

**Table 8:** Data-balancing configuration.

Technique	Implementation
**RO**	RandomOverSampler(random_state = 42)
**SMOTE**	SMOTE(random_state = 42)
**RO + TL**	Random Oversampling → Tomek Links (sampling_strategy = “all”)
**SMOTE+TL**	SMOTE → Tomek Links (sampling_strategy = “all”)
**IHT**	InstanceHardnessThreshold using Logistic Regression (solver = “lbfgs,” max_iter = 1000)
**RO + IHT**	Random Oversampling → Instance Hardness Threshold
**SMOTE+IHT**	SMOTE → Instance Hardness Threshold

#### Cross-validation and blind holdout evaluation.

Source-domain model selection was conducted using 5-fold stratified cross-validation, with randomized hyperparameter search performed within the source-domain training data. After model selection, the target dataset was divided into a 10% target-domain adaptation/calibration subset and a 90% blind holdout subset. The adaptation subset was used for model adaptation and threshold calibration, while the blind holdout was isolated until the final evaluation. Consequently, the blind holdout did not contribute to model selection, hyperparameter tuning, adaptation, threshold selection, or preprocessing.

#### Comparative performance and significance.

The best and most consistent results in comparison with other methods were obtained with the RO + TL + ETC setup. While the work of the other classifiers (Random Forest, Decision tree, Extra trees, XGBoost, Neural Network) went well with simple balancing such as RO or SMOTE, the inclusion of RO + TL made the models more stable and error less. Again, this setup gave 91.97% accuracy, 92.24% precision, 99.64% recall and 95.8% F1-Score, improved ROC-AUC and decreased MAE, MSE and RMSE values. These numbers depict greater discrimination and robustness when the class sizes are very uneven.

In Table 9, the majority of classifiers show similarly high accuracy, largely because the holdout population is overwhelmingly CKD-positive (1372 of 1494 cases, 92%). Under RO + TL, MLP achieves 87.82% accuracy and an MSE of 12.18, comparable to the ensemble models rather than trailing them; its accuracy dips only under IHT-based techniques (e.g., 37.42% under IHT alone), where the extreme undersampling of the already-small target-domain adaptation set-reduced to roughly 24–40 training instances, leaves too little data for a neural network to fit reliably, rather than reflecting a general weakness of the architecture. Extra Trees (ETC), Random Forest (RFC), and XGBoost (XGBC) span a wider accuracy range than previously observed (86.0%–92.0%, F1 92.4%–95.8%) rather than clustering tightly together; ETC posts the highest raw accuracy (91.97%) among all six classifiers under RO + TL, though as detailed below, this reflects near-complete prediction of the majority class rather than balanced performance. The 10% target-domain adaptation set consisted of 152 CKD and 13 non-CKD cases; this extreme disproportion continues to shape classification behavior across all six models tested.

**Table 9:** Performance analysis of ML Models on Proposed Adaptation Framework using different data balancing techniques.

Exp No.	Data Balancing Technique	Model	Accuracy	Precision	Recall	F1score	ROC-AUC	MAE	MSE	RMSE
1	RO	DTC	85.5422	92.8783	91.2536	92.0588	56.2826	14.4578	14.4578	38.0235
		ETC	90.6961	92.2550	98.1050	95.0901	65.4689	9.3039	9.3039	30.5023
		GBC	82.1954	92.2137	88.0466	90.0820	62.3853	17.8046	17.8046	42.1954
		MLP	87.8179	93.3673	93.3673	93.3673	62.1678	12.1821	12.1821	34.9028
		RFC	87.9518	92.3898	94.6793	93.5205	62.6219	12.0482	12.0482	34.7105
		XGBC	85.9438	92.7206	91.9096	92.3133	61.7084	14.0562	14.0562	37.4916
2	SMOTE	DTC	77.3762	93.8136	80.6851	86.7555	60.0117	22.6238	22.6238	47.5645
		ETC	84.6051	93.0618	89.9417	91.4752	63.6288	15.3949	15.3949	39.2364
		GBC	80.5221	92.9992	85.2041	88.9312	62.4770	19.4779	19.4779	44.1338
		MLP	85.2744	93.7690	89.9417	91.8155	63.6244	14.7256	14.7256	38.3739
		RFC	83.9357	92.8139	89.4315	91.0913	63.9007	16.0643	16.0643	40.0802
		XGBC	82.0616	93.2602	86.7347	89.8792	66.4878	17.9384	17.9384	42.3538
3	RO + TL	DTC	85.5422	92.8783	91.2536	92.0588	56.2826	14.4578	14.4578	38.0235
		ETC	91.9679	92.2402	99.6356	95.7954	67.5235	8.0321	8.0321	28.3410
		GBC	82.1954	92.2137	88.0466	90.0820	62.3853	17.8046	17.8046	42.1954
		MLP	87.8179	93.3673	93.3673	93.3673	62.1678	12.1821	12.1821	34.9028
		RFC	88.2195	92.7754	94.5335	93.6462	62.7685	11.7805	11.7805	34.3227
		XGBC	86.0107	92.6007	92.1283	92.3639	63.0953	13.9893	13.9893	37.4023
4	SMOTE+TL	DTC	77.3762	93.8136	80.6851	86.7555	60.0117	22.6238	22.6238	47.5645
		ETC	84.5382	93.3181	89.5773	91.4094	63.7170	15.4618	15.4618	39.3216
		GBC	78.7149	93.3388	82.7259	87.7125	65.2538	21.2851	21.2851	46.1358
		MLP	86.9478	93.4959	92.2012	92.8440	61.9330	13.0522	13.0522	36.1278
		RFC	83.9357	92.8139	89.4315	91.0913	63.9007	16.0643	16.0643	40.0802
		XGBC	76.5730	94.3576	79.2274	86.1331	66.9882	23.4270	23.4270	48.4015
5	IHT	DTC	68.6078	91.3083	72.7405	80.9736	47.4358	31.3922	31.3922	56.0288
		ETC	32.5301	95.0495	27.9883	43.2432	65.7183	67.4699	67.4699	82.1401
		GBC	44.9799	95.2303	42.2012	58.4848	64.0049	55.0201	55.0201	74.1755
		MLP	37.4163	94.8665	33.6735	49.7041	63.4374	62.5837	62.5837	79.1098
		RFC	26.2383	97.2028	20.2624	33.5344	66.6312	73.7617	73.7617	85.8846
		XGBC	41.5663	96.8105	37.6093	54.1732	66.4000	58.4337	58.4337	76.4420
6	RO + IHT	DTC	85.5422	92.8783	91.2536	92.0588	56.2826	14.4578	14.4578	38.0235
		ETC	90.6961	92.2550	98.1050	95.0901	65.4689	9.3039	9.3039	30.5023
		GBC	76.4391	92.8571	80.5394	86.2607	60.9966	23.5609	23.5609	48.5396
		MLP	87.6841	93.4211	93.1487	93.2847	62.5329	12.3159	12.3159	35.0941
		RFC	87.7510	92.6777	94.0962	93.3816	62.8229	12.2490	12.2490	34.9986
		XGBC	85.9438	92.7206	91.9096	92.3133	61.7084	14.0562	14.0562	37.4916
7	SMOTE+IHT	DTC	77.3762	93.8136	80.6851	86.7555	60.0117	22.6238	22.6238	47.5645
		ETC	84.6051	93.0618	89.9417	91.4752	63.6294	15.3949	15.3949	39.2364
		GBC	80.5890	93.0048	85.2770	88.9734	62.5122	19.4110	19.4110	44.0579
		MLP	87.0147	93.6296	92.1283	92.8729	62.2377	12.9853	12.9853	36.0351
		RFC	83.1995	92.8845	88.4840	90.6308	64.0127	16.8005	16.8005	40.9885
		XGBC	82.0616	93.2602	86.7347	89.8792	66.4878	17.9384	17.9384	42.3538

#### The best-performing model analysis.

The highest results were recorded in the case of the RO + TL + ETC hybrid model across all experiments. Table 10 of the results of experimental evaluation gives this result. The model performed at a high level in various measurement criteria in source domain training. It showed precision and recall balanced and low prediction errors. The values of MAE, MSE and RMSE are low, which means the correct predictions in the form of probabilities and the stable work of the model. In **target-domain adaptation** evaluation, the hybrid was performing well upon calibration by the same model. The personalization performed during the adaptation was conducted on only ten percent of the target dataset. The model was also stable when there were distribution dissimilarities across datasets. This presents the advantage of the integration of balancing measures with ensemble boosting techniques. The integration enhancements across datasets on chronic kidney disease prediction.

**Table 10:** The Best Performing Model from each experiment on the dataset.

Exp No.	Data Balancing Technique	Model	Accuracy	Precision	Recall	F1-score	ROC-AUC	MAE	MSE	RMSE
1	RO	ETC	90.6961	92.2550	98.1050	95.0901	65.4689	9.3039	9.3039	30.5023
2	SMOTE	MLP	85.2744	93.7690	89.9417	91.8155	63.6244	14.726	14.726	38.3739
**3**	**RO + TL**	**ETC**	**91.9679**	**92.2402**	**99.6356**	**95.7954**	**67.5235**	**8.0321**	**8.0321**	**28.3410**
4	SMOTE+TL	MLP	86.9478	93.4959	92.2012	92.8440	61.9330	13.052	13.052	36.1278
5	IHT	DTC	68.6078	91.3083	72.7405	80.9736	47.4358	31.392	31.392	56.0288
6	RO + IHT	ETC	90.6961	92.2550	98.1050	95.0901	65.4689	9.3039	9.3039	30.5023
7	SMOTE+IHT	ETC	84.6051	93.0618	89.9417	91.4752	63.6294	15.395	15.395	39.2364

#### Statistical significance analysis.

McNemar’s test as stated in Table 11 revealed statistically significant differences in paired prediction outcomes for **12 of the 15 classifier comparisons** after Holm correction (p < 0.05p < 0.05). Significant differences were observed for all comparisons involving ETC, as well as most comparisons involving DTC, RFC, and GBC. Three comparisons were not statistically significant: DTC versus XGBC ($p_{adj}=\text{0}.\text{9}\text{1}\text{4}\text{0})$, RFC versus MLP $\left(p_{adj}=\text{0}.\text{9}\text{1}\text{4}\text{0}\right)$, and XGBC versus MLP ${(p}_{adj}=\text{0}.\text{1}\text{3}\text{1}\text{7})$. These results indicate that the classifiers generally produced statistically distinguishable prediction patterns on the blind target-domain holdout, although some classifier pairs did not differ significantly.

**Table 11:** Pairwise McNemar’s tests for the six classifiers on the 90% blind target-domain holdout.

Model A	Model B	Statistic	p-value	Holm-adjusted p-value	Significant
DTC	ETC	22.0	<0.001	<0.001	Yes
DTC	RFC	40.0	<0.001	0.00198	Yes
DTC	GBC	22.0	<0.001	<0.001	Yes
DTC	XGBC	29.0	0.4570	0.9140	No
DTC	MLP	66.0	0.0102	0.0409	Yes
ETC	RFC	14.0	<0.001	<0.001	Yes
ETC	GBC	18.0	<0.001	<0.001	Yes
ETC	XGBC	17.0	<0.001	<0.001	Yes
ETC	MLP	25.0	<0.001	<0.001	Yes
RFC	GBC	17.0	<0.001	<0.001	Yes
RFC	XGBC	33.0	0.00119	0.00593	Yes
RFC	MLP	58.0	0.6510	0.9140	No
GBC	XGBC	36.0	<0.001	<0.001	Yes
GBC	MLP	59.0	<0.001	<0.001	Yes
XGBC	MLP	70.0	0.0439	0.1317	No

#### Classification report analysis.

Table 12 presents the classification report for the RO + TL configuration across all six classifiers on the 1,494-sample blind holdout. Contrary to earlier expectations, Gradient Boosting (GBC) does **not** show the most balanced performance: it records the lowest CKD recall of the group (0.8805, corresponding to 164 missed CKD cases) and the weakest NOT-CKD F1-score (0.1307) among all six models. Extra Trees (ETC) achieves the highest overall accuracy (91.97%) and near-perfect CKD recall (0.9964), but at the cost of essentially failing to detect the minority class – NOT-CKD recall is only 0.0574 (7 of 122 correctly identified), the lowest of any classifier tested. By contrast, the Multi-Layer Perceptron (MLP) shows the most balanced profile: NOT-CKD precision, recall, and F1 are all 0.2541, and CKD precision/recall/F1 are all 0.9337 – the closest alignment between the two classes of any model evaluated, and the highest macro-averaged F1 (0.5939) in the RO + TL group. Random Forest (RFC) is a secondary candidate, with the highest CKD F1 (0.9365) alongside moderate NOT-CKD detection (recall 0.1721). These results indicate that the choice of ‘best’ classifier depends materially on whether raw accuracy or balanced class-wise performance is prioritized. This distinction is particularly important when evaluating models for potential clinical applications.

**Table 12:** Classification Report of ML Models Using RO + TL for CKD risk prediction.

Exp No.	Data Balancing Technique	Model	Class	Precision	Recall	F1-score
Proposed Experiment	RO + TL	DTC	Not CKD	0.18	0.21	0.19
			CKD	0.93	0.91	0.92
				
		**ETC**	**Not CKD**	**0.58**	**0.06**	**0.10**
			**CKD**	**0.92**	**0.99**	**0.96**
				
		GBC	Not CKD	0.11	0.16	0.13
			CKD	0.92	0.88	0.90
				
		MLP	Not CKD	0.25	0.25	0.25
			CKD	0.93	0.93	0.93
				
		RFC	Not CKD	0.22	0.17	0.19
			CKD	0.93	0.95	0.94
				
		XGBC	Not CKD	0.16	0.17	0.17
			CKD	0.93	0.92	0.92

### Confusion matrix analysis.

The confusion matrices in Fig 5 make the accuracy/balance tradeoff explicit. ETC achieves the fewest false negatives (5 missed CKD cases out of 1372) but the most false-positives on the minority class (115 of 122 NOT-CKD cases misclassified as CKD), reflecting a decision boundary that has collapsed almost entirely toward the majority class. GBC, at the other extreme, has the highest false-negative count (164), missing more true CKD cases than any other model. MLP shows the most symmetric error profile (91 false positives, 91 false negatives) and the highest true-negative count (31), indicating it is the only model among the six that meaningfully discriminates the minority class rather than defaulting to majority-class prediction. RFC (21 TN, 75 FN) and XGBC (21 TN, 108 FN) fall between these extremes. Rather than confirming RO + TL + GBC as the standout configuration, this analysis indicates that RO + TL + MLP offers the most defensible balance between sensitivity and specificity under domain transfer, while RO + TL + ETC illustrates how threshold-driven accuracy optimization can mask a near-complete failure to detect the minority class.

**Fig 5:**
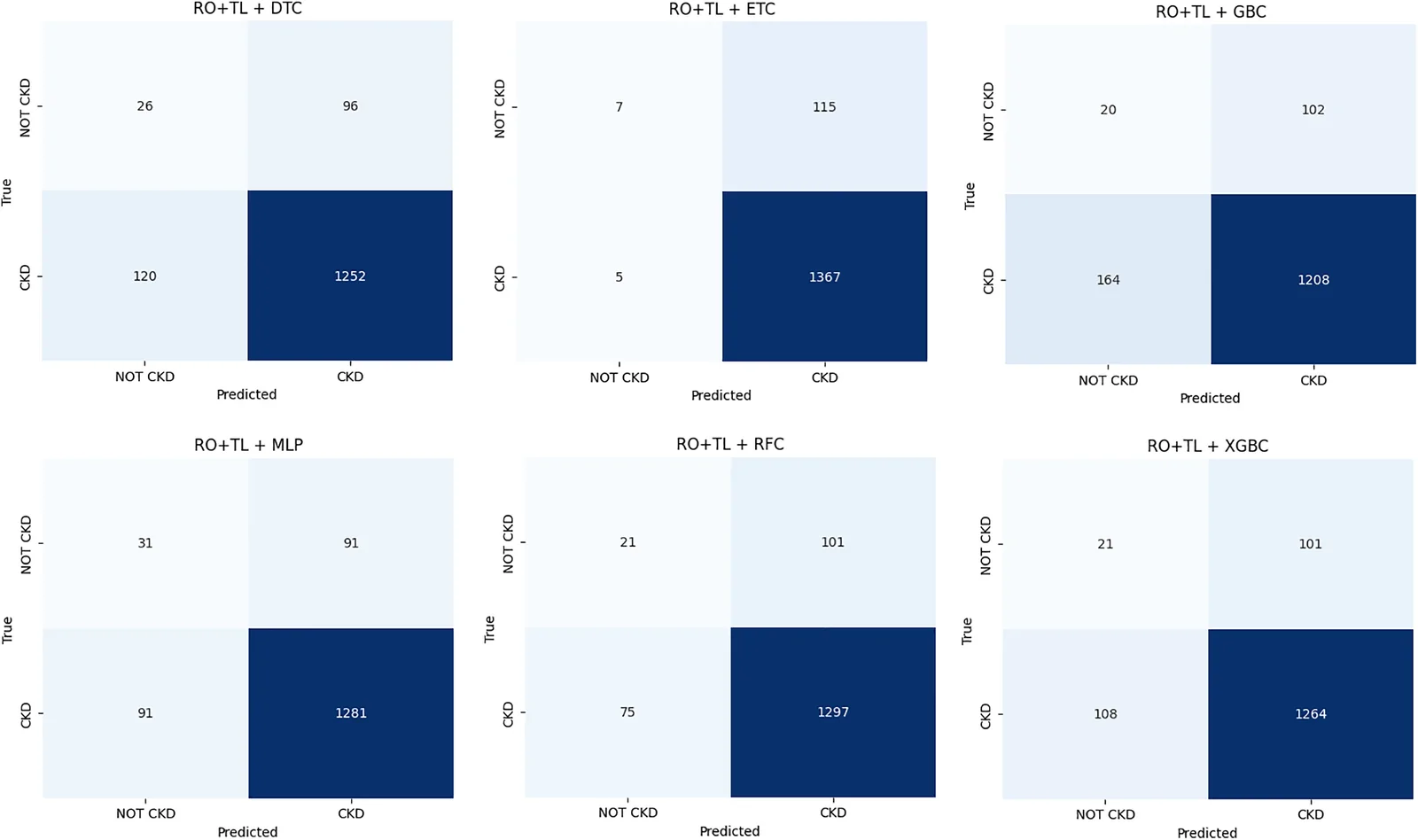
Confusion Matrix of ML Model Using RO + TL On the Dataset.

Despite accuracy in the 82%–92% range and CKD F1-scores of 90%–96%, ROC-AUC values remain modest (56.3%–67.5%). This gap arises because the underlying class ratio (1372 CKD vs. 122 NOT-CKD in the holdout) allows models to achieve high accuracy simply by favoring the majority class, while ROC-AUC – which is threshold-independent – reveals that class separability across the full probability range remains limited. The same pattern appears in the classification reports: CKD precision and recall remain consistently high (88%–100%), while NOT-CKD precision and recall are far lower and more variable across classifiers (11%–58% precision, 5.7%–25.4% recall), underscoring the difficulty of reliably predicting the minority class even after balancing. Although overall accuracy is high, the ROC-AUC values confirm that no model tested achieves consistently strong discrimination between CKD and non-CKD cases — reinforcing the need for multiple, class-aware evaluation metrics when assessing models trained on imbalanced clinical data.

The confusion matrices in Fig 5 confirm the same pattern seen in the classification reports: all six classifiers detect CKD reliably, but Not-CKD detection is consistently weak. Under RO + TL + GBC, for example, CKD precision and recall remain high (92.2% and 88.1% respectively), while Not-CKD precision and recall drop sharply (10.9% and 16.4%), with 164 CKD cases missed and only 20 of 122 Not-CKD cases correctly identified among the weakest Not-CKD detection of the six models tested. This imbalance stems from the underlying data: only 13 Not-CKD samples were available for adaptation against 152 CKD samples, leaving the models with too few genuine minority-class examples to learn reliable patterns. As a result, most classifiers bias toward predicting CKD, which raises overall accuracy while reducing the ability to correctly flag healthy cases – a pattern typical of highly skewed medical data. RO + TL + ETC shows this most starkly: near-perfect CKD recall (99.6%, only 5 missed cases) comes with just 7 of 122 Not-CKD cases correctly identified (5.7% recall), the weakest minority-class detection in the group. RO + TL + MLP is the exception, with the most balanced confusion matrix of the six (31 true negatives against 91 false positives and 91 false negatives) indicating that, unlike the other classifiers, it does not simply default to predicting the majority class. This confirms that while most classifiers achieve strong CKD detection, that strength is consistently offset by weaker performance on the Not-CKD risk factor, with the degree of imbalance varying meaningfully by model choice.

#### Key observations.

1. The best overall performance was achieved with the combination of oversampling and boundary cleaning by RO + TL + ETC. It gives 91.97% accuracy, 92.24% precision, 99.64% recall and 95.8% F1-Score. Hence, it shows that boundary cleaning and oversampling give improved generalization characteristics of the model.
2. Similar high performance was obtained by most classifiers (around 82–92%). It indicates that the properties of the dataset, mostly the prevalence of CKD samples doing a significant contribution to the predictive power.
3. Precision was very high in all the models and the recall of the models was close to 100%. This is an important point because the medical screening ability that uses the models should be attended to and not leave any CKD case out.
4. The minority Not-CKD class was still problematic. As the precision and recall scores of the class indicate low to the point of reflecting the effect of the intrinsic class imbalance.
5. The Decision Tree classifier resulted in a shortest computation time, but ETC and GBC took a more time to learn. Therefore, highlighting a strong trade-off between computation speed and predictive strength.

### 4.3. Explainable AI (XAI) analysis

In this subsection, Explainable Artificial Intelligence (XAI) analysis is presented in depth for the proposed hybrid machine learning framework. It integrates Random Oversampling (RO), Tomek Links (TL), and the Extra Trees Classifier (ETC) for Chronic Kidney Disease (CKD) prediction in a cross-dataset target-adaptive learning setting. The XAI framework provides a transparent interpretation of the model’s decision-making process. It visualizes the influence and contribution of individual clinical features to the final predictions. Interpretability techniques such as SHAP, LIME, and PIA is employed. The framework reveals both global and local feature impacts. It allows a deeper understanding of how the model differentiates between CKD and non-CKD cases. These analyses improve the interpretability of the model by providing global and local information about feature contributions. It also ensures its practical applicability in real-world clinical decision-support systems for CKD risk assessment.

#### 4.3.1. XAI analysis for the CKD dataset.

The explainability analyses were performed using the RO + TL + ETC model selected from the target-domain adaptation experiment. SHAP analysis was conducted on the 90% blind holdout dataset to examine global and class-specific feature contributions. For LIME, the 20% calibration portion of the 10% adaptation data (adaptation-calibration set) was used as the reference training/background data, while explanations were generated for observations from the blind holdout set. Permutation importance was also calculated on the blind holdout set using F1-score as the evaluation metric. The blind holdout data were not used for model training, threshold selection, or hyperparameter tuning. The resulting explanations describe model-specific feature contributions and should not be interpreted as evidence of causality, external validity, or clinical validity.

**Permutation importance analysis (PIA):** In Fig 6, permutation importance was calculated for the RO + TL + ETC model using the 90% blind holdout dataset, with F1-score as the evaluation metric. The importance value represents the change in F1-score after permutation of each feature. GFR was the most important predictor (F1 decrease: 0.0041), followed by fastingbloodsugar (0.0015) and serum potassium (0.0013). Hemoglobin and age contributed modestly (0.0007, 0.0003), while edema had no measurable effect (0.0000). Notably, diastolicbp (−0.0037) and serum creatinine (−0.0010) showed *negative* importance, meaning shuffling these features slightly improved model performance suggesting the model may be underutilizing or mis weighting them, worth flagging as a target for feature-engineering review rather than treating as confirmatory. Because PIA is model-agnostic and evaluates performance impact directly, it provides a complementary, model-agnostic assessment of feature importance.

**Fig 6:**
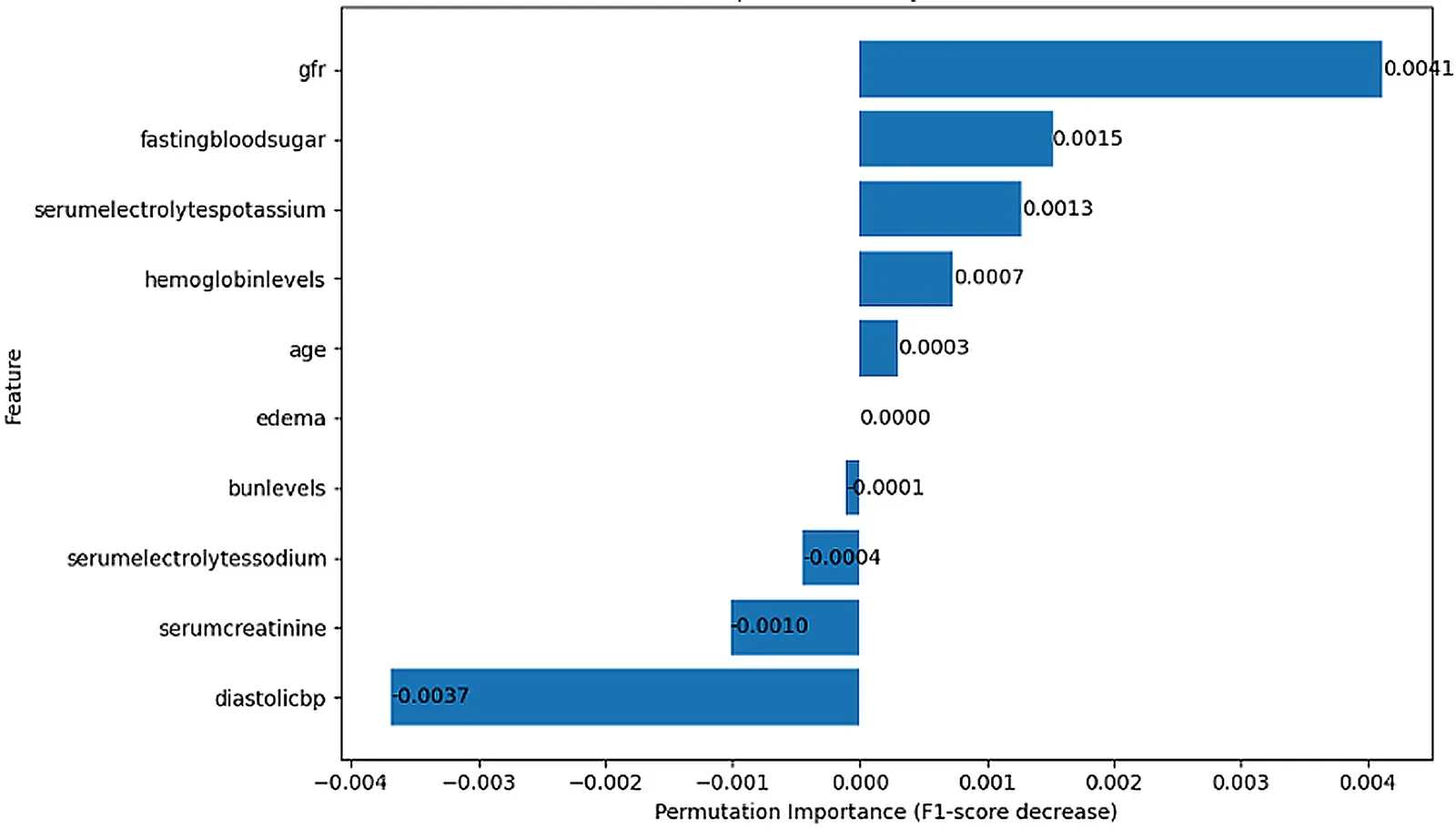
Feature Importance Using PIA.

**Shapley additive explanation (SHAP): Overall SHAP Impact –**

As shown in Fig 7, SHAP values across the 90% blind holdout samples indicate that fastingbloodsugar, diastolicbp, and GFR were among the most influential features for the RO + TL + ETC model. These results describe how the model used the available features to generate predictions and do not establish causal relationships or clinical validity. High fastingbloodsugar and abnormal GFR push predictions toward CKD; low values push toward Not-CKD. Age, potassium, sodium, creatinine, BUN, hemoglobin, and edema contribute more modestly. This is broadly consistent with PIA, though the two methods disagree diastolicbp and creatinine’s direction of influence, a discrepancy worth noting rather than glossing over, since SHAP reflects each feature’s contribution to individual predictions while PIA reflects its net effect on aggregate performance.

**Fig 7:**
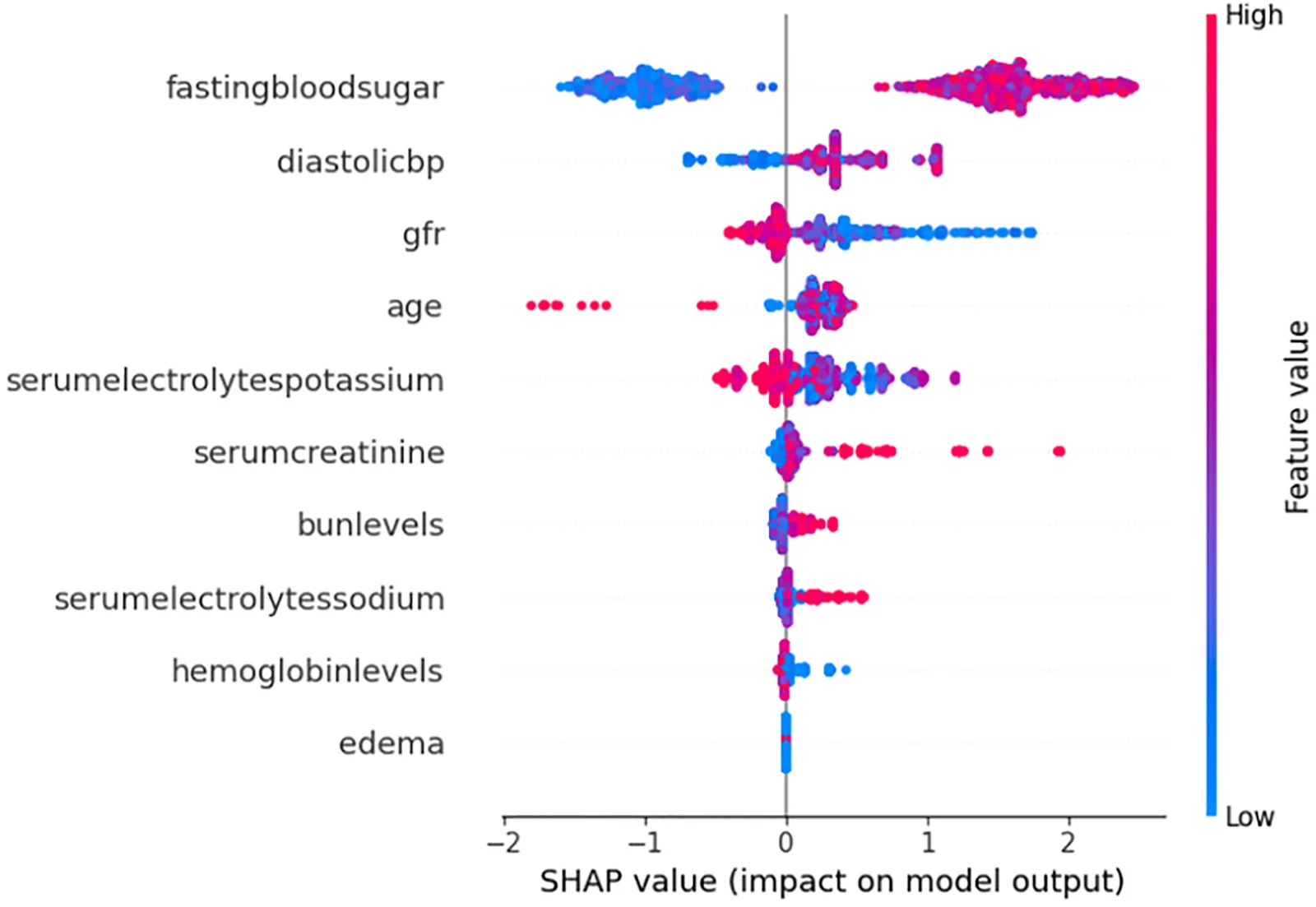
SHAP (Overall Impact) On the Dataset.

**SHAP Comparison Between CKD and not-CKD:** For CKD predictions (Fig 8A), elevated fastingbloodsugar, abnormal GFR, elevated creatinine, potassium imbalance, high BUN, and elevated diastolicbpall push the model toward a CKD prediction; older age contributes a smaller positive effect. These patterns are broadly consistent with variables commonly associated with CKD; however, this agreement with clinical knowledge does not establish causality, external validity, or clinical usefulness. The SHAP values should therefore be interpreted as model-specific feature contributions. For Not-CKD (Fig 8B), low fastingbloodsugar, normal GFR, balanced electrolytes, stable blood pressure, and normal hemoglobin and creatinine drive predictions toward the healthy class, though with visibly weaker SHAP magnitudes, consistent with the limited number of Not-CKD training examples available to the model.

**Fig 8:**
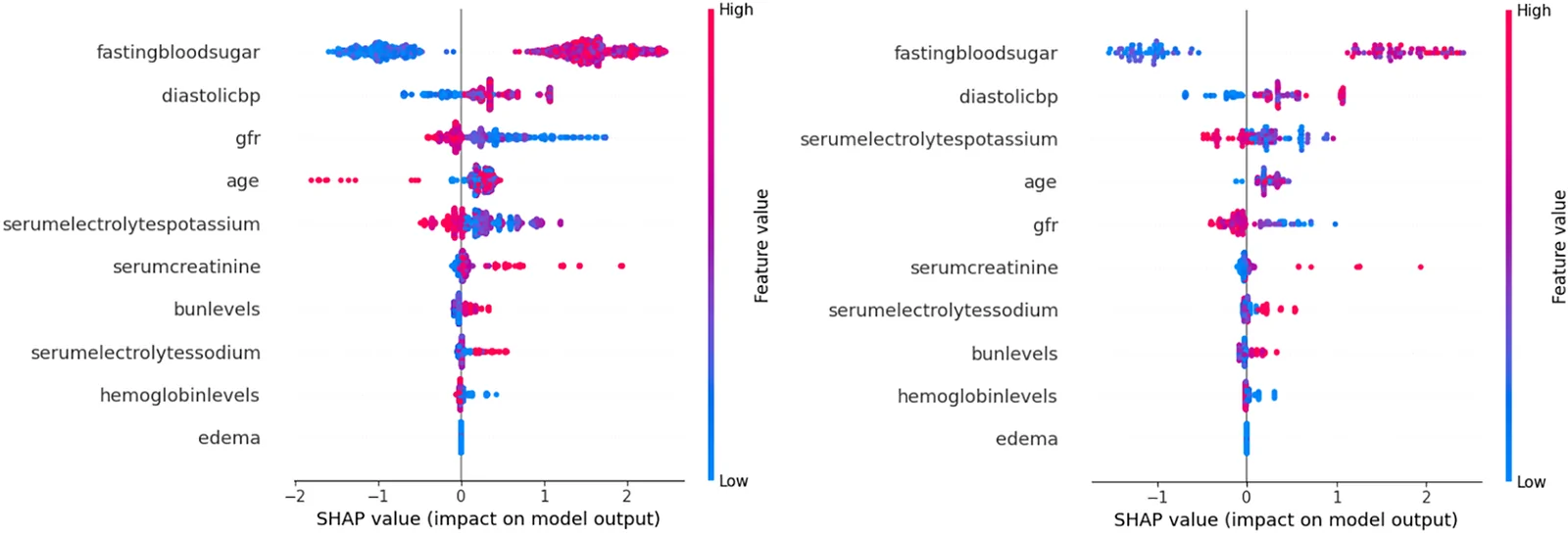
Analysis of SHAP for (a) CKD and (b) Not-CKD Class of Our Proposed Model.

Local explanation using LIME:In Fig 9, local LIME explanations are presented for observations from the 90% blind holdout dataset. The LIME explainer was constructed using the adaptation-training subset as its reference data. The explanations illustrate how individual features contributed to the model’s prediction for specific observations. These local explanations represent model behavior for individual observations and should not be interpreted as causal effects or evidence of clinical validity.

**Fig 9:**
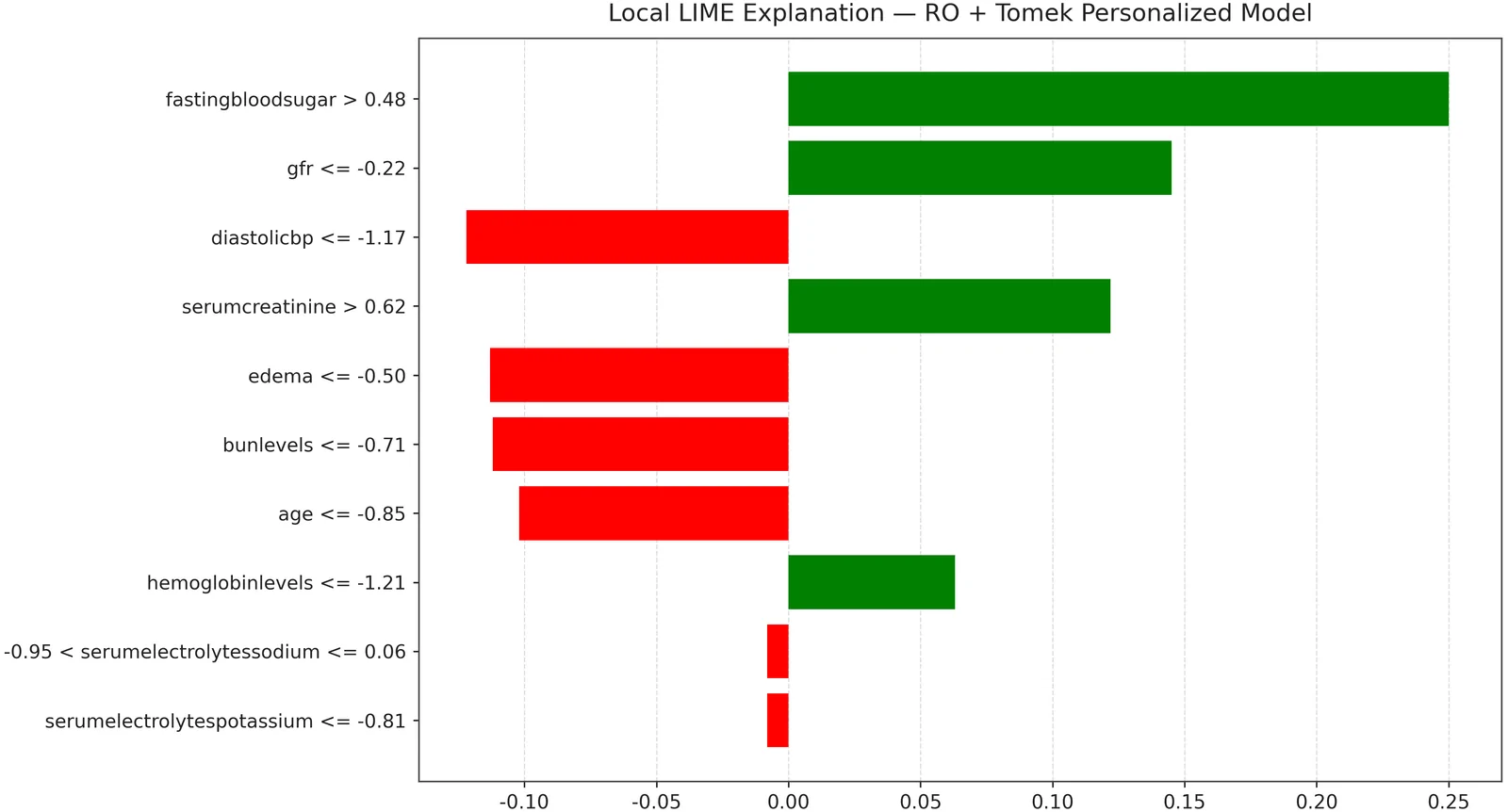
LIME Analysis For Predicting CKD Risk.

Global insights from SHAP, LIME and PIA:All three methods converge on fastingbloodsugarand GFR as the dominant predictors of CKD status, with diastolicbp and serum electrolytes as secondary contributors. Class-specific SHAP shows abnormal values consistently pushing predictions toward CKD and normal values toward Not-CKD, and LIME confirms this pattern holds at the individual-patient level. PIA independently corroborates GFR and fastingbloodsugar as the most performance-critical features, though it also surfaces a discrepancy-negative importance for diastolicbp and creatinine – that SHAP’s attribution-based view does not capture, underscoring the value of using more than one explainability method. Together, these results show the model’s predictions are grounded in clinically recognized markers of renal and metabolic function. Feature-ranking stability across independent resamples was not formally assessed; therefore, the reported rankings are interpreted as model-specific results for the evaluated holdout data rather than evidence of ranking stability.

### 4.4 Complexity analysis

Model performance was also assessed by computational cost in Table 3, measured as training time, prediction time, and total runtime for each classifier under RO + TL balancing. This test evaluates the practical feasibility of deploying the hybrid framework on real-world healthcare data, where computational cost matters alongside predictive accuracy. All six classifiers: DTC, ETC, GBC, MLP, RFC, and XGBC were evaluated under identical experimental conditions, with results reported in Table 3. Decision Tree (DTC) was by far the most computationally efficient model, completing training and prediction in 0.005s total. Extra Trees (ETC) was the most expensive overall (0.542s), with its cost driven primarily by prediction time (0.097s) reflecting the overhead of querying its full ensemble at inference. Gradient Boosting (GBC) incurred the second-highest total cost (0.451s), driven almost entirely by training time (0.449s), with negligible prediction overhead (0.002s)—a favorable profile for deployment scenarios where inference speed matters more than training speed. XGBoost (0.378s) and MLP (0.228s) fell in the middle, while Random Forest (0.181s) was comparatively efficient despite its ensemble voting process. These results show that RO + TL balancing does not impose meaningful additional computational overhead, and that the most computationally efficient model (DTC) is not the strongest predictor. This confirms a genuine trade-off between computational efficiency and predictive strength: models offering the best minority-class detection (MLP, RFC) also carry higher computational cost, while the fastest model (DTC) sacrifices classification balance for speed. This trade-off is a practical consideration for deployment in cross-dataset, target adaptive chronic kidney disease risk-prediction systems, where the choice of classifier should reflect the relative priority placed on inference speed versus diagnostic reliability.

## 5. Discussion

The paper introduces the new machine learning model, which will be used in the Chronic Kidney Disease detection. The model also clarifies how it predicts things to help the physicians. We experimented the model using two separate datasets. The initial dataset is UCI CKD Risk Factor Prediction Dataset. It was gathered at Enam medical college in Bangladesh. The second data is the Chronic Kidney Disease Dataset. Rabie El Kharoua published it to Kaggle. We observed how a model that was trained with one dataset may work well on another dataset. The imbalance of datasets was very high. Thus, a few data balancing methods were used. These techniques were Random Oversampling, SMOTE, Tomek Links and Instance Hardness Threshold. We also did combinations of these methods. Our machine learning models had a number of trained ones. These were Decision Tree, Random Forest, Extra Trees, Gradient Boosting and XGBoost. Simple neural network model was also tested. Explainability techniques were utilized to decode predictions. These were SHapley Additive explanations, Local Interpretable Model-agnostic Explanations and Permutation Importance. These are the means which facilitated the clarification of the predictions. The performance of Extra Tree was the best. Random Oversampling and Tomek Links achieved the best results. Together this enhanced balancing of the models/accuracy. It also gave more explanations to the doctors.

A few models and data balancing techniques were tested. The system was reliable in various combinations. Extra Trees, Random Forest, Decision Tree, Gradient Boosting, XGBoost and Multilayer Perceptron were tested. We also experimented with the balancing techniques with such models. Random Oversampling and Tomek Links gave the highest result. Extra Tree Classifier was applied to this combination. The model had a capacity of 91.97% accuracy, 92.24% precision, 99.64% recall and 95.8% F1-Score. Although RO + TL + ETC achieved the highest accuracy and CKD recall, its ROC-AUC of 67.52% indicates limited discriminatory ability across probability thresholds. This result, together with the low specificity of 5.74%, indicates that the high accuracy should be interpreted cautiously because of the strong class imbalance in the blind target holdout. The results indicate different performance trade-offs among the evaluated classifiers rather than a universally superior model. ETC achieved the highest accuracy, sensitivity, and F1-score, whereas MLP achieved the highest balanced accuracy, specificity, and MCC. Nevertheless, the low specificity across all classifiers indicates limited discrimination of non-CKD cases. Therefore, differences in individual point estimates should not be interpreted as evidence of overall model superiority. The bootstrap confidence intervals and paired statistical comparisons provide a more cautious assessment of these differences. Explainable AI methods were also used by us. They consisted of SHapley Additive exPlanations, Local Interpretable Model-agnostic Explanation and Permutation Importance. These techniques determine the clinical variables of predictions. The low specificity across all models (0.91–27.27%) indicates that the current framework is unsuitable for clinical screening. Substantially more non-CKD samples are required before deployment can be considered. Therefore, although the framework demonstrates high sensitivity for CKD cases, its limited ability to correctly identify non-CKD cases prevents it from being considered suitable for standalone clinical decision-making at this stage. The system can assist in the identification of the Chronic Kidney Disease by acting on the target-adapting learning strategy.

The HXAI-ML model was tested to generate results on a dataset on chronic kidney disease (CKD). It received 91.97% accuracy, 92.24% precision, 99.64% recall and 95.8% F1-Score with the RO + TL + ETC setting. The findings indicate that the hybrid approach can efficiently cope with the huge imbalance in classes. It also provides reliable forecasts on data at other locations. The model with RO + TL was most successful. It was more superior than the Random Forest, Extra Trees, XGBoost and Multilayer Perceptron. It possessed better ROC -AUC and fewer errors. The errors were MAE, MSE and RMSE. The two balancing techniques have resulted in the good performance. Random Oversampling will increase the examples of the minority group. Tomek Links removes redundant and overlapping instances. The two assist the model to understand better boundaries of classes. They ensure that the distinction between the CKD and non-CKD patients is clearer. They also perform predictions that are more consistent on other datasets. The model results are also supported by explainability tools. They include SHapley Additive exPlanations (SHAP), Local Interpretable Model-agnostic Explanations (LIME) and Permutation Importance. The tools demonstrate the general and the actual functioning of the model where each prediction is concerned [51]. This assists physicians to observe the critical CKD risk factors. It was determined that some major clinical features were identified: the Glomerular Filtration Rate, the level of fasting sugar in the blood, electrolytes, and hemoglobin. These are forces with a great influence on the CKD predictions. High accuracy and balance of data indicate the strength of HXAI-ML. Predictions too are clearly explained in the model. That is why it is applicable in identifying the risks of CKD. The cross-dataset target-adaptive model enhances the flexibility to new data. Initially it is trained on a huge base dataset. After that it is fined-tuned on a small target set. This allows the model to adapt to changes in clinical information distributions. These findings [52] It may also be useful in the future clinical decision support systems.

### 5.1. Validation of research question

#### RQ1.

The research experimented with the methods of balancing the data, such as Random Oversampling (RO), SMOTE, RO + Tomek Links (RO + TL), SMOTE + Tomek Links (SMOTE+TL), Instance Hardness Threshold (IHT), RO + IHT, and SMOTE + IHT. They have also applied some machine learning classifiers. RO + TL has produced the best result, as it was able to address the imbalance of the CKD data set. Oversampling and cleaning of the boundaries increased the clarity of the classes and eliminated noise of the samples at the decision boundary. Among the RO + TL models, ETC achieved the highest accuracy (91.97%), sensitivity (99.64%), and F1-score (95.80%), while MLP achieved the highest balanced accuracy (59.39%), specificity (25.41%), and MCC (0.19). These results indicate different performance trade-offs rather than a universally superior model.

#### RQ2.

The explanatory AI (XAI) tools were added to the study to learn why the model made its predictions. They applied SHAP, LIME and Permutation Importance Analysis (PIA) to provide the explanations of the predictions in general and patient-wise. The key predictors of CKD identified by SHAP included glomerular filtration rate (GFR), electrolytes, and hemoglobin; and fastingbloodsugar. LIME described the individual cases by demonstrating the combination of these factors to influence the result of one patient. PIA confirmed these factors by examining the performance of the model in the case that each factor was shuffled. All these methods combined resulted in clear decisions of the model that were in agreement with previously known CKD risk factors.

#### RQ3.

The framework combined data balancing, ensemble/neural classifiers, and two-stage cross-dataset adaptation: models were trained on the UCI CKD dataset, evaluated zero-shot on Kaggle CKD, then adapted using only 10% of Kaggle data, with the remaining 90% held out for blind testing. Results show adaptive generalization is achievable but trades off accuracy against minority-class sensitivity. Under RO + TL, most classifiers reached 82–92% accuracy with strong CKD recall (up to 99.6%), confirming effective majority-class transfer under domain shift. Not-CKD recall remained weak across all models (5.7%–25.4%), reflecting the scarcity of minority-class examples (13 vs. 152) rather than a failure of the adaptation mechanism itself. MLP gave the most balanced performance across both classes, while accuracy-optimized models like ETC scored high largely by defaulting to the majority class. This confirms that target-domain adaptation with minimal labeled data is viable for cross-dataset CKD prediction, but that class-balance-aware evaluation-not accuracy alone is essential to judging its clinical reliability.

The paper suggests the hybrid CKD predictive model based on data balancing, and ensemble learning. Explainable AI approaches are also used to provide better insights into predictions in the model. This methodology deals with disparities of classes and poor interpretability of medical datasets. Extra Tree Classifier is able to differentiate between CKD and cases that are not. The RO and Tomek Links balancing strategy enhances performance and reliability of the model. SHAP, LIME, and Permutation Importance Analysis are some of the explainable AI methods. These approaches determine significant variables that affect the prediction of CKD. They are also more transparent and informative of model choices. The findings indicate that the approach is valid and edifying. It will be able to aid the early kidney disease detection and aid clinicians in their clinical decision-making.

### 5.2. Methodological and potential clinical implications

The results have methodological implications for CKD prediction under severe class imbalance and cross-dataset distribution differences. The target-domain adaptation experiment demonstrates that a model trained on a source dataset can be recalibrated using a limited portion of target-domain data before evaluation on a blind holdout. However, the present results do not establish clinical usefulness. In particular, the low specificity observed across the adapted models indicates that many non-CKD cases are incorrectly classified as CKD. Therefore, the framework should not currently be used for clinical screening, diagnosis, or clinical decision support. The XAI analyses provide information about model feature contributions, but interpretability alone does not establish clinical validity or reliability. Larger and clinically representative datasets, including substantially more non-CKD cases and independent external validation, are required before clinical utility can be evaluated.

Additionally, explainable AI applications used for the study including SHAP, LIME, and PIA. Such tools will assist in demonstrating the patient facts that are key in the prediction of CKD. That simplifies the system and provides physicians with assurance that the system is operating properly. The other characteristic of the work is a cross-dataset target-adaptation learning framework. The model is trained on a single data set, and then it is fitted with a different dataset. This demonstrates that the approach is applicable to a broad variety of types of health data. The model is able to expand and surpass most clinics and hospitals. Altogether, the methodology allows physicians to make the decision according to the data that is clear and understandable. It assists in risk detection of CKD earlier and in creating superior relationships of treatment in routine healthcare practice.

### 5.3. Novelty of the proposed HXAI-ML framework

The new HXAI-ML system predicts chronic kidney disease. It applies data balancing and a number of machine learning models. It is capable of learning some new examples. It also explains why it made every prediction. Most of the medical data contain not-CKD cases rather than CKD. There are also different data presented across the studies. Such tools require working doctors to be given directions. These are some of the common problems of data addressed by this study. Everything is combined in a single workflow in the new framework. The novelty of the proposed HXAI-ML framework lies in integrating class-balancing strategies, source-domain model optimization, limited-sample target-domain adaptation, and explainable AI within a cross-dataset CKD evaluation. The framework provides an experimental setting for examining how models trained on one CKD dataset behave after adaptation to a different target dataset. The results also demonstrate the importance of evaluating class-specific performance rather than relying on overall accuracy alone.

#### Hybrid data balancing approach.

The major point is the hybrid between Random Oversampling (RO) and Tomek Links (TL). Random Oversampling makes more examples of the minority group. The case of chronic kidney disease constitutes a minority group. Tomek Links eliminates redundant or similar examples towards the decision boundary. Combining them enhances the ability of models to differentiate between CKD and non-CKD. They also reduce the confusion in the prediction of the two groups. The RO + TL method was evaluated across the six classifiers and showed different performance trade-offs. Although RO + TL combined with ETC produced the highest accuracy and sensitivity, RO + TL combined with MLP achieved the highest balanced accuracy and specificity. Therefore, RO + TL cannot be considered universally superior across all classifiers or performance measures. The mixture eliminates the powerful disparity of classes in CKD datasets. It also enhances the modeling performance both on older and new data.

#### Explainable AI techniques integration.

Black-box models are used in most studies, which are difficult to comprehend. Our HXAI-ML model includes three explainability instruments, namely SHAP, LIME, and Permutation Importance Analysis (PIA). SHAP demonstrates the total impact of all the clinical factors on the predictions of every patient. Using a simple model to simulate the complex model, LIME is able to explain the result of a single patient. The Permutation Importance Analysis is used to examine the significance of every feature. We modify the values of a feature and observe the variation in the performance of the model. This approach provides a general understanding of the way the model functions. It also aids in the reason why the model predicted certain things concerning patients. These descriptions assist physicians to visualize the risk factors of chronic kidney disease that are the most crucial. They also make doctors become even more trustful to the prediction model.

#### Cross dataset Target-Domain Adaptation model.

A further contribution is a cross-dataset target-domain adaptation framework: the model is trained on the UCI CKD dataset, then adapted to the Kaggle CKD dataset using only 10% of the new data, with the remaining 90% held out for blind testing. This experimental design provides a basis for investigating adaptation across datasets with differing patient populations or data collection practices; however, deployment across clinical institutions would require independent clinical validation. Performance under adaptation was high for the CKD class, but minority-class performance remained limited More broadly, the HXAI-ML framework combines data balancing, ensemble and neural classifiers, explainable AI (SHAP, LIME, permutation importance), and cross-dataset adaptation to support interpretable, adaptable CKD prediction across differing data sources while class imbalance and domain shift remain challenges to address in future work.

#### Class imbalance.

Data in the CKD used in this research has an extreme imbalance between CKD and non-CKD cases. The given RO + TL hybrid balancing strategy is an efficient solution to this problem since it entails the combination of Random Oversampling and Tomek Links. Random Oversampling increases the number of examples belonging to the minority class which is the case of chronic kidney disease. Tomek Links eliminates samples that lie close to or across the boundaries of the decision. A combination of these steps enhances the accuracy of the model. It also classifies cases of CKD in a better manner and maintain the balance of the predictions on both sides.

#### Interpretability.

The framework employs the Explainable AI tools such as SHAP, LIME and Permutation Importance. The techniques assist the user in knowing how the models make predictions. They also make more people trust the results of the model. These tools give a good understanding of the effects of clinical variables on the prediction of CKD. Consequently, this allows the clinicians to learn how the model makes the decision, which leads to more trust and allows informed clinical interpretation.

#### Scalability and adaptability.

The framework allows enhancing work with various datasets due to a cross-dataset **target-domain adaptation** process. The model is firstly trained on the UCI CKD Risk Factor Prediction. After that, it is properly adapted with the Kaggle CKD data through a small calibration subset. The cross-dataset target-domain adaptation experiment demonstrates methodological feasibility for adapting a source-trained model to a different target dataset using a limited calibration subset. However, evaluation was performed using only two public datasets, and no third independent dataset was used for external validation. Therefore, the present results do not establish broad generalizability or clinical deployability.

#### Outsourcing and comprehensive experimental validation.

Two CKD datasets were used to validate the proposed framework. UCI dataset was used as the source rule and the Kaggle dataset was used as the target rule, a target-domain adaptation and a blind test. Several machine learning models and methods of balancing were tested in terms of various performance measures such as accuracy, precision, recall, F1-score, ROC-AUC, MAE, MSE, and RMSE. The experimental findings showed different performance trade-offs across the RO + TL classifier combinations. RO + TL + ETC achieved the highest accuracy, sensitivity, and F1-score, whereas RO + TL + MLP achieved the highest balanced accuracy, specificity, and MCC. These results do not establish a universally superior classifier, particularly given the low specificity observed across all models. The model recorded about 91.97% accuracy, 92.24% precision, 99.64% recall, which reflect the great predictive potential and strength to identify the cases of CKD. It was done evenly because of transferring the conditions to a different domain.

#### Interpretability and potential clinical relevance.

The proposed HXAI-ML framework is unlike most machine learning models that focus on predictive accuracy only, instead of on performance and interpretability. The ensemble learning methodology determines significant clinical variables to influence CKD prediction. Such factors are the rate of glomerular filtration, fastingbloodsugar, electrolytes, and hemoglobin. The descriptions allow clinicians to get insight into the influence of each characteristic on the outcomes of prediction. These explanations provide potentially useful information for understanding model behavior, but they do not establish clinical validity or suitability for clinical decision-making. Given the low specificity observed in the target-domain holdout, the current framework should not be considered suitable for standalone clinical screening or clinical decision support. This interpretability is vital in applications in healthcare where judgment and reliability are very vital in the care of a patient. Contribution to the Field The offered HXAI-ML model has a variety of implications to the investigation of CKD prediction:

- Focusing on the challenge of finding a balance of prediction accuracy and interpretability with the help of ensemble learning and explainable AI.
- The implementation of successful hybrid balancing method (RO + TL). That supports evaluation of model behavior under cross-dataset distribution differences and lessens inequity of classes within CKD samples.
- Suggesting a **target adaptive learning** model using cross-dataset. the study evaluates whether source-trained models can be adapted to a different target dataset using a limited calibration subset.

The absence of interpretable structures would make the timely detection of CKD and give straight forward clinical instructions hard. The HXAI-ML system is a compromise to strike a balance between the accuracy of the predictions and their interpretability and cross-dataset adaptability. It may be used when it comes to a viable machine learning tool in CKD risk forecasting.

### 5.4 Limitations of the proposed work

Despite the methodological findings of the proposed HXAI-ML framework of the proposed HXAI-ML framework, several limitations should be acknowledged. First, the original target dataset contained a highly imbalanced class distribution, with only 13 non-CKD cases and 152 CKD cases in the 10% adaptation subset. In the final 90% blind holdout, there were 122 non-CKD and 1,372 CKD observations. This severe imbalance limits the reliability of specificity and other non-CKD performance measures, resulting in severe class imbalance and limiting the reliability of specificity and other non-CKD performance measures. The low specificity observed across the models also indicates a high false-positive rate; therefore, the proposed framework should not be considered suitable for standalone clinical screening or deployment at this stage. Second, the adapted models exhibited low specificity for the non-CKD class. For the selected RO + TL + ETC model, specificity was only 5.74%, indicating a false-positive rate of 94.26% among non-CKD cases in the blind holdout set. More broadly, the low specificity observed across the evaluated models indicates that the current framework is not suitable for standalone clinical screening or clinical deployment. Substantially larger and more representative non-CKD samples are required to establish whether the observed performance generalizes to clinically realistic populations. In addition, no third independent dataset was used for external validation, so the generalizability of the findings to other populations and clinical settings remains uncertain. The study uses only 10% of the target-domain data for adaptation, with the remaining 90% reserved for blind evaluation. Although this design demonstrates the feasibility of limited-sample target-domain adaptation, the small adaptation sample further restricts the amount of information available for learning the target-domain distribution. Finally, the study is limited to structured clinical data and does not compare the proposed approach with deep learning or transformer-based models. Future research should evaluate larger and more representative datasets, particularly with substantially more non-CKD cases, and include independent external validation before clinical applicability can be considered.

### 5.5 Future work

Future research should focus on addressing the severe underrepresentation of the non-CKD class and improving the generalizability of the proposed framework. Generative Adversarial Networks (GANs) could be investigated to generate synthetic non-CKD samples and reduce class imbalance. Cost-sensitive learning should also be evaluated as an alternative to oversampling, allowing greater penalties to be assigned to misclassification of the minority class without artificially generating observations. Furthermore, the framework should be validated on a third independent dataset, such as the NHANES CKD dataset, to assess its robustness across different populations and data distributions. Finally, future studies should evaluate whether these approaches can improve specificity while maintaining the high CKD detection sensitivity observed in the current study.

## 6. Conclusions

This study presents a machine-learning framework for chronic kidney disease (CKD) prediction that addresses three key challenges: class imbalance, cross-dataset target-domain adaptation, and model interpretability. Several data-balancing strategies, including Random Oversampling, SMOTE, Tomek Links, and Instance Hardness Threshold, were evaluated with six classifiers: Decision Tree, Random Forest, Extra Trees, Gradient Boosting, Extreme Gradient Boosting, and Multi-Layer Perceptron. The framework was evaluated using a source CKD dataset and a separate target dataset, with 10% of the target data used for limited-sample domain adaptation and the remaining 90% retained as a blind holdout set. Among the evaluated configurations, RO + TL + ETC achieved the strongest overall performance, obtaining 91.97% accuracy, 92.24% precision, 99.64% recall, and 95.80% F1-score, with an ROC-AUC of 67.52%. SHAP, LIME, and permutation importance were further applied to improve the interpretability of the adapted models and identify influential clinical features. Despite the high overall classification performance, the low specificity of RO + TL + ETC (5.74%) demonstrates a substantial limitation in correctly identifying non-CKD cases. Therefore, the proposed framework should not be considered clinically deployable as a CKD screening system at its current stage. The findings demonstrate the methodological feasibility of evaluating cross-dataset target-domain and explainable machine learning under limited target-domain data, while highlighting the need for improved class-wise discrimination. Future research should focus on increasing the representation of non-CKD cases, improving probability calibration and threshold selection, investigating cost-sensitive learning and synthetic-data generation, and conducting external validation on an independent and clinically representative dataset. These steps are necessary before the framework’s clinical utility can be reliably assessed.
