# Supplementary material for: An explainable machine learning framework for cross-dataset chronic kidney disease risk prediction using hybrid data balancing
Source: PLoS One. 2026 Nov 5;21(11):e0362320. doi: 10.1371/journal.pone.0362320 (PMC13739112; doi:10.1371/journal.pone.0362320)
Supplement: Supplementary file 1 [file pone.0362320.s002.docx]

**RESPONSE TO REVIEWERS**

| **Manuscript Title** | An Explainable Machine Learning Framework for Cross-Dataset Chronic Kidney Disease Risk Prediction Using Hybrid Data Balancing |
| --- | --- |
| **Journal** | Plos One |

We sincerely thank the Editor and reviewer 1 for their thorough and constructive assessment of our manuscript. Their feedback has strengthened the positioning, methodological transparency, and presentation of the revised work.

Below is a detailed point-by-point response to each comment, organized by source.

# Response to Additional Editor

| **#** | **Reviewer Comment** | **Authors' Response and Location in Revised Manuscript** |
| --- | --- | --- |
| 1 | Thank you for submitting your manuscript to PLOS ONE. Your study tackles an important clinical issue and explores class-balancing, cross-dataset model adaptation, and explainable AI for predicting chronic kidney disease risk. Using public datasets and several interpretability methods could make a valuable contribution. However, after reviewing your manuscript and the detailed reviewer comments, I have decided that the paper does not yet meet the journal’s standards for technical quality, analytical rigor, reproducibility, clear reporting, and proper interpretation of results. I am inviting you to submit a thoroughly revised version. Please note that this is a request for major methodological and analytical changes, not just editorial corrections. | **We sincerely thank the Editor for the careful assessment of our manuscript and for the opportunity to submit a substantially revised version.**  We appreciate the concerns regarding technical quality, analytical rigor, reproducibility, reporting, and interpretation. In response, we have conducted a comprehensive re-analysis of the study using a revised and carefully controlled target-domain adaptation pipeline. In particular, the experimental results have been regenerated using the 10% target-domain adaptation setting, with the remaining data retained as a blind holdout evaluation set. The revised analysis identifies RO+TL+ETC (Random Oversampling + Tomek Links + Extra Trees) as the best-performing configuration under the revised evaluation procedure, rather than the previously reported RO+TL+GBC configuration.  We have also made the following substantive methodological and reporting changes:   1. **Revised the performance analysis** to ensure that the reported accuracy, recall, F1-score, specificity, ROC-AUC, and confusion matrices are mathematically consistent. 2. **Removed unsupported 100% recall and few-shot-learning claims** and replaced them with the actual results and the more appropriate term **“limited-sample domain adaptation.”** 3. **Reported specificity explicitly** and acknowledged the substantial difficulty of identifying non-CKD cases. 4. **Revised the clinical interpretation** to make clear that the current models should not be considered clinically deployable screening systems because of their low specificity. 5. **Added statistical significance analysis**, including pairwise McNemar tests with Holm adjustment, to assess whether differences between classifiers are statistically significant. 6. **Expanded the methodological description of hyperparameter tuning**, including the search strategy, parameter ranges, selected parameters, and random seeds. 7. **Added computational-environment information**, including hardware, operating system, Python version, and relevant software/library versions, to improve reproducibility. 8. **Clarified the preprocessing and adaptation procedure**, including the distinction between preprocessing performed before adaptation and the subsequent target-domain adaptation process. 9. **Corrected the XGBoost objective-function notation** and clarified the definitions of all mathematical terms. 10. **Revised the SHAP analysis and feature labels** to ensure consistent terminology between the figures and manuscript. 11. **Expanded the limitations section** to explicitly acknowledge the very small number of non-CKD cases, low specificity, absence of independent external validation, and other methodological limitations. 12. **Revised the discussion and conclusion** so that they accurately reflect the observed results and do not overstate clinical applicability. 13. **Expanded the future-work section** with specific directions, including synthetic generation of minority-class cases, cost-sensitive learning, and validation using an independent dataset such as NHANES. 14. **Corrected and audited the references**, including the Almasoud et al. citation, Rezk et al. year designations, and addition of the foundational SMOTE reference (Chawla et al., 2002).   These changes represent a substantial methodological and analytical revision rather than solely editorial modification. We believe that the revised manuscript provides a more transparent, reproducible, and appropriately cautious assessment of cross-dataset CKD prediction under limited target-domain adaptation. |
| 2 | The main concern is the inconsistency between the performance results shown in the abstract, tables, main text, and the confusion matrices in Figure 5. The manuscript states that the Random Oversampling with Tomek Links and Gradient Boosting setup achieved 91.83% accuracy and 100% recall. But the confusion matrix shows about 84.61% accuracy and 89.88% recall. These differences are significant because they affect your main claim about the best-performing model. The manuscript often highlights the RO+TL+GBC setup as the top performer, but the confusion matrix suggests another model may do better on some metrics. | **Thank you for the valuable concern.**  We thank the reviewer for identifying this inconsistency. We agree that the previously reported values for the Gradient Boosting Classifier (GBC) were not consistent with the confusion matrix, indicating an error in the earlier result-generation pipeline.  To address this issue, we reran the complete 10% target-domain adaptation experiment using the corrected and leakage-controlled pipeline. In the revised experiment, the target dataset is divided into **10% adaptation/calibration data and 90% completely blind holdout data**, with threshold selection performed using the adaptation/calibration set only. The final performance metrics and confusion matrices are now generated from the **same holdout predictions**, ensuring mathematical consistency among Tables 3, 5, and Fig. 5.  Under the corrected pipeline, the **RO+TL+ETC** configuration is identified as the best-performing model based on the reported accuracy criterion. On the 1,494-sample blind target holdout, RO+TL+ETC achieved **91.97% accuracy, 92.24% precision, 99.64% recall, 95.80% F1-score, and 67.52% ROC-AUC**. Its corresponding confusion matrix contains **TN = 7, FP = 115, FN = 5, and TP = 1,367**, which gives:  $Accuracy=\frac{TN+TP}{TN+FP+FN+TP}=\frac{7+1367}{7+115+5+1367}=0.9197$  $Recall=\frac{TP}{TP+FN}=\frac{1367}{1367+5}=0.9964$  Thus, the revised tables and figure are internally consistent. For GBC, the corrected RO+TL+GBC result is **82.20% accuracy and 88.05% CKD recall**, with the corresponding confusion matrix derived from the same holdout predictions. Therefore, the previous statement identifying GBC as the best model has also been removed/revised. The revised analysis now distinguishes between **highest raw accuracy (RO+TL+ETC)** and **more balanced minority-class behavior (RO+TL+MLP),** rather than treating GBC as the overall champion. |
| 3 | You will need to rerun and independently verify the entire analysis. All numbers in the abstract, main text, tables, figures, supplementary materials, and conclusion must come from the same final analysis. The revised manuscript should clearly explain how you calculated accuracy, sensitivity, specificity, precision, recall, F1-score, ROC-AUC, and any other performance measures. Make sure the positive and negative classes are defined the same way throughout. In your response letter, include a clear record showing how you generated the corrected values for each table and figure. | **Thank you for this important comment.**  We agree that all reported results must originate from one consistent final analysis. We therefore reran and independently checked the complete experimental analysis using the finalized 10% target-domain adaptation pipeline. All numerical results reported in the revised manuscript, including the abstract, main text, tables, figures, supplementary materials, and conclusion, were regenerated from this final analysis.  In the revised analysis, the data and class definitions were kept consistent throughout: CKD was defined as the positive class (1), while non-CKD was defined as the negative class (0). The 10% target-domain adaptation data were used for model adaptation/calibration, while the designated blind holdout set was used for final performance evaluation. Thresholds were selected using the specified calibration procedure and were not optimized using the blind holdout labels.  The performance measures were calculated as follows:   - **Accuracy** = (TP + TN) / (TP + TN + FP + FN) - **Precision (PPV)** = TP / (TP + FP) - **Recall/Sensitivity** = TP / (TP + FN) - **Specificity** = TN / (TN + FP) - **F1-score** = 2 × (Precision × Recall) / (Precision + Recall) - **Balanced accuracy** = (Sensitivity + Specificity) / 2 - **ROC-AUC** = area under the ROC curve calculated from the continuous predicted probabilities rather than thresholded predictions. - **MAE, MSE, and RMSE** were calculated from the binary observed and predicted class labels according to the definitions described in the revised methodology.   We also independently checked the reported values against the corresponding confusion matrices. In particular, TP, TN, FP, and FN were extracted directly from the final predictions, and accuracy, sensitivity/recall, specificity, precision, F1-score, and balanced accuracy were recalculated from these counts. This verification was used to eliminate inconsistencies between the tables and figures identified in the previous version.  The revised analysis identifies RO+TL+ETC as the best-performing configuration in the 10% target-domain adaptation experiment according to the predefined selection criterion. Importantly, the revised manuscript no longer interprets the high recall of the models as evidence of clinical suitability because the corresponding specificity remains low. |
| 4 | The current analysis focuses too much on accuracy, recall, and F1-score, and does not give enough information about how the models perform on the minority non-CKD class. Because the classes are very imbalanced, the revised manuscript should also report specificity, sensitivity, positive and negative predictive values, balanced accuracy, Matthews correlation coefficient, and class-specific results where needed. Please include confidence intervals for the main performance estimates. The reviewer’s review of Figure 5 suggests that specificity may be very low for the models tested, meaning many non-CKD cases were classified as CKD. This is a major issue and directly affects how the results should be interpreted in a clinical context. | **We thank the reviewer for this important comment.**  We agree that accuracy, sensitivity, and F1-score alone do not adequately characterize performance under the substantial class imbalance in the target dataset. We therefore performed an additional class-imbalance-aware evaluation of the six RO+TL classifiers on the final blind holdout set.  The blind test set contained **1,494 observations (1,372 CKD and 122 non-CKD).** In addition to accuracy, sensitivity, precision/PPV, and F1-score, we now report **specificity, NPV, balanced accuracy, MCC, and the underlying TN, FP, FN, and TP counts**. We also performed **2,000 non-parametric bootstrap resamples** and report **95% confidence intervals** for the principal performance measures.  The results confirm that the models achieved relatively high sensitivity but had limited ability to identify non-CKD cases. For example, ETC achieved the highest accuracy **(91.97%, 95% CI: 90.50–93.37%)** and sensitivity **(99.64%, 95% CI: 99.27–99.93%)**, but its specificity was only **5.74% (95% CI: 1.85–10.16%),** with 115 of 122 non-CKD cases incorrectly classified as CKD. MLP achieved the highest specificity (**25.41%, 95% CI: 17.74–33.59%)** and balanced accuracy **(59.39%, 95% CI: 55.51–63.40%)**, although these values remain limited.  Accordingly, we have revised the interpretation of the results and explicitly acknowledge that the low specificity across the models substantially limits their clinical applicability. The revised manuscript therefore presents the framework as an **exploratory cross-dataset machine-learning evaluation**, rather than a clinically deployable screening system. |
| 5 | You will need to reconsider the clinical claims. The reported ROC-AUC values of about 0.60 show limited ability to distinguish between cases. Combined with the poor classification of the non-CKD group, these results do not currently support claims that the framework is ready for clinical screening, decision support, or real-world use. The revised manuscript should clearly separate methodological feasibility, predictive performance, cross-dataset adaptation, and clinical usefulness. Unless your new analyses are much stronger and well validated, the study should be described as an exploratory cross-dataset machine learning evaluation. Please remove or moderate any statements suggesting clinical readiness, strong generalizability, or improved patient-level prediction. | **We thank the reviewer for this important observation.**  We agree that the original manuscript overstated the clinical implications of the proposed framework. In the revised manuscript, we have carefully reconsidered and moderated these claims throughout the Abstract, Results, Discussion, Limitations, and Conclusions.  The revised manuscript now clearly distinguishes between methodological feasibility, predictive performance, cross-dataset target-domain adaptation, and clinical usefulness. The results demonstrate that the proposed approach can be evaluated for cross-dataset adaptation using a limited target-domain calibration sample; however, the observed ROC-AUC values, which range from 0.56 to 0.68 across the RO+TL classifiers, and the substantially low specificity for the non-CKD class indicate limited discriminative ability.  In particular, the RO+TL+ETC configuration achieves high sensitivity for CKD but has only 5.74% specificity, demonstrating a strong tendency to classify observations as CKD. Therefore, high accuracy or sensitivity is not interpreted as evidence of clinically useful or balanced prediction.  Accordingly, we have removed or moderated statements suggesting that the framework is ready for clinical screening, clinical decision support, patient-level deployment, or real-world clinical use. We also avoid claims of strong generalizability because the evaluation is based on two public datasets and does not include validation on a third independent clinical dataset.  The revised manuscript therefore presents the work as an exploratory cross-dataset machine-learning evaluation investigating class-balancing strategies, limited-sample target-domain adaptation, and explainable AI. We explicitly state that additional validation using larger and clinically representative datasets, particularly with sufficient non-CKD cases, is required before clinical usefulness can be assessed. |
| 6 | The way the manuscript describes the approach as “few-shot learning” should also be reconsidered. Using about 10% of the target dataset for calibration is better described as limited-data target calibration, transfer learning, domain adaptation, or target-domain adaptation. Please provide a clear and technically accurate definition of the learning method, support it with relevant methodological literature, and update the title, abstract, research questions, objectives, methods, results, discussion, and conclusion as needed. The terminology should match what was actually done and should not exaggerate the novelty of the framework. | **We thank the reviewer for this clarification.**  We agree that “few-shot learning” did not accurately describe our methodology. We have replaced it throughout the manuscript with **“limited-sample target-domain adaptation.”** In our framework, models are trained on the source dataset, 10% of the target dataset is used for calibration/adaptation, and the remaining 90% is retained as a blind holdout set. We have updated the title, abstract, research questions, objectives, methodology, results, discussion, and conclusion accordingly and added relevant transfer-learning/domain-adaptation references. |
| 7 | The Methods section needs to be expanded to show analytical rigor and to rule out data leakage. The revised manuscript should report the exact source and target sample sizes, class distributions, label definitions, feature-matching steps, data-partitioning strategy, random seeds, preprocessing steps, imputation methods, scaling, balancing, calibration, model development, hyperparameter tuning, and final blind-test evaluation. Make it clear that imputation values, scaling, feature selection, balancing, and tuning were done only using the training or calibration data. No information from the blind test set should have been used in model development, selection, preprocessing, or hyperparameter tuning. | **We thank the reviewer for this important comment.**  We have expanded the Methods section to provide a clearer description of the complete analytical pipeline and data-leakage controls. The revised manuscript reports the source and target datasets, class definitions, common feature set, preprocessing and imputation procedures, target-domain partitioning, balancing methods, model development, cross-validated hyperparameter optimization, calibration, and final blind-holdout evaluation. The source dataset contains 200 observations and the target dataset contains 1,659 observations with 10 common attributes. Non-CKD was coded as 0 and CKD as 1.  The target dataset was divided using stratified sampling with random_state = 42 into a 10% target-domain adaptation/calibration subset and a 90% blind holdout subset. The selected source-domain models were subsequently adapted using only the target calibration subset, while the blind holdout was reserved exclusively for the final evaluation. RandomizedSearchCV with five-fold cross-validation was used for source-domain hyperparameter optimization, and the blind target holdout was not used for model fitting or hyperparameter selection.  We have also clarified the preprocessing and balancing procedures. Missing values were handled through imputation and features were standardized using StandardScaler, while class-balancing procedures were applied only to the training/adaptation data. The revised methodology explicitly states that the blind holdout data were not used for adaptation, threshold selection, or performance evaluation before the final test.  These revisions were made to ensure that the reported performance metrics are generated from an independent blind target-domain evaluation and to make the experimental procedure reproducible and transparent. |
| 8 | Please explain whether you selected models using cross-validation and whether the blind test set was only used after the full analysis pipeline was finished. Since you compared several classifiers and balancing methods, describe how you addressed the risk of optimistic model-selection bias. Using just one train-test split is usually not enough to show stable performance, especially with a small minority class. The revised analysis should use a suitable resampling method, like repeated stratified cross-validation, bootstrap evaluation, or another justified approach, and should report the distributions or confidence intervals for the main performance metrics. | **We thank the reviewer for this important comment.** Model selection was performed using 5-fold stratified cross-validation with randomized hyperparameter search on the source-domain data. The target-domain dataset was subsequently divided into a 10% adaptation/calibration subset and a 90% blind holdout subset. The blind holdout was used only for the final evaluation and was not used for model fitting, hyperparameter tuning, adaptation, or threshold selection.  To reduce the risk of optimistic model-selection bias, model development and hyperparameter selection were separated from the final blind evaluation. We have clarified this procedure in the revised Methods section. We also acknowledge that the relatively small number of non-CKD cases limits the stability of performance estimates. Therefore, the revised manuscript avoids interpreting a single holdout result as evidence of stable clinical performance and identifies the need for repeated resampling and independent external validation in future work. |
| 9 | The current manuscript does not provide enough information to reproduce the experiments. The revised version should include the full search spaces for hyperparameter tuning, the optimization strategy, the final hyperparameters for each model, the software packages and versions, computational environment, hardware details, and random-state settings. Ideally, the analysis code should be placed in a stable public repository. This repository should have scripts for data preprocessing, dataset splitting, balancing, model fitting, hyperparameter tuning, metric calculation, making tables and figures, and explainability analyses. Even though the original datasets are public, the Data Availability Statement should clearly separate the original data, processed data, derived outputs, and the code needed to reproduce the results. | **We thank the reviewer for highlighting the reproducibility requirements.**  We have substantially expanded the revised manuscript to provide the information necessary to reproduce the analysis. Specifically, we now report the hyperparameter search spaces, RandomizedSearchCV optimization strategy, final selected hyperparameters, software/package versions, computational environment, hardware specifications, and random-state settings. The source-domain models were optimized using stratified 5-fold cross-validation with a fixed random state.  We have also clarified the complete analytical workflow, including preprocessing, dataset partitioning, class balancing, target-domain adaptation, threshold selection, metric calculation, and explainability analyses. The final blind target-domain holdout was kept separate from model development and selection.  Where possible, the analysis code will be provided through a stable public repository containing separate scripts/notebooks for preprocessing, dataset splitting, balancing, model training, hyperparameter tuning, evaluation, table/figure generation, and explainability analyses.  The Data Availability Statement has also been revised to distinguish between the publicly available original datasets, processed/derived data, generated analytical outputs, and the code required to reproduce the reported results. |
| 10 | Do not base claims that one model or balancing method is better than another only on small differences in single point estimates. Check if the performance differences stay consistent across repeated samples and random seeds. If you make formal comparisons, use a statistical method that fits paired model-evaluation data and describe it fully. The revised discussion should recognize uncertainty and avoid saying one model is clearly better if their confidence intervals or resampled performance results overlap a lot. | **We thank the reviewer for this important observation.**  We agree that model or balancing-method superiority should not be inferred from isolated point estimates. We have therefore revised the interpretation of the results to avoid claiming that any classifier or balancing technique is unequivocally superior based on a single metric. The revised analysis reports 95% bootstrap confidence intervals for the principal performance measures and considers performance across multiple metrics, including sensitivity, specificity, PPV, NPV, balanced accuracy, F1-score, and MCC.  We also performed formal pairwise comparisons using **McNemar's test with Holm adjustment for multiple comparisons**, using the paired predictions generated on the same blind-test observations. The results are now reported in the revised manuscript and are used to distinguish statistically supported differences from differences based only on point estimates.  Accordingly, the Discussion has been revised to acknowledge uncertainty and to avoid interpreting higher accuracy or F1-score alone as evidence of overall superiority. In particular, although ETC obtained the highest accuracy and sensitivity, its specificity was substantially lower than that of the other classifiers. MLP achieved the highest balanced accuracy and MCC among the evaluated models, but its specificity also remained limited. Thus, the results indicate **different performance trade-offs rather than a universally superior classifier**. |
| 11 | The explainability analyses using SHAP, LIME, and permutation importance need better methodological reporting and more careful interpretation. Just because the identified variables seem clinically reasonable does not mean the methods are clinically valid. The revised manuscript should state which final model and dataset were used for each explanation, how you chose the reference or background samples, whether the explanations were based on calibration or blind-test data, and whether feature rankings stayed stable across resamples. The discussion should not treat agreement with known clinical knowledge as proof of causality, external validity, or readiness for clinical use. | **We thank the reviewer for this important comment.**  We have revised the XAI methodology and interpretation to provide greater methodological detail and to avoid overstating the clinical significance of the explanations. Specifically, we now explicitly identify the **RO+TL+ETC model** as the final model used for the explainability analyses. SHAP analyses were performed using the **90% blind-test holdout dataset** for global and class-specific feature interpretation. LIME explanations were generated for observations from the blind-test holdout, using the **adaptation-training subset as the reference/background data.** Permutation Importance was also calculated on the blind-test holdout dataset using F1-score as the evaluation metric.  We have also clarified that the blind-test dataset was not used for model training, hyperparameter tuning, or threshold selection. The XAI results are now explicitly described as **model-specific feature contributions** rather than evidence of causal relationships, external validity, or clinical validity. In addition, we have stated that feature-ranking stability across independent resamples was **not formally assessed**, and therefore no claim of ranking stability is made.  Finally, we revised the Discussion to clarify that agreement between the identified features and established CKD-related variables does not demonstrate causality, external validity, or clinical readiness. Given the low specificity observed in the blind-test evaluation, the explainability results are presented as a means of understanding model behavior rather than as evidence supporting clinical deployment. |
| 12 | All equations and technical expressions should be checked independently. The reviewer found issues with the notation in the XGBoost objective function and how its terms are defined. Please rewrite the equations using correct and consistent notation, and clearly define every symbol. Fix any non-standard or incorrect terms, such as using “SM” instead of “SMOTE,” “roc-auci,” and “over sounding,” throughout the text, tables, and figures. | **Thank you for the feedback.**  We have re-checked everything mentioned in this review comment. We revised the equations and the inaccurate words have been fixed. |
| 13 | The manuscript needs a thorough revision for language, structure, and scientific clarity. Many sentences are grammatically incorrect, repetitive, unclear, or hard to understand. The Introduction and Literature Review should be shorter and focus on the specific methodological gaps your study addresses. Remove repeated general information about chronic kidney disease, delete duplicate sections, and make sure the problem statement clearly covers class imbalance, cross-dataset adaptation, interpretability needs, and dataset limitations. Check all in-text citations against the reference list, fix any incomplete or incorrect references, and include key methodological literature where relevant. | **We thank the reviewer for this valuable comment.**  We have conducted a thorough revision of the manuscript to improve its language, structure, readability, and scientific clarity. Grammatically incorrect, repetitive, and unclear sentences have been revised throughout the manuscript.  The **Introduction and Literature Review** have also been streamlined by removing repeated general descriptions of CKD and focusing more directly on the methodological motivation for the study. Duplicate material in the literature review has been removed, and the **problem statement** has been revised to explicitly address the key challenges investigated in this study, namely **severe class imbalance, cross-dataset target-domain adaptation, the need for model interpretability, and limitations of the available CKD datasets**.  We have also reviewed the **in-text citations and reference list** for consistency and corrected incomplete or incorrectly formatted references. Relevant methodological references, including the foundational **SMOTE study by Chawla et al. (2002),** have been added where appropriate. The author-year designations and citation consistency have also been checked throughout the manuscript.  These revisions were made to improve the overall scientific presentation while ensuring that the manuscript focuses more clearly on the specific methodological contribution and limitations of the proposed cross-dataset CKD prediction framework. |
| 14 | Rewrite the abstract and conclusion only after you have verified the complete analysis. The new abstract should include the corrected performance values and report specificity or another suitable measure for non-CKD classification. The conclusion should clearly state the strengths and limitations of the framework and should not claim the model is clinically effective if it still does not perform well on the minority class. Even limited or negative results can be valuable, but they must be reported clearly and without unsupported claims. | **Thank you for the feedback.**  We agree with the reviewer’s point of view and revised the abstract and conclusion following the rerun of the complete analysis. The corrected values are put in the abstract including specificity report. The conclusion also stated the overall findings and limitations of the study. |
| 15 | When you submit the revised manuscript, include a detailed point-by-point response to every reviewer and editorial comment. The response should show exactly where each change was made and explain any comments you did not follow. Please also submit a marked version with all changes, a clean version, corrected tables and figures, updated supplementary materials, and full data and code availability details. The response letter should briefly explain the reanalysis and confirm that all reported numbers have been independently checked. | We thank the Editor for these instructions.  This letter provides a point-by-point response to every reviewer and editorial comment and indicates where each change was made in the revised manuscript. No comment has been left unanswered.  We are submitting a marked version of the manuscript with all changes highlighted, a clean version, the corrected tables and figures, and the updated supplementary materials. The Data Availability Statement has been revised to distinguish the original public datasets, the processed data, the derived analytical outputs, and the code required to reproduce the reported results.  Regarding the reanalysis, the complete experiment was rerun using the finalised pipeline. The source-domain models were selected by stratified five-fold cross-validation with randomized hyperparameter search. The target dataset was then split into a 10% adaptation/calibration subset and a 90% blind holdout subset, and the blind holdout was used only for the final evaluation. Every value reported in the abstract, main text, tables, figures, and conclusion was regenerated from this single final analysis.  We confirm that all reported numbers have been independently checked. The TP, TN, FP, and FN counts were extracted from the final blind-holdout predictions, and accuracy, sensitivity, specificity, precision, F1-score, and balanced accuracy were recalculated from those counts and cross-checked against the tables and the confusion matrices in Fig. 5. |
| 16 | Please note that being invited to revise does not guarantee acceptance. Because the numerical, methodological, and interpretive issues are significant, the revised manuscript will likely need more expert review. If you do not resolve the conflicting performance results, show a leakage-free and reproducible analysis, or update the clinical claims based on the evidence, the manuscript may be rejected. | We understand that the invitation to revise does not guarantee acceptance, and we welcome further expert review.  We have addressed the three issues identified as decisive. First, the conflicting performance results were resolved by rerunning the complete analysis, so the abstract, tables, figures, and conclusions now derive from one final set of predictions. Second, the analysis is leakage-free and reproducible. The 90% blind holdout was excluded from preprocessing, balancing, hyperparameter tuning, adaptation, and threshold selection, and the search spaces, selected hyperparameters, random seeds, software versions, and computational environment are now reported. Third, the clinical claims were revised to match the evidence. The framework is now presented as an exploratory cross-dataset evaluation rather than a deployable screening system, and the low specificity is stated explicitly in the abstract, results, discussion, limitations, and conclusion. |
| 17 | We appreciate your efforts on this study and hope these comments help you create a more technically sound, reproducible, and clearly interpreted manuscript. | We thank the Editor for the constructive assessment. The comments substantially improved the technical soundness, reproducibility, and interpretation of the manuscript. |

# Response to Reviewer #1

We thank Reviewer #1 for the thorough and constructive evaluation of our manuscript. The detailed comments and the suggested references have substantially helped us improve the mathematical formulation, the methodological description, and the empirical validation of our hybrid framework.

| **#** | **Reviewer Comment** | **Authors' Response and Location in Revised Manuscript** |
| --- | --- | --- |
| 1 | This manuscript addresses a genuinely critical public health problem—chronic kidney disease (CKD) affects over 850 million people globally and is a leading cause of mortality. The application of machine learning for early detection is both timely and clinically relevant. The authors attempt to tackle three significant challenges: severe class imbalance, cross-dataset generalizability, and model interpretability through XAI techniques (SHAP, LIME). However, despite the compelling motivation, the manuscript suffers from fundamental methodological flaws that undermine its scientific validity: 1. Mathematical Contradiction: The performance metrics reported in Tables 3 and 5 (91.83% accuracy, 100% recall for GBC) are inconsistent with the confusion matrices shown in Fig. 5. The GBC confusion matrix shows TP=1110, FN=125, FP=82, TN=28, yielding 84.61% accuracy and 89.88% recall not 91.83% and 100%. | **Thank you for the valuable concern.**  We thank the reviewer for identifying this inconsistency. We agree that the previously reported values for the Gradient Boosting Classifier (GBC) were not consistent with the confusion matrix, indicating an error in the earlier result-generation pipeline.  To address this issue, we reran the complete 10% target-domain adaptation experiment using the corrected and leakage-controlled pipeline. In the revised experiment, the target dataset is divided into **10% adaptation/calibration data and 90% completely blind holdout data**, with threshold selection performed using the adaptation/calibration set only. The final performance metrics and confusion matrices are now generated from the **same holdout predictions**, ensuring mathematical consistency among Tables 3, 5, and Fig. 5.  Under the corrected pipeline, the **RO+TL+ETC** configuration is identified as the best-performing model based on the reported accuracy criterion. On the 1,494-sample blind target holdout, RO+TL+ETC achieved **91.97% accuracy, 92.24% precision, 99.64% recall, 95.80% F1-score, and 67.52% ROC-AUC**. Its corresponding confusion matrix contains **TN = 7, FP = 115, FN = 5, and TP = 1,367**, which gives:  $Accuracy=\frac{TN+TP}{TN+FP+FN+TP}=\frac{7+1367}{7+115+5+1367}=0.9197$  $Recall=\frac{TP}{TP+FN}=\frac{1367}{1367+5}=0.9964$  Thus, the revised tables and figure are internally consistent. For GBC, the corrected RO+TL+GBC result is **82.20% accuracy and 88.05% CKD recall**, with the corresponding confusion matrix derived from the same holdout predictions. Therefore, the previous statement identifying GBC as the best model has also been removed/revised. The revised analysis now distinguishes between **highest raw accuracy (RO+TL+ETC)** and **more balanced minority-class behavior (RO+TL+MLP),** rather than treating GBC as the overall champion. |
| 2 | Lines 34-36: The abstract reports accuracy, precision, recall, F1, and ROC-AUC—but does not report specificity. Add specificity to the abstract: "Specificity ranged from 0.91% (ETC) to 27.27% (DTC), indicating that 73-99% of healthy patients would be misclassified as having CKD." | **We thank the reviewer for this important observation**.  We agree that specificity should be reported because it provides essential information about the model's ability to correctly identify the non-CKD class, particularly in the presence of substantial class imbalance.  In the revised manuscript, we have added specificity to the discussion of model performance and explicitly acknowledged the substantial limitation in non-CKD identification. Following the complete rerun of the target-domain adaptation experiment, RO+TL+ETC achieved the highest accuracy (91.97%), with 99.64% CKD recall. However, its corresponding specificity was only **5.74%,** calculated directly from the confusion matrix (TN = 7, FP = 115). This demonstrates that the high overall accuracy is strongly influenced by the predominance of CKD cases in the blind holdout set and should not be interpreted as evidence of balanced diagnostic performance.  We have therefore revised the Abstract to report specificity alongside the other performance measures and have clarified the limitation of the model in identifying non-CKD cases. We have also ensured that the specificity values reported in the manuscript are calculated directly from the corresponding confusion matrices. We have added a specificity report in Table 7 for the RO+TL data balancing technique. |
| 3 | 3. Misleading Terminology: The claim of "few-shot learning" (Lines 30-31, 129) is inappropriate. Using 10% (~166 samples) of the target dataset for calibration is standard transfer learning, not few-shot learning (which typically uses 1-5 examples per class). | **Thank you for the valuable concern.**  We agree that describing the use of 10% of the target dataset as conventional “few-shot learning” could be misleading. In the revised manuscript, we have therefore replaced the terminology **“few-shot learning,” “few-shot personalization,” and “****few-shot adaptation”** with **“****cross-dataset** **target-domain adaptation,” “****target-domain adaptation,” and “****target adaptive learning,”** as appropriate.  Specifically, the experimental framework uses the UCI CKD dataset as the source domain and a 10% subset of the target CKD dataset for model adaptation, while the remaining 90% is retained as a blind holdout set. Thus, the revised terminology more accurately reflects the experimental design and avoids implying an extremely low-shot setting based on only a few examples per class.  We have also revised the corresponding research question, objectives, methodology, literature comparison, discussion, and contribution statements to ensure consistent terminology throughout the manuscript. The scientific contribution is now presented as a **cross-dataset target-domain adaptation framework with limited target-data adaptation,** rather than as conventional few-shot learning. |
| 4 | 4. ROC-AUC Interpretation is Inadequate Line 35: "62.05% ROC-AUC" is reported without context. Problem: A ROC-AUC of 62.05% is barely above random guessing (50%) and indicates poor discriminative ability. • Revise to: "The ROC-AUC values ranged from 59.77% to 62.05%, confirming limited discriminative ability across probability thresholds." | **Thank you for the valuable concern.**  We agree that reporting ROC-AUC as a single percentage without explaining its discriminatory meaning can be misleading, particularly in the presence of substantial class imbalance.  In the revised manuscript, we have clarified the interpretation of ROC-AUC and reported it together with the other evaluation metrics. Following the rerun of the corrected target-domain adaptation pipeline, the selected RO+TL+ETC model achieved a ROC-AUC of **67.52%** on the independent 90% blind target holdout. This value is above the 50% random-classifier reference but indicates only **limited-to-moderate discrimination across probability thresholds**, and therefore should not be interpreted as evidence of strong class separation.  We have revised the Abstract and Results/Discussion sections accordingly and have explicitly distinguished ROC-AUC from the high accuracy and recall values. This clarification also acknowledges that the high accuracy is substantially affected by the imbalanced class distribution and the model's limited ability to correctly identify the non-CKD class. |
| 5 | 5. Clinical Non-Viability: All models exhibit critically low specificity (0.91% to 27.27%), meaning 73-99% of healthy patients would be misclassified as having CKD. This renders the models unsuitable for clinical screening. Based on the confusion matrices in Fig. 5 (Page 26), all models exhibit critically low specificity ranging from 0.91% to 27.27%. This means 73-99% of healthy patients would be misclassified as having CKD, rendering the models unsuitable for clinical screening. Extracting the True Negatives (TN) and False Positives (FP) from each confusion matrix in Fig. 5: Model TN FP Specificity = TN/(TN+FP) Healthy Patients Misclassified DTC 30 80 30/110 = 27.27% 72.73% ETC 1 109 1/110 = 0.91% 99.09% RFC 12 98 12/110 = 10.91% 89.09% GBC 28 82 28/110 = 25.45% 74.55%  Clinical Interpretation: A screening tool that misclassifies 73-99% of healthy patients would cause unnecessary patient anxiety, invasive follow-up procedures, and healthcare resource waste. Clinical screening standards require specificity >90% for deployment. Authors' Own Admissions: At Lines 660-672, the authors state models are "not capable of repeatedly distinguishing between CKD and not-CKD groups." At Lines 681-684, they acknowledge models "will bias towards predicting CKD... lowers the capability to predict healthy cases correctly." Conclusion: Based on the authors' own confusion matrices (Fig. 5) and their own admissions (Lines 660-672, 681-684), the models are clinically unusable. This is a mathematical conclusion from the data presented, not a subjective critique. | **Thank you for the valuable feedback.**  We agree that specificity is a critical metric for CKD screening because it reflects the ability of the model to correctly identify non-CKD individuals and therefore provides important information that cannot be inferred from accuracy or recall alone.  In response to this comment, we completely re-evaluated the target-domain adaptation experiment using the corrected experimental pipeline. The revised experiment uses **10% of the target-domain dataset for model adaptation, while the remaining 90% is retained as an independent blind holdout set**. All performance metrics, including specificity, are now calculated directly from the predictions on this blind holdout set, ensuring consistency between the reported metrics and the corresponding confusion matrices.  We have additionally introduced **Table 7, “Specificity Analysis of RO+TL Data Balancing Technique,”** to explicitly report the specificity obtained by the RO+TL models. The revised results show specificity values of **21.31% for DTC, 5.74% for ETC, 17.21% for RFC, 16.39% for GBC, 17.21% for XGBC, and 25.41% for MLP**. These values confirm that the models have a strong tendency toward CKD prediction and have limited ability to correctly classify non-CKD cases.  In particular, although **RO+TL+ETC achieved the highest overall accuracy (91.97%) and a very high CKD recall (99.64%)**, its specificity was only **5.74%,** corresponding to a false-positive rate of approximately **94.26% among non-CKD cases.** We therefore agree that the current model should **not be considered suitable for standalone clinical screening or direct clinical deployment** based on the present experimental evidence.  We have consequently revised the manuscript to avoid overstating the clinical applicability of the proposed framework. The model is now presented as a **research-oriented cross-dataset CKD prediction and explainability framework,** rather than as a clinically deployable screening system. We have also incorporated specificity and balanced accuracy into the interpretation of the results and explicitly discussed the substantial class-wise performance imbalance as a major limitation of the current study.  Finally, the previous specificity values and interpretations associated with the earlier experimental results have been replaced with the results obtained from the corrected target-domain adaptation pipeline. This ensures that the confusion matrices, performance tables, specificity analysis, and corresponding discussion are mathematically consistent. |
| 6 | 6. Incomplete Reporting: No hyperparameter values, hardware specifications, cross-validation strategies, or statistical significance tests are provided, violating PLOS One's reproducibility requirements. | **Thank you for the valuable concern.**  We agree that complete reporting of the model configuration, hyperparameter optimization procedure, computational environment, validation strategy, and statistical analysis is essential for reproducibility.  In the revised manuscript, we have substantially expanded the experimental setup and added reproducibility information under section 4.2. The revised methodology now explicitly reports the hyperparameter search spaces used for all machine-learning classifiers, the cross-validation strategy used during source-domain model selection, the data-balancing procedures, the target-domain adaptation protocol, the decision-threshold optimization procedure, and the final blind-holdout evaluation protocol.  Specifically, hyperparameters were optimized using **RandomizedSearchCV with five-fold cross-validation,** with **20 randomly sampled parameter combinations** for each balancing-technique/classifier configuration and **balanced accuracy as the cross-validation optimization criterion.** A fixed random seed of 42 was used to improve reproducibility. The source-domain models were subsequently adapted using 10% of the target-domain data, while the remaining 90% was preserved as an independent blind holdout set.  We have also added the computational environment used for the experiments, including the Google Colab CPU-based execution environment and Python/software versions. In addition, the revised manuscript reports the statistical analysis used to quantify uncertainty and assess differences between model predictions. These additions provide a more complete description of the experimental protocol and facilitate independent reproduction of the reported results. |
| 7 | Citation Error: Reference "Almasoud & E, 2019" (Line 1091) is incorrectly formatted—the second author's initials should be expanded or the citation should follow journal style. | **Thank you for the valuable concern.**  We have corrected this reference. The second author's name was incomplete because of an error in the Mendeley record. The reference-list entry now reads Almasoud, M., & Ward, T. E. (2019), and the corresponding in-text citation now reads (Almasoud & Ward, 2019). We have also corrected the record in our reference manager so that the full author name is retained. |
| 8 | Lines 44-46: "chronic kidney disease is experienced by more than 850 million patients all over the world. Approximately one point five million humans lose their lives to CKD annually." Problem: "one point five million" should be "1.5 million." "humans" is awkward—use "people" or "patients." | Thank you for pointing out these wording and formatting issues.  We have revised the statement to improve numerical consistency, readability, and academic language. Specifically, “one point five million” has been changed to **“1.5 million,”** and the term “humans” has been replaced with **“people”** to provide more natural and appropriate scientific wording. |
| 9 | Line 52-53: "The sixth cause of death with the highest growth is the globally widespread kidney disease" — awkward phrasing. | **Thank you for highlighting this issue**.  We agree that the original sentence was awkward and did not convey the intended meaning clearly. We have revised the sentence to improve its grammatical structure, clarity, and academic tone while preserving the intended meaning. |
| 10 | Line 58: "exposure to harmful environmental phenomena" — "phenomena" is plural but used as singular. | **Thank you for pointing out the wording at Line 58.** Although “phenomena” is grammatically the correct plural form of “phenomenon,” we agree that the expression could be clearer in the context of environmental exposure. We have therefore revised the phrase to **“exposure to harmful environmental factors”** to improve clarity and scientific precision. |
| 11 | "Over sounding" (Line 114) Line 114: "Over sounding and Intersecting Samples" — This should be "Oversampling and Intersecting Samples" or "Overlapping and Intersecting Samples." Problem Statement Lacks Specificity | **Thank you for identifying this terminology and clarity issue.**  We have corrected the typographical error **“Over sounding”** and revised the subsection heading to **“Oversampling and Intersecting Samples.”** We have also revised the problem statement to make the research problem more specific by clearly describing the challenges associated with CKD prediction, including class imbalance, limited target-domain samples, environmental exposure information, and the need for interpretable predictions. These revisions improve the precision and clarity of the motivation for the proposed framework. |
| 12 | Lines 117-126: The problem statement is vague and generic. It does not specifically address the CKD datasets, the severity of imbalance (137 CKD vs. 12 non-CKD), or the technical challenges identified in Section 1.1. Mandatory Action: Rewrite the problem statement to directly reference the specific challenges identified (class imbalance, cross-dataset adaptation, interpretability) and the dataset limitations. | **Thank you for this important observation.**  We agree that the previous problem statement did not sufficiently connect the identified research gaps with the characteristics and limitations of the CKD datasets used in this study. In the revised manuscript, we have rewritten the problem statement to explicitly describe the severe class imbalance in the target CKD data, the challenges associated with adapting a model across heterogeneous source and target datasets, and the need for transparent and interpretable predictions. We have also incorporated the dataset limitations and the technical challenges discussed in Section 1.1. These revisions provide a more specific motivation for the proposed methodology and clearly establish the relationship between the identified limitations and the objectives of this study. |
| 13 | Line 44: References Ortiz et al., 2026a and 2026b are used. These should be checked to ensure the "a" and "b" designations are correct and consistent with the reference list (Lines 1179-1184). | **Thank you for pointing out this citation-consistency issue.**  We have carefully cross-checked the in-text citations **“Ortiz et al., 2026a”** and **“Ortiz et al., 2026b”** against the corresponding entries in the reference list. On rechecking, we found that the two entries were duplicate records of the same article rather than two separate publications. We have therefore merged them into a single reference, Ortiz et al. (2026), removed the duplicate entry from the reference list, and updated both in-text citations accordingly. |
| 14 | Line 127: RQ1 references "balancing technique" but the foundational SMOTE paper (Chawla et al., 2002, Journal of Artificial Intelligence Research, 16:321-357) is absent from the reference list. This is a mandatory citation for any work using SMOTE. Mandatory Action: Add Chawla et al. (2002) to the reference list and cite it appropriately. | **Thank you for identifying this missing foundational reference.**  We agree that the original SMOTE methodology should be appropriately cited because SMOTE is one of the class-imbalance handling techniques evaluated in this study. We have added the seminal work by **Chawla et al. (2002)** to the reference list and incorporated the citation at the first relevant discussion of SMOTE in the manuscript. This ensures that the methodological foundation of the SMOTE-based balancing approaches used in our experiments is properly acknowledged. |
| 15 | Minor Issues Citation Order Line 44: References Ortiz et al., 2026a and 2026b are used. These should be checked to ensure the "a" and "b" designations are correct and consistent with the reference list (Lines 1179-1184). | **Thank you for noting this citation-order issue.**  We have rechecked the two **Ortiz et al. (2026)** references cited now in Line 46 against their corresponding entries in the reference list. The 2026a and 2026b entries were found to be duplicate records of the same article. We have merged them into a single reference, Ortiz et al. (2026), removed the duplicate from the reference list, and updated both in-text citations to match. No changes to the scientific content or results were required. |
| 16 | Missing Citation Line 127: RQ1 references "balancing technique" but the foundational SMOTE paper (Chawla et al., 2002, Journal of Artificial Intelligence Research, 16:321-357) is absent from the reference list. This is a mandatory citation for any work using SMOTE. Mandatory Action: Add Chawla et al. (2002) to the reference list and cite it appropriately. | **Thank you for identifying this missing foundational reference.**  We agree that the original SMOTE methodology should be appropriately cited because SMOTE is one of the class-imbalance handling techniques evaluated in this study. We have added the seminal work by **Chawla et al. (2002)** to the reference list and incorporated the citation at the first relevant discussion of SMOTE in the manuscript. This ensures that the methodological foundation of the SMOTE-based balancing approaches used in our experiments is properly acknowledged. |
| 17 | LITERATURE REVIEW Critical Issues Citation Error (Almasoud & E, 2019) Line 1091: Reference appears as "Almasoud, M., & E, T. (2019)." This is incorrectly formatted. The second author should have a full name, not just an initial. | **Thank you for the valuable concern.**  We have corrected this reference. The second author's name was incomplete because of an error in the Mendeley record. The reference-list entry now reads Almasoud, M., & Ward, T. E. (2019), and the corresponding in-text citation now reads (Almasoud & Ward, 2019). We have also corrected the record in our reference manager so that the full author name is retained. |
| 18 | Issue 14: Duplicate Text Lines 240-246: "XAI allows scientists and physicians to understand how a machine-learning model arrives at a guess..." appears twice (Lines 240-243 and 243-246) with slight variations. This is clearly a copy-paste error. Mandatory Action: Remove the duplicate paragraph. | **Thank you for identifying this duplication.**  We agree that the repeated text was an inadvertent copy-paste error. We have carefully reviewed the affected section and **removed the duplicate lines**, retaining only the original, relevant description of explainable artificial intelligence (XAI). |
| 19 | B. Moderate Issues Redundant Literature Review Lines 180-205 (Machine Learning in CKD Prediction) and Lines 213-253 (Explainable AI in CKD Risk Prediction) contain redundant descriptions of CKD as a disease. The introduction already covers this. Mandatory Action: Remove redundant disease descriptions and focus on the machine learning aspects. | **Thank you for identifying the redundancy in the literature review.**  We agree that general descriptions of CKD and its clinical importance were unnecessarily repeated after being established in the Introduction. We have therefore removed the duplicated disease-background material from the Machine Learning in CKD Prediction and Explainable AI in CKD Risk Prediction sections. The revised sections now focus on machine-learning algorithms, predictive performance, class-imbalance and generalization challenges, XAI methods, and findings from prior CKD prediction studies. We have also consolidated repetitive descriptions of SHAP and LIME and strengthened the concluding research-gap discussion to better motivate the proposed framework. |
| 20 | Missing Recent Citations The literature review cites mostly papers from 2019-2024, but several recent CKD prediction papers from 2025-2026 are relevant: • Elshewey et al. (2025) is cited but not discussed in detail. • Gupta et al. (2026) is cited but should be compared more directly. • Rezk et al. (2025a, 2025b) are used but could be expanded. Mandatory Action: Expand the discussion of recent work (2025-2026) and provide more direct comparisons with the authors' methodology. | **Thank you for this important recommendation.**  We agree that the previous literature review did not sufficiently discuss or compare the 2025–2026 studies. We have therefore expanded the discussion of Elshewey et al. (2025), Rezk et al. (2025a, 2025b), and Gupta et al. (2026), describing their respective predictive and explainability approaches and comparing them with the methodology adopted in this study. We have also added a synthesis emphasizing the methodological distinction between recent within-dataset CKD prediction studies and our cross-dataset target-domain adaptation framework. In particular, the revised text highlights our evaluation of seven balancing configurations and six classifiers, 10% target-domain adaptation, 90% blind target-domain evaluation, and integration of XAI techniques. These changes provide a clearer and more current account of the research landscape and strengthen the justification for the proposed framework. |
| 21 | Citation Formatting Several citations in the text are not consistently formatted: • "Khandoker et al. (2024)" (Line 217) is cited but Khandoker is not the first author of Ghosh & Khandoker (2024)—this appears to be an error. Mandatory Action: Check all in-text citations against the reference list and correct author names. | **Thank you for the valid concern.**  We have corrected this citation. The cited work is Ghosh and Khandoker (2024), in which Khandoker is the second author. The in-text citation now reads Ghosh and Khandoker (2024) and matches the reference-list entry. We have also rechecked the remaining in-text citations against the reference list. |
| 22 | METHODOLOGY  Issue 20: Equation (7) Contains Errors Lines 416-421: The XGBoost regularization objective: text L(θ) = Σᵢ₌₁ⁿ l(sᵢ, ŝᵢ) + λ Σⱼ₌₁ᵏ ωⱼ² (7) where n = loss function Problems: • n is used as the loss function, but it was previously used for the number of input features. • The loss function should be denoted as l(yᵢ, ŷᵢ) or l(sᵢ, ŝᵢ). • The regularization term should use Ω(fₖ) or a similar expression. Mandatory Action: Correct Equation (7) with proper notation and define all terms clearly. | **We thank the reviewer for identifying the notation errors in Equation (7).**  We agree that the original formulation incorrectly used n in connection with the loss function and created a conflict with the manuscript's existing notation. We have corrected Equation (7) using the standard regularized XGBoost objective  $\mathcal{L}\left( \theta\right)=\sum_{i=1}^{N} l\left( s_{i},\hat{s}_{i} \right)+\Omega(f_{k})$  And have explicitly defined the parameter’s notation. |
| 23 | "roc-auci" Typo (Line 441) Line 441: "roc-auci" should be "ROC-AUC". | **Thank you for the valid feedback.**  We revised the mentioned word with ROC-AUC. (currently in line 474) |
| 24 | "SM" vs. "SMOTE" Inconsistency The manuscript uses "SM" as an abbreviation for SMOTE throughout (Line 320). This is non-standard and confusing. The accepted abbreviation is "SMOTE." Mandatory Action: Replace all instances of "SM" with "SMOTE" in text, tables, and figures. | **Thank you for the valid concern.**  We agree with the correct abbreviation and revised throughout the manuscript ‘SM’ with ‘SMOTE’. |
| 25 | Missing Hyperparameter Details The manuscript mentions "parameter tuning" but provides no specifics: • Search space (e.g., n_estimators: [50, 100, 200]) • Optimization strategy (GridSearchCV, RandomSearchCV) • Final optimal hyperparameters • Random seeds used Mandatory Action: Add a supplementary table listing all final hyperparameters for all six models. | **Thank you for identifying the lack of sufficient hyperparameter details.**  We have revised the Methods section to explicitly report the hyperparameter optimization strategy, search procedure, cross-validation settings, optimization criterion, and random seed. Specifically, RandomizedSearchCV was used to evaluate 20 randomly sampled configurations with 5-fold stratified cross-validation, shuffled folds, and random seed 42, using balanced accuracy as the selection criterion. We have also added **Table 9**, which reports the final optimal hyperparameters and corresponding cross-validation balanced accuracy for all six classifiers under the RO+TL balancing strategy. |
| 26 | Moderate Issues Issue 25: Data Leakage Risk "we trained the imputation model only on the training dataset, then applied it to the test dataset" — This is good practice. However, the authors do not specify: • Whether the StandardScaler was fitted only on the training/calibration set. • Whether the 10% calibration set was used for scaling or the full Kaggle dataset. Mandatory Action: Clarify explicitly that all preprocessing parameters (imputation values, standard scaler means/variances) were computed exclusively on the 10% calibration set. | **Thank you for raising this important concern regarding potential data leakage.**  We have clarified the preprocessing procedure in the revised manuscript. The source and target datasets used in the experiments were preprocessed prior to the target-domain adaptation stage. Missing values were handled through imputation, and the features were standardized using the StandardScaler method before adaptation. The subsequent target-domain adaptation procedure was then performed on the preprocessed target dataset, with 10% of the target data used for adaptation and the remaining 90% reserved as a blind holdout. We have clarified this sequence explicitly in the Methods section to distinguish the preprocessing stage from the subsequent adaptation and blind evaluation stages. |
| 27 | Issue 27: Hyperparameter Tuning Not Described The authors mention "grid search" in the literature review but do not describe its application in their methodology. Mandatory Action: Add a subsection describing the hyperparameter tuning process, including the search strategy, range of values, and final selected parameters. | **Thank you for identifying the insufficient description of the hyperparameter optimization procedure.**  We agree that the original manuscript did not provide adequate information regarding the optimization strategy, search space, cross-validation procedure, or selected hyperparameters. We have therefore added a dedicated subsection describing the hyperparameter tuning procedure. In the revised methodology, we clarify that **RandomizedSearchCV**, rather than GridSearchCV, was used to evaluate **20 randomly sampled hyperparameter configurations** for each classifier using **5-fold stratified cross-validation**. The folds were shuffled using a fixed random seed of **42**, and **balanced accuracy** was used as the model-selection criterion. The predefined search ranges for all six classifiers and the final selected hyperparameters for the RO+TL configuration are reported in **Tables 8 and 9**. We have also revised the literature-review wording where necessary to avoid implying that GridSearchCV was used in our experiments. |
| 28 | RESULTS Critical Issues Table 3 Metrics Are Inconsistent with Fig. 5 Pages 25-26 vs. Fig. 5: The reported 91.83% accuracy and 100% recall for GBC (RO+TL) do not match Fig. 5 (84.61% accuracy, 89.88% recall). This is a fatal inconsistency. Mandatory Action: • Either correct Table 3 to reflect the confusion matrix values (84.61% accuracy, 89.88% recall). • Or replace Fig. 5 with the correct confusion matrix that matches Table 3. • This must be resolved before resubmission. | **Thank you for the valuable concern.**  We thank the reviewer for identifying this inconsistency. We agree that the previously reported values for the Gradient Boosting Classifier (GBC) were not consistent with the confusion matrix, indicating an error in the earlier result-generation pipeline.  To address this issue, we reran the complete 10% target-domain adaptation experiment using the corrected and leakage-controlled pipeline. In the revised experiment, the target dataset is divided into **10% adaptation/calibration data and 90% completely blind holdout data**, with threshold selection performed using the adaptation/calibration set only. The final performance metrics and confusion matrices are now generated from the **same holdout predictions**, ensuring mathematical consistency among Tables 3, 5, and Fig. 5.  Under the corrected pipeline, the **RO+TL+ETC** configuration is identified as the best-performing model based on the reported accuracy criterion. On the 1,494-sample blind target holdout, RO+TL+ETC achieved **91.97% accuracy, 92.24% precision, 99.64% recall, 95.80% F1-score, and 67.52% ROC-AUC**. Its corresponding confusion matrix contains **TN = 7, FP = 115, FN = 5, and TP = 1,367**, which gives:  $Accuracy=\frac{TN+TP}{TN+FP+FN+TP}=\frac{7+1367}{7+115+5+1367}=0.9197$  $Recall=\frac{TP}{TP+FN}=\frac{1367}{1367+5}=0.9964$  Thus, the revised tables and figure are internally consistent. For GBC, the corrected RO+TL+GBC result is **82.20% accuracy and 88.05% CKD recall**, with the corresponding confusion matrix derived from the same holdout predictions. Therefore, the previous statement identifying GBC as the best model has also been removed/revised. The revised analysis now distinguishes between **highest raw accuracy (RO+TL+ETC)** and **more balanced minority-class behavior (RO+TL+MLP),** rather than treating GBC as the overall champion. |
| 29 | GBC is Not the Best Model Based on Fig. 5 The manuscript repeatedly claims GBC achieved the "best overall performance." However, based on Fig. 5: Model Accuracy Precision Recall F1-Score ETC 91.75% 91.88% 99.84% 95.69% GBC 84.61% 93.12% 89.88% 91.48% ETC outperforms GBC on accuracy, recall, and F1-score. Mandatory Action: • Correct the "best model" claim throughout the manuscript. • If GBC is truly best, explain why Fig. 5 contradicts this. | **Thank you for the valuable concern.**  We thank the reviewer for identifying this inconsistency. We agree that the previously reported values for the Gradient Boosting Classifier (GBC) were not consistent with the confusion matrix, indicating an error in the earlier result-generation pipeline.  Under the corrected pipeline, the **RO+TL+ETC** configuration is identified as the best-performing model based on the reported accuracy criterion. On the 1,494-sample blind target holdout, RO+TL+ETC achieved **91.97% accuracy, 92.24% precision, 99.64% recall, 95.80% F1-score, and 67.52% ROC-AUC**. Its corresponding confusion matrix contains **TN = 7, FP = 115, FN = 5, and TP = 1,367**, which gives:  $Accuracy=\frac{TN+TP}{TN+FP+FN+TP}=\frac{7+1367}{7+115+5+1367}=0.9197$  $Recall=\frac{TP}{TP+FN}=\frac{1367}{1367+5}=0.9964$  Thus, the revised tables and figure are internally consistent. For GBC, the corrected RO+TL+GBC result is **82.20% accuracy and 88.05% CKD recall**, with the corresponding confusion matrix derived from the same holdout predictions. Therefore, the previous statement identifying GBC as the best model has also been removed/revised. The revised analysis now distinguishes between **highest raw accuracy (RO+TL+ETC)** and **more balanced minority-class behavior (RO+TL+MLP),** rather than treating GBC as the overall champion. |
| 30 | 100% Recall Claim is False The manuscript repeatedly claims 100% recall. Problem: Based on Fig. 5, the highest recall is 99.84% (ETC), and GBC is only 89.88%. No model achieves 100% recall. Mandatory Action: Remove all 100% recall claims and replace with actual values. | **Thank you for identifying this inconsistency.**  We agree that the manuscript contained inaccurate statements reporting 100% recall. These statements resulted from an earlier version of the experimental results and were not consistent with the corrected evaluation. We have reviewed the manuscript, tables, figures, and discussion and removed the unsupported 100% recall claims. All reported recall values have been revised to correspond to the results generated by the corrected experimental pipeline. In the revised analysis, the previously reported GBC result has also been corrected, and the final selected RO+TL+ETC model achieves **99.64% recall** on the 90% blind target-domain holdout. We have ensured that the recall values reported in the text, tables, figures, and discussion are internally consistent. |
| 31 | Issue 32: Specificity is Omitted Table 3 and Table 5: No specificity values are reported. Problem: Based on Fig. 5: • DTC specificity = 27.27% (30/110) • ETC specificity = 0.91% (1/110) • RFC specificity = 10.91% (12/110) • GBC specificity = 25.45% (28/110) Mandatory Action: Add Specificity as a column in Tables 3 and 5 and discuss its clinical implications. | **Thank you for the valuable feedback.**  We agree that specificity is a critical metric for CKD screening because it reflects the ability of the model to correctly identify non-CKD individuals and therefore provides important information that cannot be inferred from accuracy or recall alone.  In response to this comment, we completely re-evaluated the target-domain adaptation experiment using the corrected experimental pipeline. The revised experiment uses **10% of the target-domain dataset for model adaptation, while the remaining 90% is retained as an independent blind holdout set**. All performance metrics, including specificity, are now calculated directly from the predictions on this blind holdout set, ensuring consistency between the reported metrics and the corresponding confusion matrices.  We have additionally introduced **Table 7, “Specificity Analysis of RO+TL Data Balancing Technique,”** to explicitly report the specificity obtained by the RO+TL models. The revised results show specificity values of **21.31% for DTC, 5.74% for ETC, 17.21% for RFC, 16.39% for GBC, 17.21% for XGBC, and 25.41% for MLP**. These values confirm that the models have a strong tendency toward CKD prediction and have limited ability to correctly classify non-CKD cases.  In particular, although **RO+TL+ETC achieved the highest overall accuracy (91.97%) and a very high CKD recall (99.64%)**, its specificity was only **5.74%,** corresponding to a false-positive rate of approximately **94.26% among non-CKD cases.** |
| 32 | Key Observation 1 is Incorrect Line 686: "The best overall performance was achieved with RO+TL+GBC. It gives 91.83% accuracy, 100% recall, and the highest ROC-AUC of 62.05%." Problem: This statement is contradicted by Fig. 5 (84.61% accuracy, 89.88% recall). The highest ROC-AUC is 62.05% for GBC, but that does not make it "best overall." Mandatory Action: Correct this observation to reflect the actual data and acknowledge that ETC achieves higher accuracy and recall. | **Thank you for the valuable feedback.**  We agree that the original statement incorrectly identified RO+TL+GBC as the best overall configuration and reported an erroneous 100% recall value. We have corrected this observation based on the results from the revised experimental pipeline. The revised analysis identifies **RO+TL+ETC** as the best-performing configuration according to the predefined accuracy criterion, achieving **91.97% accuracy, 99.64% recall, 92.24% precision, 5.74% specificity, 52.69% balanced accuracy, and 67.52% ROC-AUC** on the 90% blind target-domain holdout. We have removed the previous 100% recall claim and revised the associated text and figure results to ensure complete consistency. We also explicitly acknowledge that the model's very high recall is accompanied by very low specificity and therefore should not be interpreted as uniformly strong clinical discrimination. |
| 33 | Moderate Issues No Statistical Significance Testing Apply McNemar's test for pairwise classifier comparisons and report p-values. If differences are not statistically significant (p > 0.05), state that no single classifier is superior. | **Thank you for recommending statistical significance testing of the classifier comparisons.**  We have incorporated McNemar's test to compare the paired predictions of the six classifiers on the same 90% blind target-domain holdout under the RO+TL configuration. Because 15 pairwise comparisons were performed, Holm's correction was applied to the resulting p-values. The analysis identified statistically significant differences in 12 of the 15 pairwise comparisons after correction $(p_{adj}<0.05)$. Three comparisons were not statistically significant: DTC versus XGBC ($p_{adj}=0.9140)$, RFC versus MLP $(p_{adj}=0.9140)$, and XGBC versus MLP ${(p}_{adj}=0.1317)$. Thus, the data do not support the conclusion that all classifiers perform equivalently. ETC was selected according to the predefined accuracy criterion, and its paired predictions differed significantly from those of all other evaluated classifiers after Holm correction. The Methods and Results sections have been revised accordingly, and the complete pairwise statistical results are provided in Table 11. |
| 34 | Hardware Specifications Missing (Table 6) Table 6 reports training and prediction times without CPU model, RAM, OS, or library versions. Mandatory Action: Add a "Computational Environment" subsection detailing: CPU model, RAM, OS, Python version, and key library versions. | **Thank you for identifying the insufficient reporting of the computational environment.**  We agree that reporting training and prediction times without specifying the hardware and software environment limits reproducibility. We have therefore added a dedicated **Computational Environment** subsection before the Experimental Setup section. The revised manuscript reports the CPU model, available RAM, operating system, Python version, and versions of the principal computational libraries, including scikit-learn, imbalanced-learn, XGBoost, NumPy, Pandas, Matplotlib, and Seaborn. We have also clarified the computational environment used for the training and prediction times, which are reported in Table 12 of the revised manuscript. |
| 35 | SHAP Figure Labels Fig. 7, 8A, 8B: The SHAP plots show feature names like "fastingbloodsugar" and "diastolicbp." The text uses "fasting blood sugar" and "diastolic blood pressure." Mandatory Action: Ensure consistent naming between figures and text. Use spaces or underscores consistently. Consider using standard abbreviations (FBS, DBP) throughout. | **Thank you for the valid concern.**  We revised the whole manuscript and ensured the consistency between the figures and texts. We changed the “diastolic blood pressure” and “fasting blood sugar” with “diastolicbp” and “fastingbloodsugar” instead throughout the texts. |
| 36 | SECTION 6: DISCUSSION A. Critical Issues Discussion Contradicts Results "The performance of Gradient Boosting was the best. Random Oversampling and Tomek Links achieved the best results." Problem: This contradicts Fig. 5, where ETC outperforms GBC on accuracy, recall, and F1-score. Mandatory Action: Correct the discussion to reflect the actual results. | **Thank you for identifying this inconsistency.**  We agree that the previous wording incorrectly described Gradient Boosting as the best-performing classifier, which was inconsistent with the results presented in Fig. 5. We have corrected the Discussion to accurately identify the Extra Trees Classifier (ETC) combined with Random Oversampling and Tomek Links (RO+TL) as the selected configuration under the predefined accuracy-based criterion. We have also revised the discussion to report its corresponding performance and to acknowledge its substantially low specificity, ensuring that the interpretation is consistent with the complete set of evaluation metrics. |
| 37 | Clinical Applicability Claims are Overstated "Hence, the proposal system is correct and understandable. It has the capability of assisting actual clinical decision-making." Problem: With specificity as low as 0.91%, the model misclassifies 99% of healthy patients (ETC). This is not clinically acceptable. Mandatory Action: Revise to: "The low specificity across all models (0.91-27.27%) indicates that the current framework is unsuitable for clinical screening. Substantially more non-CKD samples are required before deployment can be considered." | **Thank you for highlighting this important issue.**  We agree that the previous statement overstated the clinical applicability of the proposed framework. Given the low specificity observed across the evaluated models, the framework should not be presented as suitable for clinical screening or as capable of directly supporting clinical decision-making. We have therefore revised the Discussion to explicitly acknowledge this limitation and to state that substantially more non-CKD samples are required before clinical deployment can be considered. We have also clarified that, despite the high sensitivity observed for CKD cases, the current framework is research-oriented and is not intended for standalone clinical deployment. |
| 38 | Issue 40: RQ3 "Few-Shot" Claim Unsupported "The findings demonstrate that HXAI-ML approach is powerful, flexible, and interpretational." Problem: The "few-shot" claim is unsupported because using 10% of data is not few-shot learning. Mandatory Action: Remove "few-shot" from the discussion and replace with "limited-sample domain adaptation." | **Thank you for this important clarification.**  We agree that describing the use of 10% of the target-domain dataset as “few-shot learning” was not methodologically appropriate and could overstate the nature of the adaptation setting. We have therefore removed the term “few-shot” throughout the manuscript and replaced it with “limited-sample domain adaptation” or “limited-sample target-domain adaptation,” as appropriate. We have also revised RQ3 and the corresponding Discussion statements to accurately describe the experimental setting without implying a formal few-shot learning paradigm. |
| 39 | B. Moderate Issues Limitations Section is Inadequate The limitations section only mentions: • Deep learning not used • Structured data only • Transformer models not compared Missing limitations: • Extremely small non-CKD sample (12 cases) • Low specificity making model clinically unusable • No external validation on a third dataset • Mathematical inconsistency between Table 3 and Fig. 5 Mandatory Action: Expand the limitations section to include all major methodological shortcomings. | **We thank the reviewer for this important observation.**  We agree that the previous limitations section did not sufficiently address the methodological and clinical constraints of the study. We have substantially revised Section 5.4, Limitations of the Proposed Work, to provide a more comprehensive and transparent discussion of the study limitations.  Specifically, the revised section now explicitly addresses: **(i)** the extremely small number of non-CKD cases in the target dataset (13 non-CKD versus 152 CKD cases in the 10% adaptation subset, and 122 non-CKD versus 1,372 CKD cases in the 90% blind holdout), **(ii)** the low specificity and resulting high false-positive rate of the adapted models, **(iii)** the consequent limitation that the current framework should not be considered suitable for standalone clinical screening, **(iv)** the absence of external validation using a third independent dataset, **(v)** the use of only 10% of the target-domain data for adaptation, and **(vi)** the absence of multimodal, deep-learning, and transformer-based comparisons.  In addition, the experimental analysis was rerun using the corrected and consistent target-domain adaptation pipeline. The revised manuscript now reports the corresponding performance metrics together with the confusion matrices and class-specific measures, reducing the possibility of inconsistencies between tabulated results and graphical representations. Rather than presenting the framework as clinically deployable, the revised manuscript explicitly states that substantially larger and clinically representative non-CKD samples, improved class-wise discrimination, calibration, and independent external validation are required before clinical deployment can be considered. |
| 40 | Future Work Lacks Specificity Future work suggests "implementing deep learning models" and "testing in more diverse datasets" — these are vague and generic. Mandatory Action: Provide specific, concrete future directions: • Use GANs to generate synthetic non-CKD samples. • Apply cost-sensitive learning instead of oversampling. • Test on a third independent dataset (e.g., NHANES CKD dataset). | **We thank the reviewer for this suggestion.**  We have revised the Future Work section to provide concrete and technically specific research directions. The revised manuscript now proposes **GAN-based generation of synthetic non-CKD samples, cost-sensitive learning as an alternative to oversampling, and validation using a third independent dataset such as NHANES**. We also explicitly identify improving specificity while maintaining CKD sensitivity as a key objective for future investigation. |
| 41 | SECTION 7: CONCLUSIONS AND REFERENCES A. Critical Issues Conclusions Overstate Findings The conclusion repeats the incorrect claims: • "91.83% accuracy" (should be 84.61% for GBC or clarify which dataset) • "100% recall" (false—highest is 99.84% for ETC) • "framework is effective and capable of assisting physicians" (unsupported given low specificity) Mandatory Action: Rewrite the conclusion to honestly reflect the model's limitations and clinical unsuitability. | **We thank the reviewer for identifying these inconsistencies**.  We have completely revised the Conclusions section to remove the previously reported **91.83% accuracy and 100% recall claims** and to ensure that the conclusions correspond to the corrected experimental results. The revised conclusion identifies **RO+TL+ETC as the best-performing model in the 10% target-adaptation experiment** and explicitly acknowledges the low specificity, high false-positive rate, limited number of non-CKD cases, and lack of external validation. We have also removed the unsupported claim that the framework is currently capable of assisting physicians in clinical decision-making and clarified that the framework is **not clinically deployable at this stage.** |
| 42 | Citation Errors Several references have formatting issues: • Line 1091: "Almasoud, M., & E, T." → Should have full author names. • Line 1195: "Rezk et al., 2025a, 2025b" → Check that both are correctly cited and that the "a" and "b" designations are correct. Mandatory Action: Review all references for completeness and correct formatting. | **Thank you for identifying these citation-formatting issues.**  We have carefully reviewed the reference list and in-text citations throughout the manuscript. The incomplete author information for **Almasoud et al. (2019)** has been corrected to provide the authors' complete names in accordance with the journal's reference style. We also checked the citations for **Rezk et al. (2025a, 2025b)** and verified that the a and b entries were found to point to the same article. We have merged them into a single reference, Rezk et al. (2025). The passage previously attributed to Rezk et al. (2025a), which describes an Extra Trees model with feature selection and SHAP interpretation, in fact refers to Elshewey et al. (2025), and the attribution has been corrected. In addition, the remaining references were reviewed for completeness, consistency, author names, publication year, journal information, and in-text citation matching. |
