# Supplementary material for: An explainable machine learning framework for cross-dataset chronic kidney disease risk prediction using hybrid data balancing
Source: PLoS One. 2026 Nov 5;21(11):e0362320. doi: 10.1371/journal.pone.0362320 (PMC13739112; doi:10.1371/journal.pone.0362320)
Supplement: Supplementary file 2 [file pone.0362320.s003.docx]

**RESPONSE TO REVIEWERS**

| **Manuscript Title** | An Explainable Machine Learning Framework for Cross-Dataset Chronic Kidney Disease Risk Prediction Using Hybrid Data Balancing |  |
| --- | --- | --- |
| **Journal** | Plos One |  |
| Reviewer #1 | Reviewer #1: The authors have addressed all substantive comments from the first round of review. The remaining issue (the definition of N in Equation (7)) is a minor typographical/notational correction that can be easily fixed. I recommend that the authors: 1. Correct the definition of N in Equation (7) to ‘N = number of samples’ (with l(sᵢ, śᵢ) = loss function), and 2. Conduct a final proofread of the entire manuscript to catch any remaining typographical errors. The manuscript should be accepted without further rounds of external review. | **Thank you for your thorough and constructive review. We are pleased that the substantive comments from the first round have been fully addressed.**  Regarding point 1: The definition of N in Equation (7) has been explicitly clarified in the manuscript. The notation now reads: N = number of samples, and l(sᵢ, śᵢ) = loss function, which appear in the explanatory text immediately following Equation (7).  Regarding point 2: We have conducted a thorough final proofread of the entire manuscript. All typographical errors identified during this review have been corrected. We are confident that the manuscript is now ready for publication. |

We sincerely thank the Editor for their thorough and constructive assessment of our manuscript. Their feedback has strengthened the positioning, methodological transparency, and presentation of the revised work.

Below is a detailed point-by-point response to each comment, organized by source.

# Response to Additional Editor

| **#** | **Reviewer Comment** | **Authors' Response and Location in Revised Manuscript** |
| --- | --- | --- |
| 1 | The revised manuscript exhibits significant improvement compared to the previous version. Specifically, the authors have rerun the analysis using a leakage-controlled pipeline, clarified the target-domain adaptation procedure, resolved inconsistencies in performance metrics and confusion matrices, expanded the reporting of class-specific performance measures, moderated clinical claims, enhanced reproducibility reporting, and revised the interpretation of the model’s relatively low specificity and discriminative performance. | **Thank you for the valuable feedback.**  We are grateful for the overall positive feedback for the updated analytical pipeline, consistency in performance measurements, expanded class-specific evaluation, increased reporting of reproducibility and the target domain adaptation procedure. We have also studied and tempered the clinical claims and explained the restrictions of low specificity and limited discriminative performance. These revisions help make the findings presented clearly and the structure of the framework more suitable to be called an exploratory cross-dataset machine-learning evaluation and not a clinically deployable machine-learning system. |
| 2 | The substantive concerns raised during the previous review have been addressed. The manuscript is now technically sound, statistically appropriate, adequately supported by the available data, and clearly written. The remaining issues are minor and should be readily addressed before the manuscript can proceed further. | **We thank the reviewer for this important observation**.  We appreciate the recognition that the substantive concerns raised during the previous review have been addressed and that the manuscript has improved in technical rigor, statistical analysis, evidential support, and clarity. We have carefully considered the remaining minor issues and will address them to further improve the manuscript before resubmission. |
| 3 | Equation (7) should be revised so that N is explicitly defined as the number of samples, and \(l(s_i,\hat{s}_i)\) is clearly defined as the loss function. | **Thank you for the valuable concern.**  We have revised the explanation accompanying Equation (7) to explicitly define (N) as the number of samples and clarify the meaning of the loss function, the true label (actual target value), the predicted label (model output) for the (i)-th training example, the model parameters, and the regularization term. These definitions have been added immediately below Equation (7) to improve mathematical clarity and reproducibility. |
| 4 | A thorough final proofread of the entire manuscript is recommended, including equations, mathematical notation, figure and table labels, abbreviations, references, typographical errors, and consistency of terminology. | **Thank you for this valuable recommendation.**  We have carefully proofread the revised manuscript to improve its overall accuracy, consistency, and readability. We reviewed the equations and mathematical notation, figure and table labels, abbreviations, references, and typographical errors. We also checked the consistency of terminology throughout the manuscript, particularly the terminology related to target-domain adaptation, class-specific performance metrics, and explainable AI. These revisions aim to improve the clarity, presentation, and reproducibility of the manuscript. |
| 5 | Given the extent of previous revisions and reanalysis, pay particular attention to ensuring that all numerical values in the Abstract, Results, tables, figures, Discussion, and Conclusion are fully consistent with the final analysis. | **Thank you for this important recommendation.**  We have carefully reviewed the numerical values throughout the revised manuscript to ensure consistency with the final analysis. In particular, we cross-checked the Abstract, Results, tables, figures, Discussion, and Conclusion, including the reported performance metrics, class distributions, and model comparisons. We also checked that the interpretation of the results accurately reflects the final findings, particularly the distinction between high sensitivity and limited specificity under class imbalance. Any discrepancies identified during this review have been corrected to ensure that the numerical results and their interpretation are consistent throughout the manuscript. |
