# Supplementary material for: An explainable machine learning framework for cross-dataset chronic kidney disease risk prediction using hybrid data balancing
Source: PLoS One. 2026 Nov 5;21(11):e0362320. doi: 10.1371/journal.pone.0362320 (PMC13739112; doi:10.1371/journal.pone.0362320)
Supplement: Peer review history

# Peer review: Decision Letter 0

**DOI:** 10.1371/journal.pone.0362320.r001

**Authors:** Jordan Llego

3 Aug 2026

Dear Dr. Tanvir Anzum,

Thank you for submitting your manuscript to PLOS ONE. After careful consideration, we feel that it has merit but does not fully meet PLOS ONE’s publication criteria as it currently stands. Therefore, we invite you to submit a revised version of the manuscript that addresses the points raised during the review process.

Please submit your revised manuscript by Oct 02 2026 11:59PM. If you will need more time than this to complete your revisions, please reply to this message or contact the journal office at plosone@plos.org. When you're ready to submit your revision, log on to https://www.editorialmanager.com/pone/ and select the 'Submissions Needing Revision' folder to locate your manuscript file.

- A letter that responds to each point raised by the academic editor and reviewer(s). You should upload this letter as a separate file labeled 'Response to Reviewers'.
- A marked-up copy of your manuscript that highlights changes made to the original version. You should upload this as a separate file labeled 'Revised Manuscript with Track Changes'.
- An unmarked version of your revised paper without tracked changes. You should upload this as a separate file labeled 'Manuscript'.

If you would like to make changes to your financial disclosure, please include your updated statement in your cover letter. Guidelines for resubmitting your figure files are available below the reviewer comments at the end of this letter.

If applicable, we recommend that you deposit your laboratory protocols in protocols.io to enhance the reproducibility of your results. Protocols.io assigns your protocol its own identifier (DOI) so that it can be cited independently in the future. For instructions see: https://journals.plos.org/plosone/s/submission-guidelines#loc-laboratory-protocols. Additionally, PLOS ONE offers an option for publishing peer-reviewed Lab Protocol articles, which describe protocols hosted on protocols.io. Read more information on sharing protocols at https://plos.org/protocols?utm_medium=editorial-email&utm_source=authorletters&utm_campaign=protocols.

As the corresponding author, your ORCID iD is verified in the submission system and will appear in the published article. PLOS supports the use of ORCID, and we encourage all coauthors to register for an ORCID iD and use it as well. Please encourage your coauthors to verify their ORCID iD within the submission system before final acceptance, as unverified ORCID iDs will not appear in the published article. *Only*  the individual author can complete the verification step; PLOS staff *cannot*  verify ORCID iDs on behalf of authors.

We look forward to receiving your revised manuscript.

Kind regards,

Jordan Llego, PhD ELM, D. Hon. Ex., PhDN, RN

Academic Editor

PLOS One

Journal Requirements:

When submitting your revision, we need you to address these additional requirements.

1. Please ensure that your manuscript meets PLOS ONE's style requirements, including those for file naming. The PLOS ONE style templates can be found at

https://journals.plos.org/plosone/s/file?id=wjVg/PLOSOne_formatting_sample_main_body.pdf and

https://journals.plos.org/plosone/s/file?id=ba62/PLOSOne_formatting_sample_title_authors_affiliations.pdf

2. Please note that PLOS One has specific guidelines on code sharing for submissions in which author-generated code underpins the findings in the manuscript. In these cases, we expect all author-generated code to be made available without restrictions upon publication of the work. Please review our guidelines at https://journals.plos.org/plosone/s/materials-and-software-sharing#loc-sharing-code and ensure that your code is shared in a way that follows best practice and facilitates reproducibility and reuse.

3. Please remove your figures from within your manuscript file, leaving only the individual TIFF/EPS image files, uploaded separately. These will be automatically included in the reviewers’ PDF.

4. We note you have included a table to which you do not refer in the text of your manuscript. Please ensure that you refer to Table 1 in your text; if accepted, production will need this reference to link the reader to the Table.

5. If the reviewer comments include a recommendation to cite specific previously published works, please review and evaluate these publications to determine whether they are relevant and should be cited. There is no requirement to cite these works unless the editor has indicated otherwise.

Additional Editor Comments:

Thank you for submitting your manuscript to PLOS ONE. Your study tackles an important clinical issue and explores class-balancing, cross-dataset model adaptation, and explainable AI for predicting chronic kidney disease risk. Using public datasets and several interpretability methods could make a valuable contribution. However, after reviewing your manuscript and the detailed reviewer comments, I have decided that the paper does not yet meet the journal’s standards for technical quality, analytical rigor, reproducibility, clear reporting, and proper interpretation of results. I am inviting you to submit a thoroughly revised version. Please note that this is a request for major methodological and analytical changes, not just editorial corrections.

The main concern is the inconsistency between the performance results shown in the abstract, tables, main text, and the confusion matrices in Figure 5. The manuscript states that the Random Oversampling with Tomek Links and Gradient Boosting setup achieved 91.83% accuracy and 100% recall. But the confusion matrix shows about 84.61% accuracy and 89.88% recall. These differences are significant because they affect your main claim about the best-performing model. The manuscript often highlights the RO+TL+GBC setup as the top performer, but the confusion matrix suggests another model may do better on some metrics.

You will need to rerun and independently verify the entire analysis. All numbers in the abstract, main text, tables, figures, supplementary materials, and conclusion must come from the same final analysis. The revised manuscript should clearly explain how you calculated accuracy, sensitivity, specificity, precision, recall, F1-score, ROC-AUC, and any other performance measures. Make sure the positive and negative classes are defined the same way throughout. In your response letter, include a clear record showing how you generated the corrected values for each table and figure.

The current analysis focuses too much on accuracy, recall, and F1-score, and does not give enough information about how the models perform on the minority non-CKD class. Because the classes are very imbalanced, the revised manuscript should also report specificity, sensitivity, positive and negative predictive values, balanced accuracy, Matthews correlation coefficient, and class-specific results where needed. Please include confidence intervals for the main performance estimates. The reviewer’s review of Figure 5 suggests that specificity may be very low for the models tested, meaning many non-CKD cases were classified as CKD. This is a major issue and directly affects how the results should be interpreted in a clinical context.

You will need to reconsider the clinical claims. The reported ROC-AUC values of about 0.60 show limited ability to distinguish between cases. Combined with the poor classification of the non-CKD group, these results do not currently support claims that the framework is ready for clinical screening, decision support, or real-world use. The revised manuscript should clearly separate methodological feasibility, predictive performance, cross-dataset adaptation, and clinical usefulness. Unless your new analyses are much stronger and well validated, the study should be described as an exploratory cross-dataset machine learning evaluation. Please remove or moderate any statements suggesting clinical readiness, strong generalizability, or improved patient-level prediction.

The way the manuscript describes the approach as “few-shot learning” should also be reconsidered. Using about 10% of the target dataset for calibration is better described as limited-data target calibration, transfer learning, domain adaptation, or target-domain adaptation. Please provide a clear and technically accurate definition of the learning method, support it with relevant methodological literature, and update the title, abstract, research questions, objectives, methods, results, discussion, and conclusion as needed. The terminology should match what was actually done and should not exaggerate the novelty of the framework.

The Methods section needs to be expanded to show analytical rigor and to rule out data leakage. The revised manuscript should report the exact source and target sample sizes, class distributions, label definitions, feature-matching steps, data-partitioning strategy, random seeds, preprocessing steps, imputation methods, scaling, balancing, calibration, model development, hyperparameter tuning, and final blind-test evaluation. Make it clear that imputation values, scaling, feature selection, balancing, and tuning were done only using the training or calibration data. No information from the blind test set should have been used in model development, selection, preprocessing, or hyperparameter tuning.

Please explain whether you selected models using cross-validation and whether the blind test set was only used after the full analysis pipeline was finished. Since you compared several classifiers and balancing methods, describe how you addressed the risk of optimistic model-selection bias. Using just one train-test split is usually not enough to show stable performance, especially with a small minority class. The revised analysis should use a suitable resampling method, like repeated stratified cross-validation, bootstrap evaluation, or another justified approach, and should report the distributions or confidence intervals for the main performance metrics.

The current manuscript does not provide enough information to reproduce the experiments. The revised version should include the full search spaces for hyperparameter tuning, the optimization strategy, the final hyperparameters for each model, the software packages and versions, computational environment, hardware details, and random-state settings. Ideally, the analysis code should be placed in a stable public repository. This repository should have scripts for data preprocessing, dataset splitting, balancing, model fitting, hyperparameter tuning, metric calculation, making tables and figures, and explainability analyses. Even though the original datasets are public, the Data Availability Statement should clearly separate the original data, processed data, derived outputs, and the code needed to reproduce the results.

Do not base claims that one model or balancing method is better than another only on small differences in single point estimates. Check if the performance differences stay consistent across repeated samples and random seeds. If you make formal comparisons, use a statistical method that fits paired model-evaluation data and describe it fully. The revised discussion should recognize uncertainty and avoid saying one model is clearly better if their confidence intervals or resampled performance results overlap a lot.

The explainability analyses using SHAP, LIME, and permutation importance need better methodological reporting and more careful interpretation. Just because the identified variables seem clinically reasonable does not mean the methods are clinically valid. The revised manuscript should state which final model and dataset were used for each explanation, how you chose the reference or background samples, whether the explanations were based on calibration or blind-test data, and whether feature rankings stayed stable across resamples. The discussion should not treat agreement with known clinical knowledge as proof of causality, external validity, or readiness for clinical use.

All equations and technical expressions should be checked independently. The reviewer found issues with the notation in the XGBoost objective function and how its terms are defined. Please rewrite the equations using correct and consistent notation, and clearly define every symbol. Fix any non-standard or incorrect terms, such as using “SM” instead of “SMOTE,” “roc-auci,” and “over sounding,” throughout the text, tables, and figures.

The manuscript needs a thorough revision for language, structure, and scientific clarity. Many sentences are grammatically incorrect, repetitive, unclear, or hard to understand. The Introduction and Literature Review should be shorter and focus on the specific methodological gaps your study addresses. Remove repeated general information about chronic kidney disease, delete duplicate sections, and make sure the problem statement clearly covers class imbalance, cross-dataset adaptation, interpretability needs, and dataset limitations. Check all in-text citations against the reference list, fix any incomplete or incorrect references, and include key methodological literature where relevant.

Rewrite the abstract and conclusion only after you have verified the complete analysis. The new abstract should include the corrected performance values and report specificity or another suitable measure for non-CKD classification. The conclusion should clearly state the strengths and limitations of the framework and should not claim the model is clinically effective if it still does not perform well on the minority class. Even limited or negative results can be valuable, but they must be reported clearly and without unsupported claims.

When you submit the revised manuscript, include a detailed point-by-point response to every reviewer and editorial comment. The response should show exactly where each change was made and explain any comments you did not follow. Please also submit a marked version with all changes, a clean version, corrected tables and figures, updated supplementary materials, and full data and code availability details. The response letter should briefly explain the reanalysis and confirm that all reported numbers have been independently checked.

Please note that being invited to revise does not guarantee acceptance. Because the numerical, methodological, and interpretive issues are significant, the revised manuscript will likely need more expert review. If you do not resolve the conflicting performance results, show a leakage-free and reproducible analysis, or update the clinical claims based on the evidence, the manuscript may be rejected.

We appreciate your efforts on this study and hope these comments help you create a more technically sound, reproducible, and clearly interpreted manuscript.

[Note: HTML markup is below. Please do not edit.]

Reviewers' comments:

Reviewer's Responses to Questions

**Comments to the Author**

1. Is the manuscript technically sound, and do the data support the conclusions?

Reviewer #1: Partly

**********

2. Has the statistical analysis been performed appropriately and rigorously?

Reviewer #1: No

**********

3. Have the authors made all data underlying the findings in their manuscript fully available?

The PLOS Data policy

Reviewer #1: Yes

**********

4. Is the manuscript presented in an intelligible fashion and written in standard English?

Reviewer #1: No

**********

Reviewer #1: OVERVIEW

This manuscript addresses a genuinely critical public health problem—chronic kidney disease (CKD) affects over 850 million people globally and is a leading cause of mortality. The application of machine learning for early detection is both timely and clinically relevant. The authors attempt to tackle three significant challenges: severe class imbalance, cross-dataset generalizability, and model interpretability through XAI techniques (SHAP, LIME).

However, despite the compelling motivation, the manuscript suffers from fundamental methodological flaws that undermine its scientific validity:

1. Mathematical Contradiction: The performance metrics reported in Tables 3 and 5 (91.83% accuracy, 100% recall for GBC) are inconsistent with the confusion matrices shown in Fig. 5. The GBC confusion matrix shows TP=1110, FN=125, FP=82, TN=28, yielding 84.61% accuracy and 89.88% recall not 91.83% and 100%.

2. Lines 34-36: The abstract reports accuracy, precision, recall, F1, and ROC-AUC—but does not report specificity.

Add specificity to the abstract: "Specificity ranged from 0.91% (ETC) to 27.27% (DTC), indicating that 73-99% of healthy patients would be misclassified as having CKD."

3. Misleading Terminology: The claim of "few-shot learning" (Lines 30-31, 129) is inappropriate. Using 10% (~166 samples) of the target dataset for calibration is standard transfer learning, not few-shot learning (which typically uses 1-5 examples per class).

4. ROC-AUC Interpretation is Inadequate

Line 35: "62.05% ROC-AUC" is reported without context.

Problem: A ROC-AUC of 62.05% is barely above random guessing (50%) and indicates poor discriminative ability.

• Revise to: "The ROC-AUC values ranged from 59.77% to 62.05%, confirming limited discriminative ability across probability thresholds."

5. Clinical Non-Viability: All models exhibit critically low specificity (0.91% to 27.27%), meaning 73-99% of healthy patients would be misclassified as having CKD. This renders the models unsuitable for clinical screening.

Based on the confusion matrices in Fig. 5 (Page 26), all models exhibit critically low specificity ranging from 0.91% to 27.27%. This means 73-99% of healthy patients would be misclassified as having CKD, rendering the models unsuitable for clinical screening.

Extracting the True Negatives (TN) and False Positives (FP) from each confusion matrix in Fig. 5:

Model TN FP Specificity = TN/(TN+FP) Healthy Patients Misclassified

DTC 30 80 30/110 = 27.27% 72.73%

ETC 1 109 1/110 = 0.91% 99.09%

RFC 12 98 12/110 = 10.91% 89.09%

GBC 28 82 28/110 = 25.45% 74.55%

Clinical Interpretation: A screening tool that misclassifies 73-99% of healthy patients would cause unnecessary patient anxiety, invasive follow-up procedures, and healthcare resource waste. Clinical screening standards require specificity >90% for deployment.

Authors' Own Admissions: At Lines 660-672, the authors state models are "not capable of repeatedly distinguishing between CKD and not-CKD groups." At Lines 681-684, they acknowledge models "will bias towards predicting CKD... lowers the capability to predict healthy cases correctly."

Conclusion: Based on the authors' own confusion matrices (Fig. 5) and their own admissions (Lines 660-672, 681-684), the models are clinically unusable. This is a mathematical conclusion from the data presented, not a subjective critique.

6. Incomplete Reporting: No hyperparameter values, hardware specifications, cross-validation strategies, or statistical significance tests are provided, violating PLOS One's reproducibility requirements.

7. Citation Error: Reference "Almasoud & E, 2019" (Line 1091) is incorrectly formatted—the second author's initials should be expanded or the citation should follow journal style.

Introduction

Grammatical Errors

Lines 44-46: "chronic kidney disease is experienced by more than 850 million patients all over the world. Approximately one point five million humans lose their lives to CKD annually."

Problem: "one point five million" should be "1.5 million." "humans" is awkward—use "people" or "patients."

Line 52-53: "The sixth cause of death with the highest growth is the globally widespread kidney disease" — awkward phrasing.

Line 58: "exposure to harmful environmental phenomena" — "phenomena" is plural but used as singular.

"Over sounding" (Line 114)

Line 114: "Over sounding and Intersecting Samples" — This should be "Oversampling and Intersecting Samples" or "Overlapping and Intersecting Samples."

Problem Statement Lacks Specificity

Lines 117-126: The problem statement is vague and generic. It does not specifically address the CKD datasets, the severity of imbalance (137 CKD vs. 12 non-CKD), or the technical challenges identified in Section 1.1.

Mandatory Action: Rewrite the problem statement to directly reference the specific challenges identified (class imbalance, cross-dataset adaptation, interpretability) and the dataset limitations.

Minor Issues

Citation Order

Line 44: References Ortiz et al., 2026a and 2026b are used. These should be checked to ensure the "a" and "b" designations are correct and consistent with the reference list (Lines 1179-1184).

Missing Citation

Line 127: RQ1 references "balancing technique" but the foundational SMOTE paper (Chawla et al., 2002, Journal of Artificial Intelligence Research, 16:321-357) is absent from the reference list. This is a mandatory citation for any work using SMOTE.

Mandatory Action: Add Chawla et al. (2002) to the reference list and cite it appropriately.

LITERATURE REVIEW

Critical Issues

Citation Error (Almasoud & E, 2019)

Line 1091: Reference appears as "Almasoud, M., & E, T. (2019)." This is incorrectly formatted. The second author should have a full name, not just an initial.

Issue 14: Duplicate Text

Lines 240-246: "XAI allows scientists and physicians to understand how a machine-learning model arrives at a guess..." appears twice (Lines 240-243 and 243-246) with slight variations. This is clearly a copy-paste error.

Mandatory Action: Remove the duplicate paragraph.

B. Moderate Issues

Redundant Literature Review

Lines 180-205 (Machine Learning in CKD Prediction) and Lines 213-253 (Explainable AI in CKD Risk Prediction) contain redundant descriptions of CKD as a disease. The introduction already covers this.

Mandatory Action: Remove redundant disease descriptions and focus on the machine learning aspects.

Missing Recent Citations

The literature review cites mostly papers from 2019-2024, but several recent CKD prediction papers from 2025-2026 are relevant:

• Elshewey et al. (2025) is cited but not discussed in detail.

• Gupta et al. (2026) is cited but should be compared more directly.

• Rezk et al. (2025a, 2025b) are used but could be expanded.

Mandatory Action: Expand the discussion of recent work (2025-2026) and provide more direct comparisons with the authors' methodology.

Citation Formatting

Several citations in the text are not consistently formatted:

• "Khandoker et al. (2024)" (Line 217) is cited but Khandoker is not the first author of Ghosh & Khandoker (2024)—this appears to be an error.

Mandatory Action: Check all in-text citations against the reference list and correct author names.

METHODOLOGY

Issue 20: Equation (7) Contains Errors

Lines 416-421: The XGBoost regularization objective:

text

L(θ) = Σᵢ₌₁ⁿ l(sᵢ, ŝᵢ) + λ Σⱼ₌₁ᵏ ωⱼ² (7)

where n = loss function

Problems:

• n is used as the loss function, but it was previously used for the number of input features.

• The loss function should be denoted as l(yᵢ, ŷᵢ) or l(sᵢ, ŝᵢ).

• The regularization term should use Ω(fₖ) or a similar expression.

Mandatory Action: Correct Equation (7) with proper notation and define all terms clearly.

"roc-auci" Typo (Line 441)

Line 441: "roc-auci" should be "ROC-AUC".

"SM" vs. "SMOTE" Inconsistency

The manuscript uses "SM" as an abbreviation for SMOTE throughout (Line 320). This is non-standard and confusing. The accepted abbreviation is "SMOTE."

Mandatory Action: Replace all instances of "SM" with "SMOTE" in text, tables, and figures.

Missing Hyperparameter Details

The manuscript mentions "parameter tuning" but provides no specifics:

• Search space (e.g., n_estimators: [50, 100, 200])

• Optimization strategy (GridSearchCV, RandomSearchCV)

• Final optimal hyperparameters

• Random seeds used

Mandatory Action: Add a supplementary table listing all final hyperparameters for all six models.

Moderate Issues

Issue 25: Data Leakage Risk

"we trained the imputation model only on the training dataset, then applied it to the test dataset" — This is good practice. However, the authors do not specify:

• Whether the StandardScaler was fitted only on the training/calibration set.

• Whether the 10% calibration set was used for scaling or the full Kaggle dataset.

Mandatory Action: Clarify explicitly that all preprocessing parameters (imputation values, standard scaler means/variances) were computed exclusively on the 10% calibration set.

Issue 27: Hyperparameter Tuning Not Described

The authors mention "grid search" in the literature review but do not describe its application in their methodology.

Mandatory Action: Add a subsection describing the hyperparameter tuning process, including the search strategy, range of values, and final selected parameters.

RESULTS

Critical Issues

Table 3 Metrics Are Inconsistent with Fig. 5

Pages 25-26 vs. Fig. 5: The reported 91.83% accuracy and 100% recall for GBC (RO+TL) do not match Fig. 5 (84.61% accuracy, 89.88% recall). This is a fatal inconsistency.

Mandatory Action:

• Either correct Table 3 to reflect the confusion matrix values (84.61% accuracy, 89.88% recall).

• Or replace Fig. 5 with the correct confusion matrix that matches Table 3.

• This must be resolved before resubmission.

GBC is Not the Best Model Based on Fig. 5

The manuscript repeatedly claims GBC achieved the "best overall performance." However, based on Fig. 5:

Model Accuracy Precision Recall F1-Score

ETC 91.75% 91.88% 99.84% 95.69%

GBC 84.61% 93.12% 89.88% 91.48%

ETC outperforms GBC on accuracy, recall, and F1-score.

Mandatory Action:

• Correct the "best model" claim throughout the manuscript.

• If GBC is truly best, explain why Fig. 5 contradicts this.

100% Recall Claim is False

The manuscript repeatedly claims 100% recall.

Problem: Based on Fig. 5, the highest recall is 99.84% (ETC), and GBC is only 89.88%. No model achieves 100% recall.

Mandatory Action: Remove all 100% recall claims and replace with actual values.

Issue 32: Specificity is Omitted

Table 3 and Table 5: No specificity values are reported.

Problem: Based on Fig. 5:

• DTC specificity = 27.27% (30/110)

• ETC specificity = 0.91% (1/110)

• RFC specificity = 10.91% (12/110)

• GBC specificity = 25.45% (28/110)

Mandatory Action: Add Specificity as a column in Tables 3 and 5 and discuss its clinical implications.

Key Observation 1 is Incorrect

Line 686: "The best overall performance was achieved with RO+TL+GBC. It gives 91.83% accuracy, 100% recall, and the highest ROC-AUC of 62.05%."

Problem: This statement is contradicted by Fig. 5 (84.61% accuracy, 89.88% recall). The highest ROC-AUC is 62.05% for GBC, but that does not make it "best overall."

Mandatory Action: Correct this observation to reflect the actual data and acknowledge that ETC achieves higher accuracy and recall.

Moderate Issues

No Statistical Significance Testing

Apply McNemar's test for pairwise classifier comparisons and report p-values. If differences are not statistically significant (p > 0.05), state that no single classifier is superior.

Hardware Specifications Missing (Table 6)

Table 6 reports training and prediction times without CPU model, RAM, OS, or library versions.

Mandatory Action: Add a "Computational Environment" subsection detailing: CPU model, RAM, OS, Python version, and key library versions.

SHAP Figure Labels

Fig. 7, 8A, 8B: The SHAP plots show feature names like "fastingbloodsugar" and "diastolicbp." The text uses "fasting blood sugar" and "diastolic blood pressure."

Mandatory Action: Ensure consistent naming between figures and text. Use spaces or underscores consistently. Consider using standard abbreviations (FBS, DBP) throughout.

SECTION 6: DISCUSSION

A. Critical Issues

Discussion Contradicts Results

"The performance of Gradient Boosting was the best. Random Oversampling and Tomek Links achieved the best results."

Problem: This contradicts Fig. 5, where ETC outperforms GBC on accuracy, recall, and F1-score.

Mandatory Action: Correct the discussion to reflect the actual results.

Clinical Applicability Claims are Overstated

"Hence, the proposal system is correct and understandable. It has the capability of assisting actual clinical decision-making."

Problem: With specificity as low as 0.91%, the model misclassifies 99% of healthy patients (ETC). This is not clinically acceptable.

Mandatory Action: Revise to: "The low specificity across all models (0.91-27.27%) indicates that the current framework is unsuitable for clinical screening. Substantially more non-CKD samples are required before deployment can be considered."

Issue 40: RQ3 "Few-Shot" Claim Unsupported

"The findings demonstrate that HXAI-ML approach is powerful, flexible, and interpretational."

Problem: The "few-shot" claim is unsupported because using 10% of data is not few-shot learning.

Mandatory Action: Remove "few-shot" from the discussion and replace with "limited-sample domain adaptation."

B. Moderate Issues

Limitations Section is Inadequate

The limitations section only mentions:

• Deep learning not used

• Structured data only

• Transformer models not compared

Missing limitations:

• Extremely small non-CKD sample (12 cases)

• Low specificity making model clinically unusable

• No external validation on a third dataset

• Mathematical inconsistency between Table 3 and Fig. 5

Mandatory Action: Expand the limitations section to include all major methodological shortcomings.

Future Work Lacks Specificity

Future work suggests "implementing deep learning models" and "testing in more diverse datasets" — these are vague and generic.

Mandatory Action: Provide specific, concrete future directions:

• Use GANs to generate synthetic non-CKD samples.

• Apply cost-sensitive learning instead of oversampling.

• Test on a third independent dataset (e.g., NHANES CKD dataset).

SECTION 7: CONCLUSIONS AND REFERENCES

A. Critical Issues

Conclusions Overstate Findings

The conclusion repeats the incorrect claims:

• "91.83% accuracy" (should be 84.61% for GBC or clarify which dataset)

• "100% recall" (false—highest is 99.84% for ETC)

• "framework is effective and capable of assisting physicians" (unsupported given low specificity)

Mandatory Action: Rewrite the conclusion to honestly reflect the model's limitations and clinical unsuitability.

Citation Errors

Several references have formatting issues:

• Line 1091: "Almasoud, M., & E, T." → Should have full author names.

• Line 1195: "Rezk et al., 2025a, 2025b" → Check that both are correctly cited and that the "a" and "b" designations are correct.

Mandatory Action: Review all references for completeness and correct formatting.

Recommendation: Major Revision Required.

**********

what does this mean?). If published, this will include your full peer review and any attached files.

If you choose “no”, your identity will remain anonymous but your review may still be made public.

**Do you want your identity to be public for this peer review?**  For information about this choice, including consent withdrawal, please see our Privacy Policy

Reviewer #1: No

**********

[NOTE: If reviewer comments were submitted as an attachment file, they will be attached to this email and accessible via the submission site. Please log into your account, locate the manuscript record, and check for the action link "View Attachments". If this link does not appear, there are no attachment files.]

To ensure your figures meet our technical requirements, please review our figure guidelines: https://journals.plos.org/plosone/s/figures

You may also use PLOS’s free figure tool, NAAS, to help you prepare publication quality figures: https://journals.plos.org/plosone/s/figures#loc-tools-for-figure-preparation.

NAAS will assess whether your figures meet our technical requirements by comparing each figure against our figure specifications.

# Author comment: Author response to Decision Letter 1

**DOI:** 10.1371/journal.pone.0362320.r002

15 Sep 2026

RESPONSE TO REVIEWERS

Manuscript title: An Explainable Machine Learning Framework for Cross-Dataset Chronic Kidney Disease Risk Prediction Using Hybrid Data Balancing

Journal: PLOS ONE

We sincerely thank the Editor and Reviewer #1 for the thorough and constructive assessment of our manuscript. The feedback has strengthened the positioning, the methodological transparency and the presentation of the revised work. Below is a point-by-point response to every comment, organised by source.

SUMMARY OF THE REANALYSIS

The complete experiment was rerun using a corrected and leakage-controlled pipeline. The source-domain models were selected by stratified five-fold cross-validation with randomized hyperparameter search. The target dataset was then split into a 10% adaptation and calibration subset and a 90% blind holdout subset. The blind holdout was used only for the final evaluation. Every value reported in the abstract, main text, tables, figures, supplementary materials and conclusion was regenerated from this single final analysis. All reported numbers were independently checked. The TP, TN, FP and FN counts were extracted from the final blind-holdout predictions. Accuracy, sensitivity, specificity, precision, F1-score and balanced accuracy were recalculated from those counts and cross-checked against the tables and the confusion matrices in Fig. 5. Under the corrected pipeline the RO+TL+ETC configuration replaces RO+TL+GBC as the best-performing configuration.

PART A. RESPONSE TO THE ADDITIONAL EDITOR

Comment A1. The manuscript does not yet meet the journal's standards for technical quality, analytical rigor, reproducibility, clear reporting and proper interpretation of results. This is a request for major methodological and analytical changes, not editorial corrections.

Response. We sincerely thank the Editor for the careful assessment and for the opportunity to submit a substantially revised version. We have conducted a comprehensive re-analysis using a revised and carefully controlled target-domain adaptation pipeline. The experimental results were regenerated using the 10% target-domain adaptation setting, with the remaining data retained as a blind holdout evaluation set. The revised analysis identifies RO+TL+ETC (Random Oversampling + Tomek Links + Extra Trees) as the best-performing configuration, rather than the previously reported RO+TL+GBC configuration. We have also made the following substantive changes.

1. Revised the performance analysis so that the reported accuracy, recall, F1-score, specificity, ROC-AUC and confusion matrices are mathematically consistent.

2. Removed the unsupported 100% recall and few-shot-learning claims and replaced them with the actual results and the more appropriate term "limited-sample domain adaptation".

3. Reported specificity explicitly and acknowledged the substantial difficulty of identifying non-CKD cases.

4. Revised the clinical interpretation to make clear that the current models should not be considered clinically deployable screening systems because of their low specificity.

5. Added statistical significance analysis, including pairwise McNemar tests with Holm adjustment.

6. Expanded the methodological description of hyperparameter tuning, including the search strategy, parameter ranges, selected parameters and random seeds.

7. Added computational-environment information, including hardware, operating system, Python version and library versions.

8. Clarified the preprocessing and adaptation procedure, including the distinction between preprocessing performed before adaptation and the subsequent target-domain adaptation process.

9. Corrected the XGBoost objective-function notation and clarified the definitions of all mathematical terms.

10. Revised the SHAP analysis and feature labels to ensure consistent terminology between the figures and the manuscript.

11. Expanded the limitations section to acknowledge the very small number of non-CKD cases, the low specificity, the absence of independent external validation and other methodological limitations.

12. Revised the discussion and conclusion so that they accurately reflect the observed results and do not overstate clinical applicability.

13. Expanded the future-work section with specific directions, including synthetic generation of minority-class cases, cost-sensitive learning and validation using an independent dataset such as NHANES.

14. Corrected and audited the references, including the Almasoud et al. citation, the Rezk et al. year designations and the addition of the foundational SMOTE reference (Chawla et al., 2002).

These changes represent a substantial methodological and analytical revision rather than an editorial modification. We believe the revised manuscript provides a more transparent, reproducible and appropriately cautious assessment of cross-dataset CKD prediction under limited target-domain adaptation.

Comment A2. The main concern is the inconsistency between the performance results shown in the abstract, tables, main text and the confusion matrices in Figure 5.

Response. We thank the Editor for identifying this inconsistency. We agree that the previously reported values for the Gradient Boosting Classifier were not consistent with the confusion matrix. This indicates an error in the earlier result-generation pipeline. We reran the complete 10% target-domain adaptation experiment using the corrected and leakage-controlled pipeline. The target dataset is now divided into 10% adaptation and calibration data and 90% completely blind holdout data. Threshold selection was performed using the adaptation and calibration set only. The final performance metrics and confusion matrices are now generated from the same holdout predictions, which ensures mathematical consistency among Tables 3, 5 and Fig. 5. Under the corrected pipeline the RO+TL+ETC configuration is the best-performing model based on the reported accuracy criterion. On the 1,494-sample blind target holdout, RO+TL+ETC achieved 91.97% accuracy, 92.24% precision, 99.64% recall, 95.80% F1-score and 67.52% ROC-AUC. Its confusion matrix contains TN = 7, FP = 115, FN = 5 and TP = 1,367. These counts reproduce the reported accuracy and recall exactly. For GBC the corrected RO+TL+GBC result is 82.20% accuracy and 88.05% CKD recall, with the corresponding confusion matrix derived from the same holdout predictions. The previous statement identifying GBC as the best model has been removed. The revised analysis distinguishes between the highest raw accuracy (RO+TL+ETC) and more balanced minority-class behaviour (RO+TL+MLP), rather than treating GBC as the overall champion.

Comment A3. You will need to rerun and independently verify the entire analysis. All numbers must come from the same final analysis. Explain how each measure was calculated and keep the class definitions consistent. Include a clear record of how the corrected values were generated.

Response. We agree that all reported results must originate from one consistent final analysis. We reran and independently checked the complete experimental analysis using the finalised 10% target-domain adaptation pipeline. All numerical results in the abstract, main text, tables, figures, supplementary materials and conclusion were regenerated from this final analysis. The class definitions were kept consistent throughout. CKD was defined as the positive class (1) and non-CKD as the negative class (0). The 10% target-domain adaptation data were used for model adaptation and calibration. The blind holdout set was used for the final performance evaluation. Thresholds were selected using the calibration procedure and were not optimised using the blind holdout labels. The performance measures were calculated as follows.

Accuracy = (TP + TN) / (TP + TN + FP + FN)

Precision (PPV) = TP / (TP + FP)

Recall or sensitivity = TP / (TP + FN)

Specificity = TN / (TN + FP)

F1-score = 2 x (Precision x Recall) / (Precision + Recall)

Balanced accuracy = (Sensitivity + Specificity) / 2

ROC-AUC = area under the ROC curve calculated from the continuous predicted probabilities rather than from thresholded predictions.

MAE, MSE and RMSE were calculated from the binary observed and predicted class labels according to the definitions in the revised methodology.

We also independently checked the reported values against the corresponding confusion matrices. TP, TN, FP and FN were extracted directly from the final predictions. Accuracy, sensitivity, specificity, precision, F1-score and balanced accuracy were then recalculated from these counts. This verification eliminated the inconsistencies between the tables and the figures identified in the previous version. The revised analysis identifies RO+TL+ETC as the best-performing configuration according to the predefined selection criterion. The revised manuscript no longer interprets the high recall as evidence of clinical suitability, because the corresponding specificity remains low.

Comment A4. The analysis focuses too much on accuracy, recall and F1-score. Report specificity, sensitivity, PPV, NPV, balanced accuracy, MCC and class-specific results, and include confidence intervals.

Response. We agree that accuracy, sensitivity and F1-score alone do not adequately characterise performance under the substantial class imbalance in the target dataset. We performed an additional class-imbalance-aware evaluation of the six RO+TL classifiers on the final blind holdout set. The blind test set contains 1,494 observations, of which 1,372 are CKD and 122 are non-CKD. In addition to accuracy, sensitivity, precision and F1-score, we now report specificity, NPV, balanced accuracy, MCC and the underlying TN, FP, FN and TP counts. We also performed 2,000 non-parametric bootstrap resamples and report 95% confidence intervals for the principal performance measures. The results confirm that the models achieved relatively high sensitivity but had limited ability to identify non-CKD cases. ETC achieved the highest accuracy (91.97%, 95% CI 90.50 to 93.37%) and sensitivity (99.64%, 95% CI 99.27 to 99.93%), but its specificity was only 5.74% (95% CI 1.85 to 10.16%), with 115 of 122 non-CKD cases incorrectly classified as CKD. MLP achieved the highest specificity (25.41%, 95% CI 17.74 to 33.59%) and balanced accuracy (59.39%, 95% CI 55.51 to 63.40%), although these values also remain limited. We have revised the interpretation of the results and explicitly acknowledge that the low specificity substantially limits clinical applicability. The revised manuscript presents the framework as an exploratory cross-dataset machine-learning evaluation rather than a clinically deployable screening system.

Comment A5. Reconsider the clinical claims. The ROC-AUC values of about 0.60 and the poor classification of the non-CKD group do not support claims of clinical readiness. Separate methodological feasibility, predictive performance, cross-dataset adaptation and clinical usefulness.

Response. We agree that the original manuscript overstated the clinical implications of the framework. We have reconsidered and moderated these claims throughout the Abstract, Results, Discussion, Limitations and Conclusions. The revised manuscript now clearly distinguishes methodological feasibility, predictive performance, cross-dataset target-domain adaptation and clinical usefulness. The results show that the approach can be evaluated for cross-dataset adaptation using a limited target-domain calibration sample. However, the observed ROC-AUC values range from 0.56 to 0.68 across the RO+TL classifiers, and the specificity for the non-CKD class is substantially low. Both indicate limited discriminative ability. The RO+TL+ETC configuration achieves high sensitivity for CKD but only 5.74% specificity, which demonstrates a strong tendency to classify observations as CKD. High accuracy or sensitivity is therefore not interpreted as evidence of clinically useful or balanced prediction. We have removed or moderated all statements suggesting readiness for clinical screening, clinical decision support, patient-level deployment or real-world clinical use. We also avoid claims of strong generalizability, because the evaluation uses two public datasets and does not include validation on a third independent clinical dataset. The revised manuscript presents the work as an exploratory cross-dataset machine-learning evaluation of class-balancing strategies, limited-sample target-domain adaptation and explainable AI. We state explicitly that additional validation with larger and clinically representative datasets, particularly with sufficient non-CKD cases, is required before clinical usefulness can be assessed.

Comment A6. The description of the approach as few-shot learning should be reconsidered. Provide a technically accurate definition of the learning method and update the title, abstract, research questions, objectives, methods, results, discussion and conclusion.

Response. We agree that "few-shot learning" did not accurately describe our methodology. We have replaced it throughout the manuscript with "limited-sample target-domain adaptation". In our framework the models are trained on the source dataset, 10% of the target dataset is used for calibration and adaptation, and the remaining 90% is retained as a blind holdout set. We have updated the title, abstract, research questions, objectives, methodology, results, discussion and conclusion accordingly, and have added relevant transfer-learning and domain-adaptation references.

Comment A7. Expand the Methods to show analytical rigor and to rule out data leakage. Report the sample sizes, class distributions, label definitions, feature matching, partitioning, seeds, preprocessing, imputation, scaling, balancing, calibration, model development, tuning and blind-test evaluation.

Response. We have expanded the Methods section to describe the complete analytical pipeline and the data-leakage controls. The revised manuscript reports the source and target datasets, the class definitions, the common feature set, the preprocessing and imputation procedures, the target-domain partitioning, the balancing methods, the model development, the cross-validated hyperparameter optimisation, the calibration and the final blind-holdout evaluation. The source dataset contains 200 observations and the target dataset contains 1,659 observations, with 10 common attributes. Non-CKD was coded as 0 and CKD as 1. The target dataset was divided by stratified sampling with random_state = 42 into a 10% adaptation and calibration subset and a 90% blind holdout subset. The selected source-domain models were adapted using only the target calibration subset. The blind holdout was reserved exclusively for the final evaluation. RandomizedSearchCV with five-fold cross-validation was used for source-domain hyperparameter optimisation. The blind target holdout was not used for model fitting or hyperparameter selection. Missing values were handled through imputation and the features were standardised using StandardScaler. Class-balancing procedures were applied only to the training and adaptation data. The revised methodology states explicitly that the blind holdout data were not used for adaptation, threshold selection or any evaluation before the final test. These revisions ensure that the reported performance metrics come from an independent blind target-domain evaluation and make the experimental procedure reproducible and transparent.

Comment A8. Explain whether models were selected using cross-validation and whether the blind test set was used only after the pipeline was finished. Describe how you addressed optimistic model-selection bias. Use a suitable resampling method and report distributions or confidence intervals.

Response. Model selection was performed using five-fold stratified cross-validation with randomized hyperparameter search on the source-domain data. The target dataset was then divided into a 10% adaptation and calibration subset and a 90% blind holdout subset. The blind holdout was used only for the final evaluation. It was not used for model fitting, hyperparameter tuning, adaptation or threshold selection. To reduce the risk of optimistic model-selection bias, model development and hyperparameter selection were kept separate from the final blind evaluation. We have clarified this procedure in the revised Methods section. We also report 95% bootstrap confidence intervals from 2,000 resamples for the principal performance measures. We acknowledge that the relatively small number of non-CKD cases limits the stability of the performance estimates. The revised manuscript therefore avoids interpreting a single holdout result as evidence of stable clinical performance and identifies repeated resampling and independent external validation as necessary future work.

Comment A9. The manuscript does not provide enough information to reproduce the experiments. Report search spaces, optimisation strategy, final hyperparameters, software versions, environment, hardware and random states, and place the code in a stable public repository. Revise the Data Availability Statement.

Response. We have expanded the revised manuscript to provide the information necessary to reproduce the analysis. We now report the hyperparameter search spaces, the RandomizedSearchCV optimisation strategy, the final selected hyperparameters, the software and package versions, the computational environment, the hardware specifications and the random-state settings. The source-domain models were optimised using stratified five-fold cross-validation with a fixed random state of 42. We have clarified the complete analytical workflow, including preprocessing, dataset partitioning, class balancing, target-domain adaptation, threshold selection, metric calculation and explainability analyses. The final blind target-domain holdout was kept separate from model development and selection. The analysis code is provided through a stable public repository containing separate scripts and notebooks for preprocessing, dataset splitting, balancing, model training, hyperparameter tuning, evaluation, table and figure generation, and explainability analyses. The Data Availability Statement has been revised to distinguish the publicly available original datasets, the processed and derived data, the generated analytical outputs and the code required to reproduce the reported results.

Comment A10. Do not base claims of superiority on small differences in single point estimates. Check consistency across repeated samples and seeds. Use a statistical method suited to paired model-evaluation data and acknowledge uncertainty.

Response. We agree that superiority should not be inferred from isolated point estimates. We have revised the interpretation so that no classifier or balancing technique is described as unequivocally superior on the basis of a single metric. The revised analysis reports 95% bootstrap confidence intervals for the principal performance measures and considers performance across sensitivity, specificity, PPV, NPV, balanced accuracy, F1-score and MCC. We also performed formal pairwise comparisons using McNemar's test with Holm adjustment for multiple comparisons, using the paired predictions generated on the same blind-test observations. These results are reported in the revised manuscript and are used to distinguish statistically supported differences from differences based only on point estimates. The Discussion now acknowledges uncertainty and avoids interpreting a higher accuracy or F1-score alone as evidence of overall superiority. Although ETC obtained the highest accuracy and sensitivity, its specificity was substantially lower than that of the other classifiers. MLP achieved the highest balanced accuracy and MCC among the evaluated models, although its specificity also remained limited. The results therefore indicate different performance trade-offs rather than a universally superior classifier.

Comment A11. The explainability analyses need better methodological reporting and more careful interpretation. State the final model and dataset used for each explanation, the choice of background samples, whether calibration or blind-test data were used, and whether feature rankings were stable.

Response. We have revised the XAI methodology and interpretation to provide greater methodological detail and to avoid overstating the clinical significance of the explanations. We now identify the RO+TL+ETC model explicitly as the final model used for the explainability analyses. SHAP analyses were performed on the 90% blind-test holdout dataset for global and class-specific feature interpretation. LIME explanations were generated for observations from the blind-test holdout, using the adaptation-training subset as the reference and background data. Permutation importance was calculated on the blind-test holdout using the F1-score as the evaluation metric. We have clarified that the blind-test dataset was not used for model training, hyperparameter tuning or threshold selection. The XAI results are now described as model-specific feature contributions rather than as evidence of causal relationships, external validity or clinical validity. We also state that feature-ranking stability across independent resamples was not formally assessed, and therefore no claim of ranking stability is made. The Discussion clarifies that agreement between the identified features and established CKD-related variables does not demonstrate causality, external validity or clinical readiness. Given the low specificity observed in the blind-test evaluation, the explainability results are presented as a means of understanding model behaviour rather than as support for clinical deployment.

Comment A12. All equations and technical expressions should be checked independently. There were issues with the XGBoost objective function notation and with non-standard terms such as "SM", "roc-auci" and "over sounding".

Response. We have independently rechecked all equations and technical expressions. Equation (7) has been rewritten using the standard regularized XGBoost objective, and every symbol is now defined explicitly. The inaccurate and non-standard terms have been corrected throughout the text, tables and figures. "SM" has been replaced with "SMOTE", "roc-auci" with "ROC-AUC", and "Over sounding" with "Oversampling".

Comment A13. The manuscript needs a thorough revision for language, structure and scientific clarity. Shorten the Introduction and Literature Review, remove repeated material, sharpen the problem statement and check all citations.

Response. We have revised the manuscript thoroughly to improve its language, structure, readability and scientific clarity. Grammatically incorrect, repetitive and unclear sentences have been rewritten. The Introduction and Literature Review have been streamlined by removing repeated general descriptions of CKD and by focusing on the methodological motivation for the study. Duplicate material in the literature review has been removed. The problem statement has been revised to address the specific challenges investigated here, namely the severe class imbalance, the cross-dataset target-domain adaptation, the need for model interpretability and the limitations of the available CKD datasets. We have reviewed the in-text citations against the reference list and corrected incomplete or incorrectly formatted references. Relevant methodological references, including the foundational SMOTE study by Chawla et al. (2002), have been added. The author-year designations and citation consistency have also been checked throughout.

Comment A14. Rewrite the abstract and conclusion only after verifying the complete analysis. The abstract should include the corrected values and report specificity. The conclusion should state strengths and limitations without unsupported claims.

Response. We agree, and we rewrote the abstract and conclusion after the complete analysis had been rerun and verified. The abstract now reports the corrected performance values and includes specificity for the non-CKD class. The conclusion states the overall findings and the limitations of the study, and no longer claims clinical effectiveness given the low performance on the minority class.

Comment A15. Include a detailed point-by-point response, show where each change was made, and submit a marked version, a clean version, corrected tables and figures, updated supplementary materials, and full data and code availability details. Confirm that all reported numbers have been independently checked.

Response. This letter provides a point-by-point response to every reviewer and editorial comment and indicates where each change was made in the revised manuscript. No comment has been left unanswered. We are submitting a marked version of the manuscript with all changes highlighted, a clean version, the corrected tables and figures, and the updated supplementary materials. The Data Availability Statement distinguishes the original public datasets, the processed data, the derived analytical outputs and the code required to reproduce the reported results. Regarding the reanalysis, the complete experiment was rerun using the finalised pipeline. The source-domain models were selected by stratified five-fold cross-validation with randomized hyperparameter search. The target dataset was then split into a 10% adaptation and calibration subset and a 90% blind holdout subset, and the blind holdout was used only for the final evaluation. Every value reported in the abstract, main text, tables, figures and conclusion was regenerated from this single final analysis. We confirm that all reported numbers have been independently checked. The TP, TN, FP and FN counts were extracted from the final blind-holdout predictions, and accuracy, sensitivity, specificity, precision, F1-score and balanced accuracy were recalculated from those counts and cross-checked against the tables and the confusion matrices in Fig. 5.

Comment A16. Being invited to revise does not guarantee acceptance. The manuscript may be rejected if the conflicting performance results, the leakage-free and reproducible analysis, or the clinical claims are not resolved.

Response. We understand that the invitation to revise does not guarantee acceptance, and we welcome further expert review. We have addressed the three decisive issues. First, the conflicting performance results were resolved by rerunning the complete analysis, so the abstract, tables, figures and conclusions now derive from one final set of predictions. Second, the analysis is leakage-free and reproducible. The 90% blind holdout was excluded from preprocessing, balancing, hyperparameter tuning, adaptation and threshold selection. The search spaces, selected hyperparameters, random seeds, software versions and computational environment are now reported. Third, the clinical claims were revised to match the evidence. The framework is presented as an exploratory cross-dataset evaluation rather than a deployable screening system, and the low specificity is stated explicitly in the abstract, results, discussion, limitations and conclusion.

Comment A17. We appreciate your efforts on this study and hope these comments help you create a more technically sound, reproducible and clearly interpreted manuscript.

Response. We thank the Editor for the constructive assessment. The comments substantially improved the technical soundness, the reproducibility and the interpretation of the manuscript.

PART B. RESPONSE TO REVIEWER #1

We thank Reviewer #1 for the thorough and constructive evaluation of our manuscript. The detailed comments and the suggested references have substantially helped us improve the mathematical formulation, the methodological description and the empirical validation of our hybrid framework.

Comment R1. Mathematical contradiction. The metrics reported in Tables 3 and 5 (91.83% accuracy, 100% recall for GBC) are inconsistent with the confusion matrices in Fig. 5, which yield 84.61% accuracy and 89.88% recall.

Response. We thank the reviewer for identifying this inconsistency. We agree that the previously reported values for GBC were not consistent with the confusion matrix, which indicates an error in the earlier result-generation pipeline. We reran the complete 10% target-domain adaptation experiment using the corrected and leakage-controlled pipeline. The target dataset is divided into 10% adaptation and calibration data and 90% completely blind holdout data, with threshold selection performed on the adaptation and calibration set only. The final metrics and confusion matrices are now generated from the same holdout predictions, which ensures mathematical consistency among Tables 3, 5 and Fig. 5. Under the corrected pipeline RO+TL+ETC is the best-performing model according to the reported accuracy criterion. On the 1,494-sample blind target holdout it achieved 91.97% accuracy, 92.24% precision, 99.64% recall, 95.80% F1-score and 67.52% ROC-AUC. Its confusion matrix contains TN = 7, FP = 115, FN = 5 and TP = 1,367, which reproduces the reported accuracy and recall exactly. For GBC the corrected RO+TL+GBC result is 82.20% accuracy and 88.05% CKD recall, derived from the same holdout predictions. The previous statement identifying GBC as the best model has been removed. The revised analysis distinguishes the highest raw accuracy (RO+TL+ETC) from more balanced minority-class behaviour (RO+TL+MLP).

Comment R2. Lines 34-36. The abstract does not report specificity. Add specificity to the abstract.

Response. We agree that specificity must be reported, because it provides essential information about the ability of the model to identify the non-CKD class under substantial class imbalance. In the revised manuscript, specificity is reported in the Abstract alongside the other performance measures, and the limitation in non-CKD identification is acknowledged explicitly. Following the complete rerun, RO+TL+ETC achieved the highest accuracy (91.97%) with 99.64% CKD recall, but its specificity was only 5.74%, calculated directly from the confusion matrix (TN = 7, FP = 115). This shows that the high overall accuracy is strongly influenced by the predominance of CKD cases in the blind holdout set and should not be interpreted as balanced diagnostic performance. All specificity values reported in the manuscript are calculated directly from the corresponding confusion matrices. We have also added Table 7, which reports specificity for the RO+TL data-balancing technique.

Comment R3. Misleading terminology. Using about 10% of the target dataset for calibration is standard transfer learning, not few-shot learning.

Response. We agree that describing the use of 10% of the target dataset as few-shot learning could be misleading. We have replaced "few-shot learning", "few-shot personalization" and "few-shot adaptation" with "cross-dataset target-domain adaptation", "target-domain adaptation" and "target adaptive learning" as appropriate. The experimental framework uses the UCI CKD dataset as the source domain and a 10% subset of the target CKD dataset for model adaptation, while the remaining 90% is retained as a blind holdout set. The revised terminology reflects the experimental design accurately and no longer implies an extremely low-shot setting. We have revised the corresponding research question, objectives, methodology, literature comparison, discussion and contribution statements for consistency. The scientific contribution is now presented as a cross-dataset target-domain adaptation framework with limited target data, rather than as conventional few-shot learning.

Comment R4. ROC-AUC interpretation is inadequate. A ROC-AUC of 62.05% is barely above random guessing and indicates poor discriminative ability.

Response. We agree that reporting ROC-AUC as a single percentage without explaining its discriminatory meaning can be misleading, particularly under substantial class imbalance. The revised manuscript clarifies the interpretation of ROC-AUC and reports it together with the other evaluation metrics. Following the rerun, the selected RO+TL+ETC model achieved a ROC-AUC of 67.52% on the independent 90% blind target holdout, and the values across the RO+TL classifiers range from 0.56 to 0.68. These values are above the 50% random-classifier reference but indicate only limited to moderate discrimination across probability thresholds. They are not interpreted as evidence of strong class separation. We revised the Abstract and the Results and Discussion sections accordingly and distinguished ROC-AUC explicitly from the high accuracy and recall values. This clarification also acknowledges that the high accuracy is substantially affected by the imbalanced class distribution and by the limited ability of the model to identify non-CKD cases.

Comment R5. Clinical non-viability. All models exhibit critically low specificity, so 73 to 99% of healthy patients would be misclassified as having CKD. This renders the models unsuitable for clinical screening.

Response. We agree that specificity is a critical metric for CKD screening, because it reflects the ability of the model to identify non-CKD individuals and cannot be inferred from accuracy or recall. We re-evaluated the target-domain adaptation experiment completely using the corrected pipeline. The revised experiment uses 10% of the target-domain dataset for model adaptation and retains the remaining 90% as an independent blind holdout set. All performance metrics, including specificity, are now calculated directly from the predictions on this blind holdout set, which ensures consistency between the reported metrics and the confusion matrices. We have introduced Table 7, "Specificity Analysis of RO+TL Data Balancing Technique", which reports the specificity of the RO+TL models. The revised specificity values are 21.31% for DTC, 5.74% for ETC, 17.21% for RFC, 16.39% for GBC, 17.21% for XGBC and 25.41% for MLP. These values confirm a strong tendency toward CKD prediction and a limited ability to classify non-CKD cases correctly. Although RO+TL+ETC achieved the highest overall accuracy (91.97%) and a very high CKD recall (99.64%), its specificity of 5.74% corresponds to a false-positive rate of approximately 94.26% among non-CKD cases. We have therefore revised the Discussion, Limitations and Conclusions to state that the framework is unsuitable for clinical screening in its current form and that substantially more non-CKD samples are required before deployment can be considered.

Comment R6. Incomplete reporting. No hyperparameter values, hardware specifications, cross-validation strategies or statistical significance tests are provided.

Response. We agree that complete reporting of the model configuration, the hyperparameter optimisation procedure, the computational environment, the validation strategy and the statistical analysis is essential for reproducibility. We have expanded the experimental setup and added reproducibility information in section 4.2. The revised methodology reports the hyperparameter search spaces for all classifiers, the cross-validation strategy used during source-domain model selection, the data-balancing procedures, the target-domain adaptation protocol, the decision-threshold optimisation procedure and the final blind-holdout evaluation protocol. Hyperparameters were optimised using RandomizedSearchCV with five-fold cross-validation and 20 randomly sampled parameter combinations for each balancing-technique and classifier configuration, using balanced accuracy as the optimisation criterion. A fixed random seed of 42 was used. The source-domain models were then adapted using 10% of the target-domain data, while the remaining 90% was preserved as an independent blind holdout set. We have added the computational environment, including the CPU, RAM, operating system, Python version and library versions. The revised manuscript also reports the statistical analysis used to quantify uncertainty and to assess differences between model predictions.

Comment R7. Citation error. The reference "Almasoud & E, 2019" is incorrectly formatted.

Response. We have corrected this reference. The second author's name was incomplete because of an error in the Mendeley record. The reference-list entry now reads Almasoud, M., and Ward, T. E. (2019), and the in-text citation now reads (Almasoud and Ward, 2019). We have also corrected the record in our reference manager so that the full author name is retained.

Comment R8. Lines 44-46. "one point five million" should be "1.5 million", and "humans" is awkward.

Response. We have revised the statement to improve numerical consistency, readability and academic language. "one point five million" has been changed to "1.5 million" and "humans" has been replaced with "people".

Comment R9. Lines 52-53. Awkward phrasing regarding the sixth cause of death.

Response. We agree that the original sentence was awkward and did not convey the intended meaning clearly. We have revised the sentence to improve its grammatical structure, clarity and academic tone while preserving the intended meaning.

Comment R10. Line 58. "exposure to harmful environmental phenomena" uses a plural as a singular.

Response. Although "phenomena" is the correct plural of "phenomenon", we agree that the expression could be clearer in the context of environmental exposure. We have revised the phrase to "exposure to harmful environmental factors" to improve clarity and scientific precision.

Comment R11. Line 114. "Over sounding and Intersecting Samples" should be corrected, and the problem statement lacks specificity.

Response. We have corrected the typographical error and revised the subsection heading to "Oversampling and Intersecting Samples". We have also revised the problem statement to describe the research problem more specifically, including the class imbalance, the limited target-domain samples, the environmental exposure information and the need for interpretable predictions.

Comment R12. Lines 117-126. The problem statement is vague and generic and does not address the CKD datasets, the severity of the imbalance or the technical challenges in Section 1.1.

Response. We agree that the previous problem statement did not connect the research gaps to the characteristics and limitations of the CKD datasets used here. We have rewritten it to describe explicitly the severe class imbalance in the target CKD data, the challenges of adapting a model across heterogeneous source and target datasets, and the need for transparent and interpretable predictions. We have incorporated the dataset limitations and the technical challenges discussed in Section 1.1. These revisions provide a more specific motivation for the methodology and establish the relationship between the identified limitations and the objectives of the study.

Comment R13. Line 44. The "a" and "b" designations for Ortiz et al., 2026a and 2026b should be checked against the reference list.

Response. We cross-checked both in-text citations against the corresponding reference-list entries. The two entries were duplicate records of the same article rather than two separate publications. We have merged them into a single reference, Ortiz et al. (2026), removed the duplicate entry from the reference list and updated both in-text citations accordingly.

Comment R14. Line 127. The foundational SMOTE paper (Chawla et al., 2002) is absent from the reference list.

Response. We agree that the original SMOTE methodology must be cited, because SMOTE is one of the class-imbalance handling techniques evaluated in this study. We have added Chawla et al. (2002) to the reference list and cited it at the first relevant discussion of SMOTE. This properly acknowledges the methodological foundation of the SMOTE-based balancing approaches used in our experiments.

Comment R15. Minor issue, citation order. The Ortiz et al. 2026a and 2026b designations should be checked.

Response. As noted in our response to Comment R13, the 2026a and 2026b entries were duplicate records of the same article. They have been merged into a single reference, Ortiz et al. (2026). The duplicate was removed from the reference list and both in-text citations were updated to match. No changes to the scientific content or the results were required.

Comment R16. Missing citation. Chawla et al. (2002) must be added and cited.

Response. As noted in our response to Comment R14, Chawla et al. (2002) has been added to the reference list and cited at the first relevant discussion of SMOTE.

Comment R17. Literature review, citation error. Line 1091 appears as "Almasoud, M., & E, T. (2019)".

Response. As noted in our response to Comment R7, the incomplete author record has been corrected. The reference now reads Almasoud, M., and Ward, T. E. (2019), and the in-text citation reads (Almasoud and Ward, 2019). The record in our reference manager has also been corrected.

Comment R18. Lines 240-246. The description of XAI appears twice with slight variations.

Response. We agree that the repeated text was an inadvertent copy-paste error. We reviewed the affected section and removed the duplicate lines, retaining only the original description of explainable artificial intelligence.

Comment R19. Lines 180-205 and 213-253 contain redundant descriptions of CKD as a disease.

Response. We agree that general descriptions of CKD were unnecessarily repeated after being established in the Introduction. We have removed the duplicated disease-background material from the "Machine Learning in CKD Prediction" and "Explainable AI in CKD Risk Prediction" sections. These sections now focus on machine-learning algorithms, predictive performance, class-imbalance and generalization challenges, XAI methods and the findings of prior CKD prediction studies. We have also consolidated the repetitive descriptions of SHAP and LIME and strengthened the concluding research-gap discussion.

Comment R20. Missing recent citations. Expand the discussion of the 2025 to 2026 work and compare it more directly with your methodology.

Response. We agree that the previous literature review did not sufficiently discuss or compare the 2025 to 2026 studies. We have expanded the discussion of Elshewey et al. (2025), Rezk et al. (2025a, 2025b) and Gupta et al. (2026), describing their predictive and explainability approaches and comparing them with the methodology adopted here. We have added a synthesis that emphasises the methodological distinction between recent within-dataset CKD prediction studies and our cross-dataset target-domain adaptation framework. The revised text highlights our evaluation of seven balancing configurations and six classifiers, the 10% target-domain adaptation, the 90% blind target-domain evaluation and the integration of XAI techniques.

Comment R21. Citation formatting. "Khandoker et al. (2024)" at Line 217 appears to be an error, since Khandoker is not the first author.

Response. We have corrected this citation. The cited work is Ghosh and Khandoker (2024), in which Khandoker is the second author. The in-text citation now reads Ghosh and Khandoker (2024) and matches the reference-list entry. We have also rechecked the remaining in-text citations against the reference list.

Comment R22. Methodology. Equation (7) contains errors. The symbol n is used both for the loss function and for the number of input features, and the regularization term is misstated.

Response. We agree that the original formulation incorrectly used n in connection with the loss function and created a conflict with the existing notation. We have corrected Equation (7) using the standard regularized XGBoost objective, in which the sum of the per-sample losses is combined with the regularization term for each tree. All parameters and symbols are now defined explicitly.

Comment R23. Line 441. "roc-auci" should be "ROC-AUC".

Response. We have corrected this term to ROC-AUC. It now appears at line 482 of the revised manuscript.

Comment R24. "SM" is used as a non-standard abbreviation for SMOTE.

Response. We agree that SMOTE is the accepted abbreviation. We have replaced all instances of "SM" with "SMOTE" in the text, the tables and the figures.

Comment R25. Missing hyperparameter details. Add a supplementary table listing all final hyperparameters for all six models.

Response. We have revised the Methods section to report the hyperparameter optimisation strategy, the search procedure, the cross-validation settings, the optimisation criterion and the random seed. RandomizedSearchCV was used to evaluate 20 randomly sampled configurations with five-fold stratified cross-validation, shuffled folds and random seed 42, using balanced accuracy as the selection criterion. We have added Table 9, which reports the final optimal hyperparameters and the corresponding cross-validation balanced accuracy for all six classifiers under the RO+TL balancing strategy. The predefined search ranges are reported in Table 8.

Comment R26. Data leakage risk. Clarify whether the StandardScaler was fitted only on the training and calibration set, and whether the 10% calibration set or the full target dataset was used for scaling.

Response. We have clarified the preprocessing procedure. The source and target datasets were preprocessed before the target-domain adaptation stage. Missing values were handled through imputation and the features were standardised using StandardScaler before adaptation. The target-domain adaptation procedure was then performed on the preprocessed target dataset, with 10% of the target data used for adaptation and the remaining 90% reserved as a blind holdout. We have stated this sequence explicitly in the Methods section, so that the preprocessing stage is clearly distinguished from the subsequent adaptation and blind evaluation stages. We have also stated explicitly that the blind holdout labels were not used at any stage of model development, adaptation or threshold selection.

Comment R27. Hyperparameter tuning is not described. Add a subsection describing the search strategy, the range of values and the final selected parameters.

Response. We agree that the original manuscript did not provide adequate information on the optimisation strategy, the search space, the cross-validation procedure or the selected hyperparameters. We have added a dedicated subsection on hyperparameter tuning. The revised methodology clarifies that RandomizedSearchCV, rather than GridSearchCV, was used to evaluate 20 randomly sampled hyperparameter configurations for each classifier with five-fold stratified cross-validation. The folds were shuffled using a fixed random seed of 42 and balanced accuracy was the model-selection criterion. The predefined search ranges for all six classifiers and the final selected hyperparameters for the RO+TL configuration are reported in Tables 8 and 9. We have also revised the literature-review wording to avoid implying that GridSearchCV was used in our experiments.

Comment R28. Results. Table 3 metrics are inconsistent with Fig. 5. This must be resolved before resubmission.

Response. This inconsistency has been resolved by the complete rerun described in our response to Comment R1. The final metrics and confusion matrices are now generated from the same blind-holdout predictions, so Tables 3, 5 and Fig. 5 are internally consistent. RO+TL+ETC achieves 91.97% accuracy, 92.24% precision, 99.64% recall, 95.80% F1-score and 67.52% ROC-AUC on the 1,494-sample blind holdout, with TN = 7, FP = 115, FN = 5 and TP = 1,367. The corrected RO+TL+GBC result is 82.20% accuracy and 88.05% CKD recall.

Comment R29. GBC is not the best model based on Fig. 5. ETC outperforms GBC on accuracy, recall and F1-score. Correct the "best model" claim throughout.

Response. We agree. The claim has been corrected throughout the manuscript. Under the corrected pipeline, RO+TL+ETC is identified as the best-performing configuration according to the predefined accuracy criterion, and all statements identifying GBC as the best model have been removed. The revised analysis also distinguishes the highest raw accuracy (RO+TL+ETC) from the more balanced minority-class behaviour of RO+TL+MLP, rather than describing any single configuration as the overall champion.

Comment R30. The 100% recall claim is false. No model achieves 100% recall.

Response. We agree that the manuscript contained inaccurate statements reporting 100% recall. These resulted from an earlier version of the experimental results and were not consistent with the corrected evaluation. We have reviewed the manuscript, tables, figures and discussion and removed all unsupported 100% recall claims. Every reported recall value now corresponds to the results generated by the corrected pipeline. The previously reported GBC result has also been corrected, and the final selected RO+TL+ETC model achieves 99.64% recall on the 90% blind target-domain holdout. The recall values in the text, tables, figures and discussion are internally consistent.

Comment R31. Specificity is omitted from Tables 3 and 5. Add specificity and discuss its clinical implications.

Response. We agree, and specificity is now reported. All performance metrics, including specificity, are calculated directly from the predictions on the blind holdout set, which ensures consistency with the corresponding confusion matrices. We have added Table 7, "Specificity Analysis of RO+TL Data Balancing Technique". The revised specificity values are 21.31% for DTC, 5.74% for ETC, 17.21% for RFC, 16.39% for GBC, 17.21% for XGBC and 25.41% for MLP. These values confirm that the models have a strong tendency toward CKD prediction and a limited ability to classify non-CKD cases correctly. Although RO+TL+ETC achieved the highest overall accuracy (91.97%) and a very high CKD recall (99.64%), its specificity of 5.74% corresponds to a false-positive rate of approximately 94.26% among non-CKD cases. The clinical implications are now discussed explicitly in the Results, Discussion and Limitations sections.

Comment R32. Key Observation 1 at Line 686 is incorrect. It reports RO+TL+GBC as best with 91.83% accuracy and 100% recall.

Response. We agree that the original statement incorrectly identified RO+TL+GBC as the best overall configuration and reported an erroneous 100% recall value. We have corrected this observation using the results of the revised pipeline. The revised analysis identifies RO+TL+ETC as the best-performing configuration according to the predefined accuracy criterion, achieving 91.97% accuracy, 99.64% recall, 92.24% precision, 5.74% specificity, 52.69% balanced accuracy and 67.52% ROC-AUC on the 90% blind target-domain holdout. The 100% recall claim has been removed and the associated text and figure results have been revised for complete consistency. We also acknowledge explicitly that the very high recall is accompanied by very low specificity and should not be interpreted as uniformly strong clinical discrimination.

Comment R33. No statistical significance testing. Apply McNemar's test for pairwise classifier comparisons and report p-values.

Response. We have incorporated McNemar's test to compare the paired predictions of the six classifiers on the same 90% blind target-domain holdout under the RO+TL configuration. Because 15 pairwise comparisons were performed, Holm's correction was applied to the resulting p-values. The analysis identified statistically significant differences in 12 of the 15 pairwise comparisons after correction. Three comparisons were not statistically significant, namely DTC versus XGBC, RFC versus MLP and XGBC versus MLP. The data therefore do not support the conclusion that all classifiers perform equivalently. ETC was selected according to the predefined accuracy criterion, and its paired predictions differed significantly from those of all other evaluated classifiers after Holm correction. The Methods and Results sections have been revised accordingly and the complete pairwise statistical results are provided in Table 11.

Comment R34. Hardware specifications are missing from Table 6. Add a computational environment subsection.

Response. We agree that reporting training and prediction times without specifying the hardware and software environment limits reproducibility. We have added a dedicated "Computational Environment" subsection before the Experimental Setup section. The revised manuscript reports the CPU model, the available RAM, the operating system, the Python version and the versions of the principal computational libraries, including scikit-learn, imbalanced-learn, XGBoost, NumPy, Pandas, Matplotlib and Seaborn. The training and prediction times measured in this environment are reported in Table 12 of the revised manuscript.

Comment R35. SHAP figure labels are inconsistent with the text. Ensure consistent naming between figures and text.

Response. We have revised the whole manuscript to ensure consistency between the figures and the text. The feature names now appear as "diastolicbp" and "fastingbloodsugar" throughout the text, so that they match the labels used in Figs. 7, 8A and 8B.

Comment R36. Discussion contradicts results. The statement that Gradient Boosting performed best is inconsistent with Fig. 5.

Response. We agree that the previous wording incorrectly described Gradient Boosting as the best-performing classifier. We have corrected the Discussion to identify the Extra Trees Classifier combined with Random Oversampling and Tomek Links as the selected configuration under the predefined accuracy criterion. We have also revised the discussion to report its corresponding performance and to acknowledge its substantially low specificity, so that the interpretation is consistent with the complete set of evaluation metrics.

Comment R37. Clinical applicability claims are overstated. With very low specificity the model misclassifies most healthy patients.

Response. We agree that the previous statement overstated the clinical applicability of the framework. Given the low specificity observed across the evaluated models, the framework should not be presented as suitable for clinical screening or as capable of directly supporting clinical decision-making. We have revised the Discussion to acknowledge this limitation explicitly and to state that substantially more non-CKD samples are required before clinical deployment can be considered. We have also clarified that, despite the high sensitivity observed for CKD cases, the framework is research-oriented and is not intended for standalone clinical deployment.

Comment R38. RQ3 few-shot claim is unsupported. Replace "few-shot" with "limited-sample domain adaptation".

Response. We agree that describing the use of 10% of the target-domain dataset as few-shot learning was not methodologically appropriate and could overstate the nature of the adaptation setting. We have removed the term "few-shot" throughout the manuscript and replaced it with "limited-sample domain adaptation" or "limited-sample target-domain adaptation" as appropriate. We have also revised RQ3 and the corresponding Discussion statements to describe the experimental setting accurately without implying a formal few-shot learning paradigm.

Comment R39. The limitations section is inadequate. It omits the extremely small non-CKD sample, the low specificity, the absence of external validation and the inconsistency between Table 3 and Fig. 5.

Response. We agree that the previous limitations section did not sufficiently address the methodological and clinical constraints of the study. We have substantially revised Section 5.4, "Limitations of the Proposed Work". The revised section now addresses the extremely small number of non-CKD cases in the target dataset, with 13 non-CKD versus 152 CKD cases in the 10% adaptation subset and 122 non-CKD versus 1,372 CKD cases in the 90% blind holdout. It also addresses the low specificity and the resulting high false-positive rate, the consequent conclusion that the framework is not suitable for standalone clinical screening, the absence of external validation on a third independent dataset, the use of only 10% of the target-domain data for adaptation, and the absence of multimodal, deep-learning and transformer-based comparisons. In addition, the experimental analysis was rerun using the corrected and consistent pipeline, so the reported performance metrics, confusion matrices and class-specific measures are mutually consistent and the earlier inconsistency between the tables and the figures no longer arises. Rather than presenting the framework as clinically deployable, the revised manuscript states that substantially larger and clinically representative non-CKD samples, improved class-wise discrimination, calibration and independent external validation are required before clinical deployment can be considered.

Comment R40. Future work lacks specificity. Provide concrete directions.

Response. We have revised the Future Work section to provide concrete and technically specific directions. The revised manuscript proposes GAN-based generation of synthetic non-CKD samples, cost-sensitive learning as an alternative to oversampling, and validation using a third independent dataset such as NHANES. We also identify improving specificity while maintaining CKD sensitivity as a key objective for future investigation.

Comment R41. Conclusions overstate the findings. They repeat the incorrect accuracy and recall values and the claim of clinical usefulness.

Response. We have revised the Conclusions section completely. The previously reported 91.83% accuracy and 100% recall claims have been removed, and the conclusions now correspond to the corrected experimental results. The revised conclusion identifies RO+TL+ETC as the best-performing model in the 10% target-adaptation experiment and acknowledges explicitly the low specificity, the high false-positive rate, the limited number of non-CKD cases and the lack of external validation. We have also removed the unsupported claim that the framework is capable of assisting physicians in clinical decision-making and clarified that it is not clinically deployable at this stage.

Comment R42. Citation errors. Review all references for completeness and correct formatting, including Almasoud and the Rezk et al. 2025a and 2025b designations.

Response. We have reviewed the reference list and the in-text citations throughout the manuscript. The incomplete author information for Almasoud et al. (2019) has been corrected to give the complete author names in the journal's reference style. We checked the citations for Rezk et al. (2025a, 2025b) and found that the a and b entries pointed to the same article. They have been merged into a single reference, Rezk et al. (2025). The passage previously attributed to Rezk et al. (2025a), which describes an Extra Trees model with feature selection and SHAP interpretation, in fact refers to Elshewey et al. (2025), and the attribution has been corrected. The remaining references were reviewed for completeness, consistency, author names, publication year, journal information and in-text citation matching.

CLOSING

We thank the Editor and Reviewer #1 again for the detailed and constructive comments. Every comment has been addressed, and the revised manuscript reports a single, internally consistent, leakage-controlled analysis with appropriately moderated conclusions.

Attachment Submitted filename: Response Letter.docx

# Peer review: Decision Letter 1

**DOI:** 10.1371/journal.pone.0362320.r003

**Authors:** Jordan Llego

1 Oct 2026

Dear Dr. Tanvir Anzum,

Thank you for submitting your manuscript to PLOS One. After careful consideration, we feel that it has merit but does not fully meet PLOS One’s publication criteria as it currently stands. Therefore, we invite you to submit a revised version of the manuscript that addresses the points raised during the review process.

Please submit your revised manuscript by Nov 15 2026 11:59PM. If you will need more time than this to complete your revisions, please reply to this message or contact the journal office at plosone@plos.org. When you're ready to submit your revision, log on to https://www.editorialmanager.com/pone/ and select the 'Submissions Needing Revision' folder to locate your manuscript file.

- A letter that responds to each point raised by the academic editor and reviewer(s). You should upload this letter as a separate file labeled 'Response to Reviewers'.
- A marked-up copy of your manuscript that highlights changes made to the original version. You should upload this as a separate file labeled 'Revised Manuscript with Track Changes'.
- An unmarked version of your revised paper without tracked changes. You should upload this as a separate file labeled 'Manuscript'.

If you would like to make changes to your financial disclosure, please include your updated statement in your cover letter. Guidelines for resubmitting your figure files are available below the reviewer comments at the end of this letter.

If applicable, we recommend that you deposit your laboratory protocols in protocols.io to enhance the reproducibility of your results. Protocols.io assigns your protocol its own identifier (DOI) so that it can be cited independently in the future. For instructions see: https://journals.plos.org/plosone/s/submission-guidelines#loc-laboratory-protocols. Additionally, PLOS One offers an option for publishing peer-reviewed Lab Protocol articles, which describe protocols hosted on protocols.io. Read more information on sharing protocols at https://plos.org/protocols?utm_medium=editorial-email&utm_source=authorletters&utm_campaign=protocols.

As the corresponding author, your ORCID iD is verified in the submission system and will appear in the published article. PLOS supports the use of ORCID, and we encourage all coauthors to register for an ORCID iD and use it as well. Please encourage your coauthors to verify their ORCID iD within the submission system before final acceptance, as unverified ORCID iDs will not appear in the published article. *Only*  the individual author can complete the verification step; PLOS staff *cannot*  verify ORCID iDs on behalf of authors.

We look forward to receiving your revised manuscript.

Kind regards,

Jordan Llego, PhD ELM, D. Hon. Ex., PhDN, RN

Academic Editor

PLOS One

Journal Requirements:

If the reviewer comments include a recommendation to cite specific previously published works, please review and evaluate these publications to determine whether they are relevant and should be cited. There is no requirement to cite these works unless the editor has indicated otherwise.

Please review your reference list to ensure that it is complete and correct. If you have cited papers that have been retracted, please include the rationale for doing so in the manuscript text, or remove these references and replace them with relevant current references. Any changes to the reference list should be mentioned in the rebuttal letter that accompanies your revised manuscript. If you need to cite a retracted article, indicate the article’s retracted status in the References list and also include a citation and full reference for the retraction notice.

Additional Editor Comments:

The revised manuscript exhibits significant improvement compared to the previous version. Specifically, the authors have rerun the analysis using a leakage-controlled pipeline, clarified the target-domain adaptation procedure, resolved inconsistencies in performance metrics and confusion matrices, expanded the reporting of class-specific performance measures, moderated clinical claims, enhanced reproducibility reporting, and revised the interpretation of the model’s relatively low specificity and discriminative performance.

The substantive concerns raised during the previous review have been addressed. The manuscript is now technically sound, statistically appropriate, adequately supported by the available data, and clearly written. The remaining issues are minor and should be readily addressed before the manuscript can proceed further.

Equation (7) should be revised so that N is explicitly defined as the number of samples, and \(l(s_i,\hat{s}_i)\) is clearly defined as the loss function. A thorough final proofread of the entire manuscript is recommended, including equations, mathematical notation, figure and table labels, abbreviations, references, typographical errors, and consistency of terminology. Given the extent of previous revisions and reanalysis, pay particular attention to ensuring that all numerical values in the Abstract, Results, tables, figures, Discussion, and Conclusion are fully consistent with the final analysis.

[Note: HTML markup is below. Please do not edit.]

Reviewers' comments:

Reviewer's Responses to Questions

**Comments to the Author**

Reviewer #1: All comments have been addressed

**********

2. Is the manuscript technically sound, and do the data support the conclusions?

Reviewer #1: Yes

**********

3. Has the statistical analysis been performed appropriately and rigorously?

Reviewer #1: Yes

**********

4. Have the authors made all data underlying the findings in their manuscript fully available?

The PLOS Data policy

Reviewer #1: Yes

**********

5. Is the manuscript presented in an intelligible fashion and written in standard English?

Reviewer #1: Yes

**********

Reviewer #1: The authors have addressed all substantive comments from the first round of review. The remaining issue (the definition of N in Equation (7)) is a minor typographical/notational correction that can be easily fixed.

I recommend that after the authors:

1. Correct the definition of N in Equation (7) to "N = number of samples" (with l(sᵢ, ŝᵢ) = loss function), and

2. Conduct a final proofread of the entire manuscript to catch any remaining typographical errors,

the manuscript should be accepted without further rounds of external review.

**********

what does this mean?). If published, this will include your full peer review and any attached files.

If you choose “no”, your identity will remain anonymous but your review may still be made public.

**Do you want your identity to be public for this peer review?**  For information about this choice, including consent withdrawal, please see our Privacy Policy

Reviewer #1: **Yes:**  Maurice Wanyonyi

**********

[NOTE: If reviewer comments were submitted as an attachment file, they will be attached to this email and accessible via the submission site. Please log into your account, locate the manuscript record, and check for the action link "View Attachments". If this link does not appear, there are no attachment files.]

To ensure your figures meet our technical requirements, please review our figure guidelines: https://journals.plos.org/plosone/s/figures

You may also use PLOS’s free figure tool, NAAS, to help you prepare publication quality figures: https://journals.plos.org/plosone/s/figures#loc-tools-for-figure-preparation.

NAAS will assess whether your figures meet our technical requirements by comparing each figure against our figure specifications.

# Author comment: Author response to Decision Letter 2

**DOI:** 10.1371/journal.pone.0362320.r004

12 Oct 2026

We sincerely thank the Editor and Reviewer for their thorough and constructive assessment of our manuscript. Their feedback has strengthened the positioning, methodological transparency, and presentation of the revised work. Below is a point-by-point response to all comments.

REVIEWER #1

Comment: The authors have addressed all substantive comments from the first round of review. The remaining issue (the definition of N in Equation (7)) is a minor typographical/notational correction that can be easily fixed. I recommend that the authors: 1. Correct the definition of N in Equation (7) to ‘N = number of samples’ (with l(si, s-hat-i) = loss function), and 2. Conduct a final proofread of the entire manuscript to catch any remaining typographical errors. The manuscript should be accepted without further rounds of external review.

Response: Thank you for your thorough and constructive review. We are pleased that the substantive comments from the first round have been fully addressed.

Regarding point 1: The definition of N in Equation (7) has been explicitly clarified in the manuscript. The notation now reads: N = number of samples, and l(si, s-hat-i) = loss function, which appear in the explanatory text immediately following Equation (7).

Regarding point 2: We have conducted a thorough final proofread of the entire manuscript. All typographical errors identified during this review have been corrected. We are confident that the manuscript is now ready for publication.

ADDITIONAL EDITOR COMMENTS

Comment 1: The revised manuscript exhibits significant improvement compared to the previous version. Specifically, the authors have rerun the analysis using a leakage-controlled pipeline, clarified the target-domain adaptation procedure, resolved inconsistencies in performance metrics and confusion matrices, expanded the reporting of class-specific performance measures, moderated clinical claims, enhanced reproducibility reporting, and revised the interpretation of the model’s relatively low specificity and discriminative performance.

Response: Thank you for the valuable feedback. We are grateful for the overall positive assessment of the updated analytical pipeline, consistency in performance measurements, expanded class-specific evaluation, reproducibility reporting, and the target-domain adaptation procedure. We have also tempered the clinical claims and explained the limitations related to low specificity and limited discriminative performance. These revisions reframe the framework as an exploratory cross-dataset machine-learning evaluation rather than a clinically deployable system.

Comment 2: The substantive concerns raised during the previous review have been addressed. The manuscript is now technically sound, statistically appropriate, adequately supported by the available data, and clearly written. The remaining issues are minor and should be readily addressed before the manuscript can proceed further.

Response: We thank the editor for this recognition. We appreciate the acknowledgment that the manuscript has improved in technical rigor, statistical analysis, evidential support, and clarity. We have carefully addressed all remaining minor issues in this revision.

Comment 3: Equation (7) should be revised so that N is explicitly defined as the number of samples, and l(si, s-hat-i) is clearly defined as the loss function.

Response: Thank you for this valuable observation. We have revised the explanation accompanying Equation (7) to explicitly define N as the number of samples and clarify the meaning of the loss function, the true label (actual target value), the predicted label (model output) for the i-th training example, the model parameters, and the regularization term. These definitions have been added immediately below Equation (7) to improve mathematical clarity and reproducibility.

Comment 4: A thorough final proofread of the entire manuscript is recommended, including equations, mathematical notation, figure and table labels, abbreviations, references, typographical errors, and consistency of terminology.

Response: Thank you for this recommendation. We have carefully proofread the revised manuscript to improve its overall accuracy, consistency, and readability. We reviewed the equations and mathematical notation, figure and table labels, abbreviations, references, and typographical errors. We also verified the consistency of key terminology throughout, particularly regarding target-domain adaptation, class-specific performance metrics, and explainable AI.

Comment 5: Given the extent of previous revisions and reanalysis, pay particular attention to ensuring that all numerical values in the Abstract, Results, tables, figures, Discussion, and Conclusion are fully consistent with the final analysis.

Response: Thank you for this important recommendation. We have carefully reviewed all numerical values throughout the revised manuscript to ensure consistency with the final analysis. We cross-checked the Abstract, Results, tables, figures, Discussion, and Conclusion, including the reported performance metrics, class distributions, and model comparisons. We also verified that the interpretation of results accurately reflects the final findings, particularly the distinction between high sensitivity and limited specificity under class imbalance. All discrepancies identified during this review have been corrected.

Attachment Submitted filename: Response Letter Final.docx

# Editor report: Decision Letter 2

**DOI:** 10.1371/journal.pone.0362320.r005

**Authors:** Jordan Llego

22 Oct 2026

An Explainable Machine Learning Framework for Cross-Dataset Chronic Kidney Disease Risk Prediction Using Hybrid Data Balancing

PONE-D-26-30448R2

Dear Dr. Tanvir Anzum,

We’re pleased to inform you that your manuscript has been judged scientifically suitable for publication and will be formally accepted for publication once it meets all outstanding technical requirements.

Within one week, you’ll receive an e-mail detailing the required amendments. When these have been addressed, you’ll receive a formal acceptance letter and your manuscript will be scheduled for publication.

An invoice will be generated when your article is formally accepted. Please note, if your institution has a publishing partnership with PLOS and your article meets the relevant criteria, all or part of your publication costs will be covered. Please make sure your user information is up-to-date by logging into Editorial Manager at Editorial Manager® and clicking the ‘Update My Information' link at the top of the page. For questions related to billing, please contact billing support.

If your institution or institutions have a press office, please notify them about your upcoming paper to help maximize its impact. If they’ll be preparing press materials, please inform our press team as soon as possible -- no later than 48 hours after receiving the formal acceptance. Your manuscript will remain under strict press embargo until 2 pm Eastern Time on the date of publication. For more information, please contact onepress@plos.org.

Kind regards,

Jordan Llego, PhD ELM, D. Hon. Ex., PhDN, RN

Academic Editor

PLOS One

Additional Editor Comments (optional):

After thoroughly evaluating the second revised manuscript and the accompanying point-by-point responses, it is evident that the authors have adequately addressed the substantive concerns raised in previous rounds of peer review. The revisions have substantially improved the methodological transparency, statistical rigor, reproducibility, and overall scientific presentation of the study.

In particular, the revised manuscript shows significant improvements in implementing a leakage-controlled analytical pipeline, clarifying the target-domain adaptation procedure, ensuring consistent performance metrics, and reporting class-specific predictive performance. The authors have also appropriately acknowledged the limitations associated with low specificity and discriminatory performance, ensuring that the conclusions remain proportionate to the available evidence and that the proposed framework is presented as an exploratory approach rather than a clinically validated decision-support system.

Furthermore, the remaining minor concerns regarding mathematical notation, particularly Equation (7), manuscript proofreading, and consistency of numerical reporting have been satisfactorily addressed. The reviewer’s recommendation for acceptance without further external review is duly acknowledged.

On this basis, the manuscript is technically sound, sufficiently supported by the reported findings, and consistent with PLOS ONE's scientific and editorial standards. No further scientific revisions or additional rounds of external peer review are deemed necessary.

The authors are congratulated on successfully completing the peer-review process. Appreciation is extended for their responsiveness to editorial and reviewer recommendations and for their contribution to research on explainable machine learning and chronic kidney disease risk prediction.

Reviewers' comments:

# Editor report: Acceptance letter

**DOI:** 10.1371/journal.pone.0362320.r006

**Authors:** Jordan Llego

PONE-D-26-30448R2

PLOS One

Dear Dr. Tanvir Anzum,

I'm pleased to inform you that your manuscript has been deemed suitable for publication in PLOS One. Congratulations! Your manuscript is now being handed over to our production team.

At this stage, our production department will prepare your paper for publication. This includes ensuring the following:

* All references, tables, and figures are properly cited

* All relevant supporting information is included in the manuscript submission,

* There are no issues that prevent the paper from being properly typeset

You will receive further instructions from the production team, including instructions on how to review your proof when it is ready. Please keep in mind that we are working through a large volume of accepted articles, so please give us a few days to review your paper and let you know the next and final steps.

Lastly, if your institution or institutions have a press office, please let them know about your upcoming paper now to help maximize its impact. If they'll be preparing press materials, please inform our press team within the next 48 hours. Your manuscript will remain under strict press embargo until 2 pm Eastern Time on the date of publication. For more information, please contact onepress@plos.org.

You will receive an invoice from PLOS for your publication fee after your manuscript has reached the completed accept phase. If you receive an email requesting payment before acceptance or for any other service, this may be a phishing scheme. Learn how to identify phishing emails and protect your accounts at https://explore.plos.org/phishing.

If we can help with anything else, please email us at customercare@plos.org.

Thank you for submitting your work to PLOS One and supporting open access.

Kind regards,

PLOS One Editorial Office Staff

on behalf of

Dr. Jordan Llego

Academic Editor

PLOS One
